# Highly compressible and anisotropic lamellar ceramic sponges with superior thermal insulation and acoustic absorption performances

**DOI:** 10.1038/s41467-020-17533-6

**Published:** 2020-07-24

**Authors:** Chao Jia, Lei Li, Ying Liu, Ben Fang, He Ding, Jianan Song, Yibo Liu, Kejia Xiang, Sen Lin, Ziwei Li, Wenjie Si, Bo Li, Xing Sheng, Dongze Wang, Xiaoding Wei, Hui Wu

**Affiliations:** 1 State Key Laboratory of New Ceramics and Fine Processing, School of Materials Science and Engineering, Tsinghua University, Beijing, 100084 China; 2 State Key Laboratory for Turbulence and Complex System, Department of Mechanics and Engineering Science, College of Engineering, Peking University, Beijing, 100871 China; 3 Beijing Innovation Center for Engineering Science and Advanced Technology, Peking University, Beijing, 100871 China; 4 Beijing Engineering Research Center of Mixed Reality and Advanced Display, School of Optics and Photonics, Beijing Institute of Technology, Beijing, 100081 China; 5 Advanced Materials Institute, Graduate School at Shenzhen, Tsinghua University, Shenzhen, 518055 China; 6 Department of Electronic Engineering, Beijing National Research Center for Information Science and Technology, Tsinghua University, Beijing, 100084 China; 7 Department of Energy and Power Engineering, Tsinghua University, Beijing, 100084 China

**Keywords:** Materials science, Soft materials

## Abstract

Advanced ceramic sponge materials with temperature-invariant high compressibility are urgently needed as thermal insulators, energy absorbers, catalyst carriers, and high temperature air filters. However, the application of ceramic sponge materials is severely limited due to their complex preparation process. Here, we present a facile method for large-scale fabrication of highly compressible, temperature resistant SiO_2_-Al_2_O_3_ composite ceramic sponges by blow spinning and subsequent calcination. We successfully produce anisotropic lamellar ceramic sponges with numerous stacked microfiber layers and density as low as 10 mg cm^−3^. The anisotropic lamellar ceramic sponges exhibit high compression fatigue resistance, strain-independent zero Poisson’s ratio, robust fire resistance, temperature-invariant compression resilience from −196 to 1000 °C, and excellent thermal insulation with a thermal conductivity as low as 0.034 W m^−1^ K^−1^. In addition, the lamellar structure also endows the ceramic sponges with excellent sound absorption properties, representing a promising alternative to existing thermal insulation and acoustic absorption materials.

## Abstract (web summary)

Temperature-invariant highly compressible ceramic sponges are attractive for thermal insulators and energy absorbers, but development is limited by complex preparation processes. Here the authors report large-scale fabrication of silica-alumina composite ceramic sponges via blow spinning and calcination.

## Introduction

In recent years, various sponge-like materials, including carbon nanotube aerogels^1,2^, biomass-derived aerogels^3–6^, graphene aerogels^7–12^, ceramic nanofiber aerogels^13–15^, and carbon nanofiber aerogels^16–18^, have received significant interest owing to their high compressibility and resilience under large deformation, which results from their porous three-dimensional network structures. Among them, ceramic sponge materials have attracted more interest owing to their lightweight feature, high specific surface area, low thermal conductivity, and excellent chemical and thermal stability^13,14,19,20^. By virtue of these characteristics, ceramic sponge materials have been widely used in a variety of fields, including thermal insulation, water treatment, as catalyst carriers, for energy absorption, and for high-temperature air filtration^13,21–24^. However, traditional ceramic sponge materials are usually prepared from ceramic oxides, such as silica nanoparticles, alumina nanolattices, and boron nitride sheets, and the inherent brittleness of ceramic materials severely limits their practical applications^25–27^. Therefore, it is urgent to develop ceramic sponge materials with excellent flexibility, high compressibility, as well as high and low-temperature resistance.

Various methods have been used to prepare compressible ceramic sponge materials. An effective method is direct assembly of ceramic nanofibers/microfibers into flexible ceramic sponges^28,29^. Ceramic nanofibers are usually prepared by electrospinning and are usually stacked into a random nonwoven structure owing to the direct spinning-deposition process. Although ceramic nanofiber-based sponges can be fabricated by reasonably designing the receiver, it is difficult to obtain real three-dimensional sponges with regular shapes^30,31^. In addition, sponges produced by direct electrospinning have no ordered structure, thus they have poor compression resistance. Another practical approach is chemical vapor deposition (CVD)^22,32,33^. For example, Xu et al.^32^ developed hexagonal boron nitride ceramic aerogels with nanolayered double-pane walls and hyperbolic architecture using a template-assisted and catalyst-free CVD method. The aerogels have Poisson’s ratio of −0.25 and a linear thermal expansion coefficient of −1.8 × 10^−6^ °C^−1^. The aerogels also exhibit robust mechanical and thermal stability, ultralow density, superelasticity, and ultralow thermal conductivity in vacuum and air. Although some highly compressible, high-temperature-resistant ceramic sponge materials have been fabricated using these aforementioned strategies, the preparation process is usually complicated and involves multiple steps, which greatly hinders their mass production and application. Therefore, preparing flexible ceramic sponges with temperature-invariant high compressibility with a facile, low-cost, scalable method is still very challenging.

In this work, we developed an anisotropic lamellar SiO_2_–Al_2_O_3_ composite ceramic sponge (SAC sponge) with temperature-invariant high compressibility using a sol-gel solution blow spinning technique followed by calcination. These SAC sponges have lamellar structure and exhibit high compressibility up to 80% strain and high compression fatigue resistance of 600 cycles at 50% strain. In addition, the layered structure and ceramic components provide the SAC sponges with robust fire resistance, temperature-invariant compression resilience from −196 °C to 1000 °C, and excellent thermal insulation property with a thermal conductivity as low as 0.034 W m^−1^ K^−1^. In addition, we also demonstrated that the lightweight SAC sponges with a density of 20 mg cm^−3^ and a thickness of 29 mm possess excellent sound absorption properties (NRC of 0.77). The developed anisotropic lamellar SAC sponges with temperature-invariant high compressibility represent a promising alternative to current brittle materials used in the fields of thermal insulation and acoustic absorption.

## Results

### Preparation of the SAC sponges

**Fig. 1:**
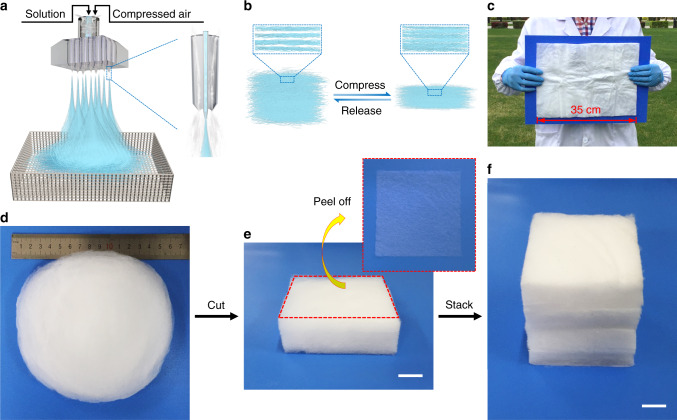
Preparation of SiO_2_–Al_2_O_3_ composite ceramic sponges (SAC sponges). **a** Schematic diagram of the design and preparation process of the SAC sponges. **b** Schematic to show the high compressibility of the SAC sponges. The SAC sponges possess anisotropic mechanical properties and high compressibility owing to the unique lamellar structure. **c** Photograph of a large SAC sponge sample to demonstrate the scalability of the preparation method. **d** Photograph of a typical circular SAC sponge, which has a diameter of 16 cm. **e** Photograph showing a square SAC sponge cut from the large sample in **d**. Scale bar in **e**, 1 cm. The inset in the top right corner is the photograph of a microfiber layer peeling off from the square SAC sponge. **f** Photograph of a cubic SAC sponge sample obtained by stacking some square SAC sponge sheets. This shows how the thickness of the lamellar SAC sponge can be adjusted. Scale bar in **f**, 1 cm.

The fabrication process and design concept of the anisotropic lamellar SAC sponges are illustrated in Fig. 1a, b. Compared with previous methods for preparing ceramic aerogels^22,28,32^, our fabrication method is simple and scalable (Fig. 1c). The fabrication process of the SAC sponges mainly includes three procedures: preparation of a Poly(vinyl alcohol) (PVA)-TEOS-AlCl_3_ sol-gel solution, blow spinning of the sol-gel solution, and calcination of the as-spun PVA-SiO_2_–Al_2_O_3_ composite (PSAC) sponges (Supplementary Fig. 1).

In our work, PVA was used as a tackifier for TEOS and AlCl_3_ to promote the gelation process and also used as a template for forming the microfibers during the blow spinning process (Supplementary Fig. 2 and Supplementary Discussion). TEOS was hydrolyzed under the catalysis of H_3_PO_4_ in the spinning solution, and the interactions among PVA, TEOS, and AlCl_3_ were examined by the Fourier transform-infrared spectra and ^27^Al, ^29^Si nuclear magnetic resonance (NMR) spectra analysis (Supplementary Fig. 3, Supplementary Fig. 4 and Supplementary Discussion). We can obtain transparent and spinnable solutions quickly when adding some AlCl_3_ in the solutions because AlCl_3_ can also serve as an acidic catalyst to promote the hydrolysis of TEOS (Supplementary Fig. 5 and Supplementary Discussion). As shown in Supplementary Fig. 6, the spinning solutions with some AlCl_3_ become transparent in 15 minutes, whereas the SiO_2_ precursor solution without AlCl_3_ remains an emulsion state in 3 hours. Therefore, the addition of AlCl_3_ can greatly shorten the preparation time of spinning solutions.

The PSAC sponges exhibit lamellar structure (Supplementary Figs. 7 and 8), and microfibers in the PSAC sponges have an average diameter of 4.8 μm (Supplementary Fig. 9a). The SAC sponges were obtained by calcinating the as-spun PSAC sponges in air to remove residual organic components (Fig. 1d). After calcination, the lamellar structure of the sponges can be preserved and the diameter of microfibers in the SAC sponges will be significantly reduced to ~2.7 μm (Supplementary Fig. 9b). The prepared SAC sponges were examined using X-ray photoelectron spectroscopy (XPS) (Supplementary Fig. 10), and characteristic peaks corresponding to Al_2p_, Si_2p3_, and Si_2s_ can be assigned to a tetrahedral Al-doped silica structure. The peak at 120 eV can be attributed to Al_2s_ owing to the existence of alpha Al_2_O_3_.

We also fabricated SiO_2_ sponges without adding AlCl_3_ in the silica precursor solution (Supplementary Figs. 11–15). It is well known that the sponges prepared by direct spinning have a disordered structure, which usually provides poor compression resistance. Adding a certain amount of AlCl_3_ to the spinning solution produces sponges with anisotropic lamellar structure and numerous stacked microfiber layers (Fig. 1). The single microfiber layer can be peeled off from the SAC sponge block (Fig. 1e and Supplementary Movie 1), and the layer is found to be transparent (Supplementary Fig. 16). In addition, the SAC sponges can be cut into various shapes and stacked to a desired thickness by virtue of the layered structure (Fig. 1e, f). We also demonstrated that the SAC sponge after absorbing water shows the characteristics of “gel state”. As shown in Supplementary Fig. 17 a, b, when the sponge absorbs water, it becomes a gel. Rheological test results showed that the storage modulus is constant and larger than the loss modulus in the angular frequency range of 0.5–200 rad s^−1^ (Supplementary Fig. 17c).

### Morphology of the SAC sponges

**Fig. 2:**
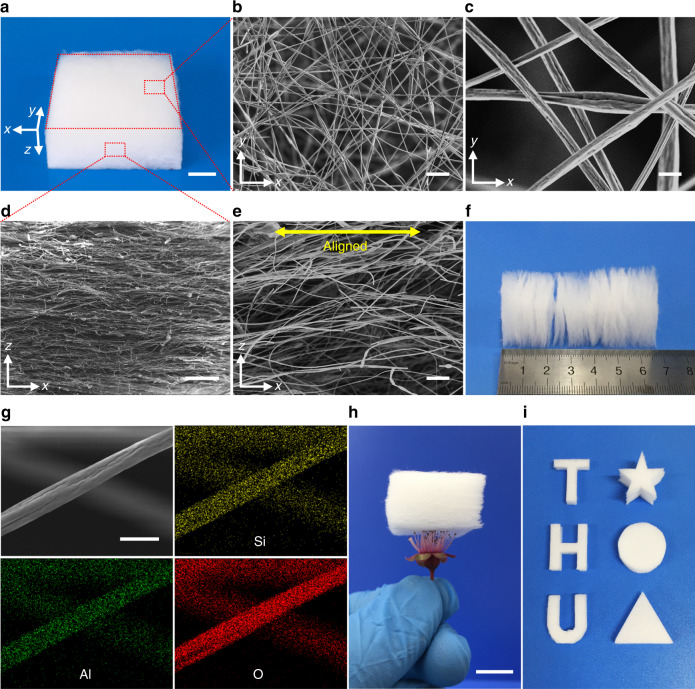
Microstructure of the SAC sponges. **a** Photograph of an SAC sponge. Scale bar in **a**, 1 cm. **b** Overhead SEM image of the SAC sponge. Scale bar in **b**, 50 μm. **c** Magnified overhead SEM image of the SAC sponge. Scale bar in **c**, 5 μm. **d** Cross-sectional SEM image of the SAC sponge. Scale bar in **d**, 500 μm. **e** Magnified cross-sectional SEM image of the SAC sponge. Scale bar in **e**, 50 μm. **f** Photograph showing the lamellar structure of the SAC sponges. **g** SEM image of a microfiber in the SAC sponge and corresponding elemental mapping images of Si, Al, and O. Scale bar in **g**, 5 μm. **h** Photograph showing an SAC sponge (*ρ* = 13 mg cm^−3^) placed on the stamen of an Armeniaca mume to show its lightweight. Scale bar in **h**, 1 cm. **i** Photograph of SAC sponges with various shapes.

Figure 2a shows a white SAC sponge block. The random fiber distribution can be visualized from an overhead view of the SAC sponge (Fig. 2b, c), and the lamellar structure and oriented fiber distribution can be seen in the cross-section of the SAC sponge (Fig. 2d, e). The layered structure of the SAC sponges can also be clearly observed in Fig. 2f. Energy dispersive spectrum and elemental mapping images show that the SAC sponges primarily contain O, Si, and Al (Fig. 2g and Supplementary Fig. 18). These elements are uniformly distributed throughout the microfibers. Our SAC sponge is lightweight, which was demonstrated by using the stamen of an Armeniaca mume to support it (Fig. 2h). The density of the SAC sponge can be as low as 10 mg cm^−3^, which is comparable to those of other inorganic aerogels^22,25^. The prepared SAC sponges can be cut into various desired shapes, such as a five-pointed star, circle, triangle, and even English letters, e.g., “T,” “H,” and “U” (Fig. 2i).

### Flexibility and compressibility of the SAC sponges

**Fig. 3:**
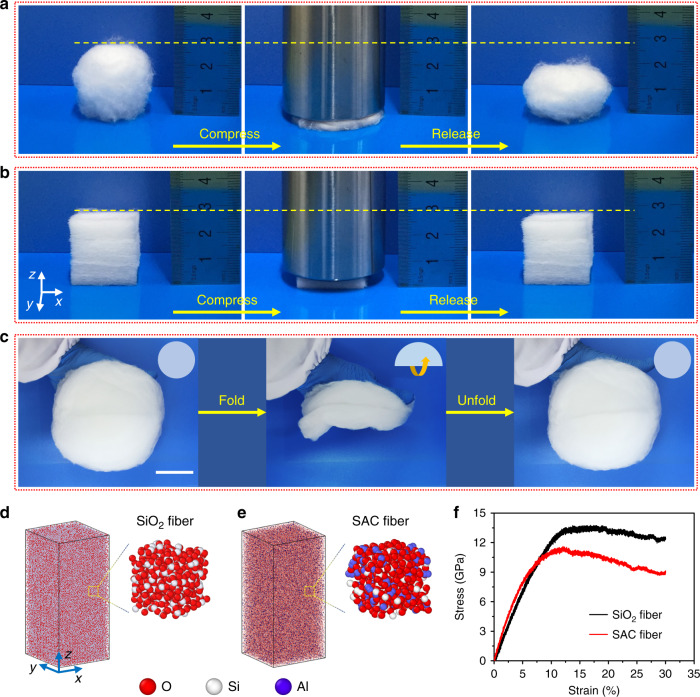
Flexibility and mechanical compressibility of the SiO_2_ and SAC sponges. **a** Photographs of an SiO_2_ sponge before compression, during compression, and after release. **b** Photographs of an SAC sponge before compression, during compression, and after release. **c** Photographs showing the flexibility of the SAC sponges. The schematics in the top right corner of the photographs are used to show the folding and unfolding of the samples. Scale bar in **c**, 5 cm. Atomistic models of **d** SiO_2_ fiber and **e** SAC fiber. **f** Stress–strain curves from the molecular dynamics simulations of the SiO_2_ fibers and SAC fibers under uniaxial tension.

We qualitatively studied the flexibility and mechanical compressibility of the SiO_2_ sponges and SAC sponges by compressing them using a 370 g metal block, as shown in Fig. 3. The two sponges can be compressed beyond 80% strain thanks to their high flexibility and porosity. However, the SiO_2_ sponge shows poor compressibility and the deformation cannot completely restore (Fig. 3a). Surprisingly, the SAC sponge completely recovers to its original shape without obvious dimensional change after pressure release (Fig. 3b and Supplementary Movie 2), illustrating its highly compressible characteristics. Note that the PSAC sponge did not show the same elastic recovery as SAC sponge, which may result from the softness of the PSAC microfibers (Supplementary Fig. 19).

The anisotropic structure of SAC sponges is also responsible for their anisotropic compressibility. In order to verify the link between structure and anisotropic compressibility, we further compressed the SAC sponges along the *x* direction (parallel to the stacked layers). The SAC sponges can also be compressed with >80% strain, but the shape cannot fully recover (Supplementary Fig. 20). This phenomenon is considerably different from that observed when the SAC sponges are compressed along the *z* direction (perpendicular to the stacked layers) (Fig. 3b). The significantly different compression of the SAC sponges along two orthogonal directions shows that the SAC sponges have a unique anisotropic mechanical property, which results from the anisotropic lamellar structure; many other types of sponge-like materials with anisotropic structures also exhibit the similar behavior^3,34–36^. In addition, the SAC sponges exhibit excellent flexibility, which was demonstrated by folding and unfolding the SAC sponges (Fig. 3c, Supplementary Fig. 21, and Supplementary Movie 3).

The mechanical properties of the SiO_2_ fibers and SAC fibers was investigated by molecular dynamics (MD) simulation (see details in Supplementary Notes). The modeling structures of the SiO_2_ fibers and SAC fibers with a size of 10 × 10 × 20 nm were built and stretched along *z* axis (Fig. 3d, e). Figure 3f shows the tensile stress–strain curves of the two samples. From the results, the Young’s moduli (*E*) of the SiO_2_ fibers and SAC fibers are found to be 142.0 GPa and 168.6 GPa, respectively. The tensile strength of the SiO_2_ fibers is 13.8 GPa and the corresponding tensile strain is up to 16.8%, demonstrating excellent flexibility. When the Al_2_O_3_ phase is added, the SAC fibers still possess a tensile strength of 11.6 GPa (corresponding to a tensile strain of 12.1%), which indicates that the SAC fibers also possess good ductility. In summary, the MD simulations suggest that the SAC fibers exhibit a higher Young’s modulus than that of SiO_2_ fibers, which can explain why the SAC sponges have better compression resilience. In addition, both SiO_2_ fibers and SAC fibers exhibit comparable ductility and flexibility.

### Compression fatigue resistance of the SAC sponges

**Fig. 4:**
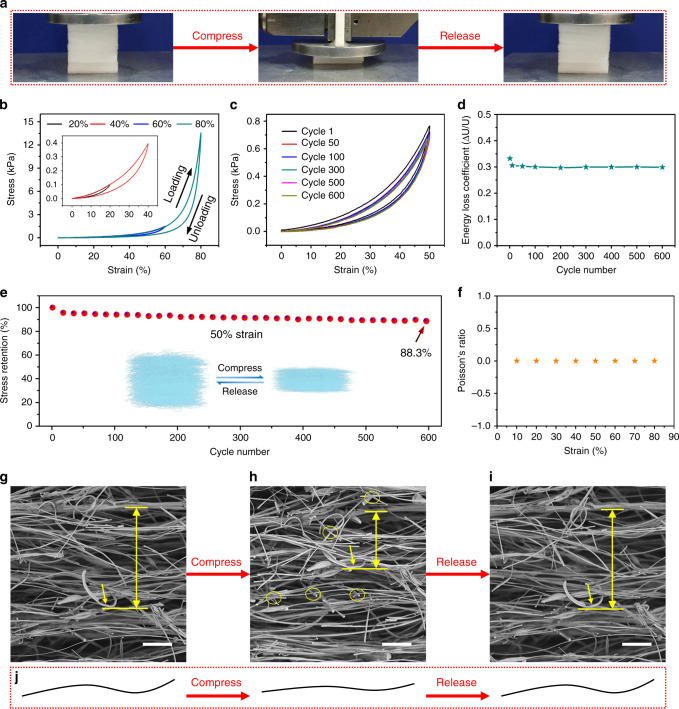
Mechanical properties of the SAC sponges under compression. **a** Photographs of the multi-cycle compression test. **b** Compressive stress–strain curves during loading–unloading cycles with different maximum strain. **c** Compressive stress–strain curves at 50% maximum strain and different numbers of cycles. **d** Energy loss coefficient △U/U of the SAC sponges at different numbers of cycles. **e** Maximum stress retention during 600 cycles at 50% maximum strain. **f** Poisson’s ratio of the SAC sponges versus strain. In situ SEM images of the SAC sponges **g** before compression, **h** during compression, and **i** after release. The ellipses in **h** show the lap joints between fibers. Scale bars in **g**–**i**, 50 μm. **j** Schematic showing the morphological changes of the microfibers in the SAC sponges during a compression cycle.

In order to further examine the compressibility of the SAC sponges, we quantitatively studied their behavior under compression using a universal testing machine (Supplementary Movie 4). Figure 4a shows photographs of the compression test. The stress–strain curve during loading exhibits three characteristic regions (Fig. 4b). There is a linear elastic deformation stage at strain <20%, indicating elastic bending of the SAC microfibers. A plateau stage emerges and lasts until the strain reaches 65%, which can be attributed to compression of space between microfiber layers. When the strain exceeds 65%, a nonlinear regime with sharply increased stress can be observed, which results from densification of the SAC sponges. The observed phenomena in the compressive stress–strain curves are similar to those reported for other sponge-like materials^3,28,29,37^. The maximum strain can reach 80% under an applied stress of 13.5 kPa, illustrating the high compressibility of the SAC sponges (Fig. 4b). In addition, SAC sponges with different densities exhibit different compressive mechanical strength (Supplementary Figs. 22 and 23). Note that the density of the original sponges is 10 mg cm^−3^. We regulated the density by compressing the sponges to certain thickness, and sponges with different densities were obtained.

Previous studies suggest a scaling law of *E*/*E*_s_ ~ (*ρ*/*ρ*_s_)^3^ (where the properties of bulk constitutive solids are denoted by subscript “s”) for cellular materials consisting of weakly interacting ligaments^38^. Herein, we have *ρ*_s_ = 2.6 g cm^−3^ for the SiO_2_-Al_2_O_3_ ceramic and *ρ* in the range of 10–30 mg cm^−^^3^ for the SAC sponges. Inputting *E*_s_ = 168.6 GPa for SAC fibers obtained from MD simulations, the scaling law suggests that our sponges should have an elastic modulus of ~8 kPa to 130 kPa, which is comparable to the tangential moduli of our sponges from the stress–strain curves in Fig. 4b.

We performed multi-cycle compression tests to evaluate the compressibility and compression fatigue resistance of the SAC sponges (Fig. 4c). The SAC sponges can endure 600 loading–unloading fatigue cycles at a maximum strain of 50% with 100 mm min^−1^ loading rate. The SAC sponges recover elastically very quickly, despite the high loading–unloading rate. In addition, the stress–strain curves did not change obviously after 600 cycles, showing that the SAC sponges have excellent compression fatigue resistance and they do not show obvious plastic deformation. The energy loss coefficient △U/U can be determined from hysteresis loops between the loading and unloading curves, and the calculated △U/U value is shown in Fig. 4d. The energy loss coefficient decreases from 0.33 for the first cycle to 0.31 for the tenth cycle, and then remains near 0.30 for subsequent cycles. The energy loss coefficient of our SAC sponges is smaller than or comparable to that reported for other ceramic aerogels^22,28^, showing the potential of this material for energy damping. In addition, the SAC sponges retain 88.3% of their initial maximum stress after 600 compression cycles (Fig. 4e), demonstrating their high compression resistance and robust structure. We also found that the SAC sponges have a zero Poisson’s ratio during a loading–unloading cycle, and Poisson’s ratio is nearly independent of the compressive strain (Fig. 4f), which can be attributed to the layered structure of the SAC sponges.

The mechanical compressibility of the SAC sponges with a density of 15 mg cm^−3^ stored at room temperature for 2 months was studied to evaluate their mechanical stability after a long-term storage. The sponges can be compressed beyond 80% strain and almost completely return to their initial state without obvious dimensional change after pressure release (Supplementary Fig. 24). In addition, quantitative study also shows that there is no obvious deterioration in the mechanical properties of the SAC sponge after long-term storage at room temperature. The SAC sponges maintain 96.9% of the initial maximum stress after 100 compression cycles (Supplementary Fig. 25), indicating that our sponges can maintain good compression resilience after a long-term storage.

We captured SEM images to further clarify the mechanism governing the compressibility of the SAC sponges (Fig. 4g–j, Supplementary Fig. 26, and Supplementary Movie 5). The original SAC sponges contain many voids (Fig. 4g), thus they have low density. The microfibers in the SAC sponges become straighter and the microfiber layers become denser under compression (Fig. 4h). In addition, the fibers were not found to break during compression, demonstrating the robust structure of the SAC sponges. Many lap joints among fibers can be observed from the SAC sponges (Fig. 4h), and they play an important role on the compressibility of the sponges. When the stress releases, the SAC fibers repel each other at the lap joints, which combines with the high Young’s modulus of the fibers make them to fully return to their original shapes (Fig. 4i).

### High and low-temperature resistance of the SAC sponges

**Fig. 5:**
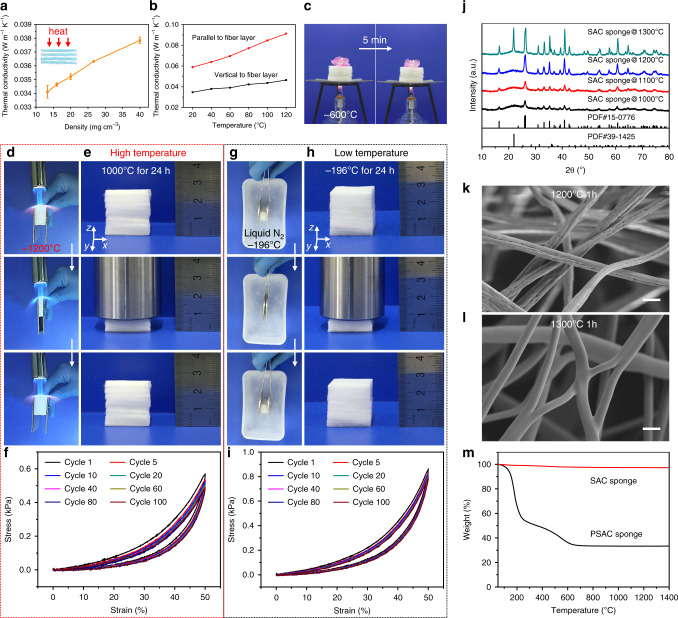
Thermal properties of the SAC sponges. **a** Thermal conductivity of SAC sponges with different densities. The inset is the schematic showing the heat flow direction during the thermal conductivity determination. Error bars represent s.d. (*n* = 3). **b** Thermal conductivity of the SAC sponges with a density of 16 mg cm^−3^ along different fiber layer directions at different temperatures. **c** Thermal insulation performance of the SAC sponges at high temperature; this figure shows how an SAC sponge prevents a fresh flower from withering when heated with an alcohol lamp. **d** Photographs showing the compressibility and fire resistance of the SAC sponges burned with a butane blowlamp. **e** Photographs showing the high compressibility of the SAC sponges treated at 1000 °C for 24 h. **f** Compressive stress–strain curves of the SAC sponges treated at 1000 °C for 24 h at 50% maximum strain for different numbers of cycles. **g** Photographs showing the compressibility of the SAC sponges immersed in liquid N_2_. **h** Photographs showing the high compressibility of the SAC sponges kept in liquid N_2_ for 24 h. **i** Compressive stress–strain curves of the SAC sponge kept in liquid N_2_ for 24 h at 50% maximum strain for different numbers of cycles. **j** XRD patterns of the SAC sponges calcinated at 1000, 1100, 1200, and 1300 °C for 1 h. SEM images of the SAC sponges obtained by calcination at **k** 1200 °C and **l** 1300 °C for 1 h. Scale bars in **k** and **l**, 5 μm. **m** Thermogravimetric analysis of the SAC sponges and PSAC sponges in air.

Sponge-like materials with low thermal conductivity are well-known thermal insulation materials and play an important role in reducing energy consumption^35,39–42^. The thermal conductivities of the SAC sponges were determined using a Hot Disk thermal conductivity analyser with the transient plane source method according to the testing standard of ISO 22007-2:2015 (Supplementary Fig. 27). The thermal conductivity vertical to the fiber layer direction for the SAC sponges with 13 mg cm^−3^ density is 0.034 W m^−1^ K^−1^ at 20 °C (Fig. 5a), which is comparable to other thermal insulating materials^43,44^. The low thermal conductivity of the SAC sponges can be ascribed to their low density and lamellar structure, where a large amount of air is retained within and between microfiber layers. When the density of the SAC sponges increased to 40 mg cm^−3^, the thermal conductivity slightly increased to 0.038 W m^−1^ K^−1^ at 20 °C owing to the reduced space between layers.

We also demonstrated the anisotropic thermal property of the SAC sponges by determining the thermal conductivity of the SAC sponges with a density of 16 mg cm^−3^ along different fiber layer directions. The thermal conductivity along different directions was found to increase as the temperature increased from 20 to 120 °C (Fig. 5b), which can be attributed to the increased thermal radiation from trapped air at higher temperature^45^. The thermal conductivity in the direction vertical to the fiber layer slowly increases from 0.035 W m^−1^ K^−1^ to 0.046 W m^−1^ K^−1^ when the temperature increases from 20 °C to 120 °C. Compared with the thermal conductivity vertical to the fiber layer direction, the SAC sponges show higher thermal conductivity in the direction parallel to the fiber layer. We ascribe the anisotropic thermal property of the SAC sponges to the anisotropic lamellar structure, and the air within and between fiber layers can effectively inhibit the heat transport. In order to demonstrate the thermal insulation performance of the SAC sponges, we put a fresh flower on an SAC sponge with 30 mm thickness and heated it using an alcohol lamp. After 5 minutes of heating, most petals on the flower remained fresh, indicating that our SAC sponges are good thermal insulators (Fig. 5c).

The combination of SiO_2_ and Al_2_O_3_ endows the SAC sponges with excellent fire resistance and high compressibility at high temperature, which was demonstrated using in situ compression tests while heated with an alcohol lamp to ~600 °C and with a butane blowlamp to ~1200 °C (Fig. 5d, Supplementary Fig. 28, and Supplementary Movie 6). No obvious structural changes or elasticity decay were observed when the SAC sponges were exposed to high temperature, illustrating their superelasticity at high temperature. When the SAC sponges were treated at 1000 °C for 24 h and then compressed, the treated, compressed SAC sponges recovered their original shape, indicating that our SAC sponges can withstand high temperature and maintain their high compressibility for long periods (Fig. 5e and Supplementary Movie 7). A quantitative study of the compressive mechanical property of the SAC sponges treated at 1000 °C for 24 h also shows that there was no significant deterioration in the mechanical properties of the SAC sponges after long-term treatment at high temperature (Fig. 5f and Supplementary Fig. 29).

Apart from the high-temperature superelasticity, our SAC sponges also exhibit excellent compressibility at low temperature. The SAC sponges remained flexible and can be compressed with ~80% strain in liquid N_2_ (−196 °C) (Fig. 5g and Supplementary Movie 8). When the stress was released, the SAC sponges returned to their original shape and did not exhibit obvious fracture. We also kept the SAC sponges in liquid N_2_ for 24 h, and their compressibility and compression fatigue resistance were investigated (Fig. 5h and i, Supplementary Fig. 30, and Supplementary Movie 9). As shown in Fig. 5h, the SAC sponges treated at low temperature can completely return to their initial shape after releasing stress, illustrating their excellent compressibility. In addition, the SAC sponges maintain 91.4% of the initial maximum stress after 100 compression cycles (Fig. 5i and Supplementary Fig. 30), demonstrating their long-term low-temperature resistance and high compression fatigue resistance.

We also investigated the compressibility and structure of the SAC sponges prepared at different calcination temperatures. All SAC sponges can recover their original shapes after releasing stress, illustrating their excellent compressive elastic resilience (Supplementary Fig. 31). Heat-induced structural change in the SAC sponges was analyzed using X-ray diffraction (XRD) in order to assess their thermal stability (Fig. 5j). The XRD patterns of the SAC sponges treated at 1000, 1100, and 1200 °C exhibit a broad peak at ~22°, corresponding to amorphous SiO_2_. The sample calcinated at 1300 °C exhibits a sharp peak near the original position of the broad peak, demonstrating the crystalline β-quartz structure of SiO_2_^28^. These results are consistent with the corresponding SEM images (Fig. 5k, l, Supplementary Fig. 32). When the SAC sponges were treated at 1300 °C for 1 h, the microfibers fused and adhered together, which can be ascribed to excessive crystallite growth at high temperature (Fig. 5l).

Compared with the pure SiO_2_ sponges, the presence of Al_2_O_3_ can inhibit the crystallization of SiO_2_. The pure SiO_2_ microfibers begin to crystallize at 1200 °C (Supplementary Fig. 15), whereas the initial crystallization temperature of the SAC microfibers is enhanced to 1300 °C (Fig. 5j). Therefore, the addition of AlCl_3_ can increase the flexibility of the sponges obtained at higher calcination temperature. In addition, we did not observe obvious changes in mass when the SAC sponges were heated to 1400 °C in air using thermogravimetric analysis, which also illustrates the excellent thermal stability of the SAC sponges (Fig. 5m).

### Comparison with other ceramic sponge preparation methods

**Fig. 6:**
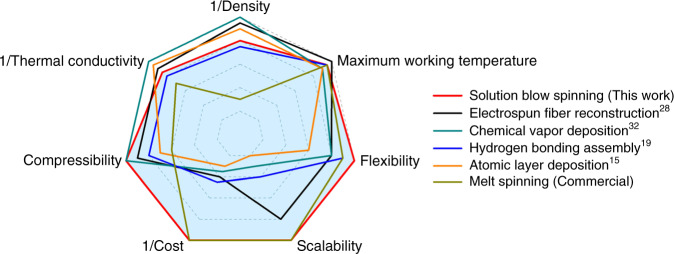
Comparison of preparation methods for ceramic sponges. A radar plot showing a comparison of the preparation methods and properties of ceramic sponges.

The SAC sponges exhibit excellent flexibility, temperature-invariant high compressibility, and low thermal conductivity. These properties are highly desirable as thermal insulation materials but it is very difficult to achieve all of them simultaneously. Compared with the most commonly used ceramic sponge preparation methods, including electrospun fiber reconstruction^28^, chemical vapor deposition^32^, hydrogen bonding assembly^19^, atomic layer deposition^15^, and melt spinning, our solution blow spinning method shows more advantages (Fig. 6 and Supplementary Table 1). Compared with electrospun fiber reconstruction and hydrogen bonding assembly, our preparation method does not need freeze drying procedure. Freeze drying is a time and energy-consuming process, which significantly increases the cost of ceramic sponges and severely limits their large-scale preparation and application. On the other hand, rigorous preparation conditions are required for chemical vapor deposition and atomic layer deposition, including inert gas atmosphere, vacuum, or high-temperature environment. In addition, these two deposition strategies require template preparation, and the template needs to be removed after deposition process, which leads to a low efficiency and high cost.

Melt spinning is a commonly used strategy for fabricating ceramic microfibers. Similar to electrospinning, melt spinning method also produces randomly distributed fibers and they show irregular shapes. In order to apply the melt spun fibers in real environments, they need to be processed into regular shapes. At present, ceramic fiber blankets have been fabricated using melt spinning combined with needling process. Note that the aluminosilicate needled blanket is a kind of typical ceramic fiber product. We compared the commercial ceramic fiber blanket with our sponge in terms of density, thermal conductivity, compressibility, flexibility, maximum working temperature, cost, and scalability (Fig. 6, Supplementary Table 1, Supplementary Fig. 33, and Supplementary Discussion). In comparison, our sponges have an ultralow density, relatively higher energy efficiency, and better compressibility than commercial ceramic fiber blanket. Taken together, our solution blow spinning method has obvious technical advantages in the preparation of ceramic sponges, and the fabricated SAC sponges show the unprecedented integration of high flexibility and compressibility, low thermal conductivity and low cost.

### Acoustic absorption property

Noise pollution from vehicles, machinery equipment, engineering construction has become a major health and environmental concern, and eliminating or reducing noise pollution is of great importance to human life. Sound absorption materials can be used to consume sound energy in the process of noise transmission, and some efficient sound absorption materials have been developed in recent years^46,47^. However, simultaneous achievement of excellent thermal insulation and acoustic absorption remains a huge challenge.

**Fig. 7:**
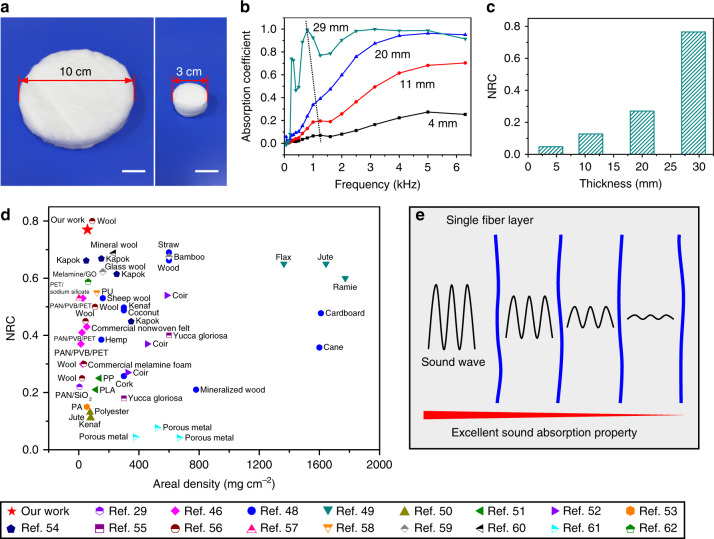
Acoustic absorption properties of the SAC sponges. **a** Optical images of the SAC sponges for the acoustic absorption property determination. Scale bars in **a**, 2 cm. **b** Sound absorption coefficient of the SAC sponges with different thicknesses. **c** Noise reduction coefficient (NRC) of the SAC sponges with different thicknesses. **d** Comparison of the sound absorption properties of our SAC sponges with other sound absorption materials. **e** Schematic showing the sound transmission though the SAC sponges.

In addition to good thermal insulation properties, we also demonstrated that our SAC sponges possess excellent sound absorption property. The sound absorption properties were measured using impedance tubes in the frequency range of 63–6300 Hz according to the test criteria of ISO 10534-2:1998 (Supplementary Fig. 34 and Supplementary Notes). The SAC sponges were cut into cylinders with diameters of 100 mm and 30 mm for the determination of sound absorption properties in the frequency ranges of 63–1600 Hz and 1000–6300 Hz, respectively (Fig. 7a). The absorption coefficient of the SAC sponges increases sharply with the increase of sound frequency, specifically for the SAC sponge with a thickness of 29 mm (Fig. 7b). The effect of thickness on the sound absorption properties was investigated, and the results are shown in Fig. 7b, c. The sound absorption coefficient of the SAC sponges increases with the increasing thickness, which can be explained by the increased sound propagation path.

The sound absorption properties of porous materials can be evaluated by noise reduction coefficient (NRC), which is the average of the sound absorption coefficients at 250, 500, 1000, and 2000 Hz. The NRC value significantly increases from 0.05 for the SAC sponge with a thickness of 4 mm to 0.77 for the SAC sponge with a thickness of 29 mm. We compared the sound absorption property of our SAC sponges with that of other sound absorption materials in terms of NRC and areal density (Fig. 7d and Supplementary Table 2)^29,46,48–62^. Our SAC sponges show lightweight characteristic and better sound absorption property. In addition, compared with commercial melamine foam and nonwoven felt with similar thickness, the SAC sponges also exhibit better sound absorption property^46^.

We ascribe the excellent sound absorption properties of our SAC sponges to the following reasons. First, the SAC sponges possess a lamellar structure, which leads to multiple absorption of sound waves between layers (Fig. 7e). Second, the microfibers in our SAC sponges have a rough surface (Fig. 2c), which can increase the friction between sound waves and our materials, thus causing more consumption of sound energy during the sound propagation progress. Third, a sound absorption peak can be observed at the frequency of ~1 kHz in the sound absorption curves (Fig. 7b), which can be attributed to the fiber vibration^63^. The resonant frequency of the SAC sponges at the absorption peak shows a tendency to move to a lower frequency with the increasing thickness, which can be ascribed to the increased areal density of the SAC sponges^64–66^. The fiber vibration caused by the sound waves may also play an important role in sound absorption.

Moisture resistance property is important for the practical application of sound absorption materials. Our SAC sponges exhibit a water contact angle of 128° (Supplementary Fig. 35a), which can prevent them from swelling and maintain the stable acoustic absorption properties. We placed our SAC sponges in ambient environment, and weighed them at different time to determine the weight retention rate. The weight of the samples remained stable at different times and no weight change can be observed (Supplementary Fig. 35b), indicating that our sponges do not absorb water in air. The moisture resistance property of our sponges was further demonstrated by moisture absorption and desorption test (Supplementary Fig. 35c, d, Supplementary Notes). The weight of the samples was increased by 51% after exposing them in a high-humidity atmosphere for 10 hours. The adsorbed water in the samples was desorbed quickly when keeping them in ambient environment with a relative humidity of 60% and the samples returned to their original weight in about 2 hours (Supplementary Fig. 35e), indicating that the SAC sponges possess good moisture resistance property. Water is mainly distributed on the surface of the fibers owing to their hydrophobic property, which results in the rapid moisture desorption.

## Discussion

Solution blow spinning is a fiber fabrication method that uses compressed air to draw threads of polymer solution to produce microfibers or nanofibers^67^. This method shares characteristics of both electrospinning and conventional melt spinning. The process does not require the use of high voltage or high temperature to form solid threads from solution, which makes this method can be applied to a wider range of material systems. When using solution blow spinning to deposit materials, with the aid of airflow, the microfibers or nanofibers can be stacked layer upon layer to form a three-dimensional structure in custom conformal geometries^68^.

Theoretically, anisotropic lamellar structural materials can be obtained from various spinnable polymer solution by blow spinning technique. However, our research suggests that the fiber diameter has an important effect on the structure of materials. For the SiO_2_ sponges without adding AlCl_3_ in the silica precursor solution, the deposited SiO_2_ fibers are easily blown away during the spinning process, which leads to a random fiber distribution. Note that the average diameter of the PVA-SiO_2_ composite microfibers is only 1.3 μm (Supplementary Fig. 13a). When adding some AlCl_3_ in the spinning solutions, the concentration of the solutions increases, which causes a larger fiber diameter. It can be observed that the diameter of the microfibers increases with the increasing AlCl_3_ content (Supplementary Fig. 36). The microfibers with significantly increased diameter can be confined in a specific area and cannot be blown away, thus anisotropic lamellar sponges are obtained.

The properties of materials are related to their structures. As shown in Supplementary Fig. 37, all the SAC sponges prepared from spinning solutions with different Al and Si molar ratio show good compression resilience. We attribute the excellent mechanical compression properties of the SAC sponges to their anisotropic lamellar structure. Most microfibers are distributed horizontally in the single fiber layer in the SAC sponges (Fig. 2d, e), and they show good compression resilience (Fig. 4g–j), which results in the excellent compression mechanical properties of the SAC sponges. For the SiO_2_ sponges, the fibers in the sponges exhibit disordered distribution (Supplementary Fig. 12c). When the fibers are compressed with a large strain, the fibers with a large inclination angle cannot fully return to their original state owing to the interference between the fibers. In addition, the SiO_2_ fibers have a smaller diameter (Supplementary Fig. 13b), and they are softer than SAC fibers, which is also responsible to the poor compressibility of the SiO_2_ sponges.

The lamellar structure can be easily obtained by the solution blow spinning technique. However, we have to admit that it is difficult to control the thickness of the single fiber layer. As can be seen in Fig. 2d–e, the single fiber layer cannot be easily distinguished in the bulk sponge. When peeled off from the sponge block, the single fiber layer cannot keep its original morphology very well and the thickness of the single fiber layer cannot be measured accurately (Supplementary Fig. 38). More work needs to be done to regulate the thickness of the single fiber layer and investigate the influence of the lamellar thickness on the properties of the SAC sponges.

For practical uses of ceramic fibers, the toxicity and health risk are the primary concerns for users. We performed toxicity test of our SAC sponges by implanting them in the subcutaneous tissue of C57 black 6 (C57BL/6 N) mice (see details in Supplementary Notes). The results of histological examinations show that no obvious necrosis can be observed after 3 weeks’ implantation, and the inflammation can be graded as minimal based on the number and distribution of inflammatory cells within the tissues surrounding the fibers (Supplementary Fig. 39), demonstrating good biocompatibility of the SAC sponges^69,70^.

In summary, we developed an anisotropic lamellar SAC sponge with temperature-invariant high compressibility using a facile sol-gel solution blow spinning technique. The anisotropic lamellar structure of the sponges with numerous stacked microfiber layers enabled the excellent mechanical compressibility up to 80% strain, high compression fatigue resistance of 600 cycles at 50% strain, robust mechanical stability with 88.3% initial maximum stress retention after 600 compression cycles, and strain-independent zero Poisson’s ratio. In addition, the layered structure and ceramic components also provide the SAC sponges with robust fire resistance, temperature-invariant compression resilience from −196 to 1000 °C, and excellent thermal insulation property with a thermal conductivity as low as 0.034 W m^−1^ K^−1^. The SAC sponges also demonstrated excellent sound absorption properties with an NRC value of 0.77, which is better than most reported sound absorption materials. Furthermore, the sponges could be cut into various shapes and stacked to a desired thickness by virtue of the layered structure. These outstanding mechanical and thermal properties make our SAC sponges a promising material for use as a fireproofing material, high-temperature thermal insulator, acoustic energy absorber, and catalyst support. Moreover, the successful preparation of anisotropic lamellar SAC sponges illustrates a method for developing flexible ceramic sponges via a simple, scalable solution blow spinning technique.

## Methods

### Materials and chemicals

Polyvinyl alcohol (Mowiol PVA-224, Mw~205,000) was purchased from Aladdin. Tetraethyl orthosilicate (TEOS > 99%) was provided by Shanghai Macklin Biochemical Co., Ltd. Phosphoric acid and aluminum chloride hexahydrate (AlCl_3_·6H_2_O) were purchased from Sinopharm Group Chemical Reagent Co., Ltd. Commercial ceramic fiber blanket was purchased from Yunxiang Sealing Insulation Material Factory.

### Preparation of the SAC sponges

The spinning solution was prepared using the following procedures. First, 1.125 g PVA powder was dissolved in 10.8 g deionized water by magnetically stirring at 90 °C for 1 h. 3.9 g TEOS, 0.0183 g phosphoric acid, and different masses of AlCl_3_·6H_2_O were subsequently added to the PVA solution. The mixed solutions were magnetically stirred at ambient temperature for ~ 2 h, yielding transparent solutions for preparing SAC sponges with Al and Si molar ratio of 0.1:1, 0.2:1, 0.4:1, and 0.8:1.

The spinning solution was loaded into a 1 mL syringe with a coaxial needle (the needle has an inner diameter of 0.21 mm and an outer diameter of 0.4 mm), and an injection pump was used to feed the solution to the spinneret at 5 mL h^−1^. The PSAC microfibers were obtained by blowing the solution using compressed air at ~50 kPa gas pressure. The PSAC sponges were collected using an air-permeable cage at ~50 cm from the nozzle. The blow spinning process was conducted at ambient temperature. Finally, the prepared PSAC sponges were calcinated in a muffle furnace at 1000–1300 °C in air for 1 h at 5 °C min^−1^ heating rate to remove residual organic components, yielding SAC sponges with high compressibility. We also prepared SiO_2_ sponges without adding AlCl_3_ to the spinning precursor solution. Unless otherwise specified, the SAC sponges used for characterization and determination were prepared from the spinning solutions with Al and Si molar ratio of 0.8:1.

### Characterization

Mechanical compression tests were conducted using a universal testing machine (Jinan assay testing machine Co., Ltd, China) with a 50 N load cell. The loading and unloading rate was set to 5 mm min^−1^ during stress–strain tests. The loading and unloading rate was 100 mm min^−1^ during cyclic compression fatigue tests. The storage modulus, loss modulus and damping ratio were determined using a rheometer (Physica MCR 302, Anton Paar, Austria) with a prestress of 0.5 N and a shear strain of 1% in the angular frequency range of 0.5–200 rad s^−1^. The microscopic morphologies of the sponges were examined using a field emission scanning electron microscope (FESEM, Zeiss, Germany). The elemental composition was examined with an energy disperse spectroscopy equipped on the FESEM. The distribution of microfiber diameter was obtained by analyzing 200 microfibers in the SEM images using Image-Pro Plus software (Media Cybernetics, USA). In situ observation of the SAC sponges was performed on a desktop scanning electron microscope (Phenom XL, Phenom Scientific, Netherlands). The thermal conductivity of the sponges was measured on a thermal conductivity analyser (TPS 2500 S, Hot Disk Instruments, Sweden) with the transient plane source method according to the testing standard of ISO 22007-2:2015. The SAC sponges were cut into squares with 50 mm side length for thermal conductivity determination. XRD patterns from the samples were recorded with an X-ray diffractometer (D/max-2500/PC, Rigaku, Japan) equipped with Cu Kα radiation with 2θ ranging from 10° to 80° at 10° min^−1^ scanning rate. The elemental composition of the SAC sponges was analyzed with an XPS (Escalab 250Xi, Thermo Fisher, USA) equipped with an Al Kα excitation source (1487.6 eV). The thermal stability of the samples was measured with a thermogravimetric-differential thermal analyzer (TG-DTA, Netzsch, Germany) at temperatures ranging from 50 to 1400 °C and 20 °C min^−1^ heating rate in air. The density of the SAC sponges was calculated using the measured mass and geometry of each sample. Water contact angles of the sponges were determined using a contact angle goniometer (JCY, Shanghai Fangrui Instrument Co., Ltd, China). Infrared (IR) spectra were recorded with a FTIR spectrometer (VERTEX 70 v, Bruker) at room temperature. The viscosity of the solutions was determined using a rheometer (Physica MCR 302, Anton Paar, Austria) in the shear rate range of 0.01–1000 s^−1^ at a constant temperature of 25 °C. ^27^Al and ^29^Si NMR measurement was performed on a 600 MHz NMR spectrometer (AVANCE NEO, Bruker, Germany) at room temperature.

### Reporting summary

Further information on research design is available in the Nature Research Reporting Summary linked to this article.

## Supplementary information

Supplementary Information

Peer Review File

Supplementary Movies

Reporting Summary

## Data Availability

The data that support the findings of this study are available from the corresponding author upon reasonable request.
