# Supplementary material for: Highly compressible and anisotropic lamellar ceramic sponges with superior thermal insulation and acoustic absorption performances
Source: Nat Commun. 2020 Jul 24;11:3732. doi: 10.1038/s41467-020-17533-6 (PMC7382455; doi:10.1038/s41467-020-17533-6)
Supplement: Supplementary file 1 — Supplementary Information [file 41467_2020_17533_MOESM1_ESM.pdf]

Supplementary Information

Highly compressible and anisotropic lamellar ceramic sponges with superior thermal insulation and acoustic absorption performances

Jia et al.

Supplementary Figures

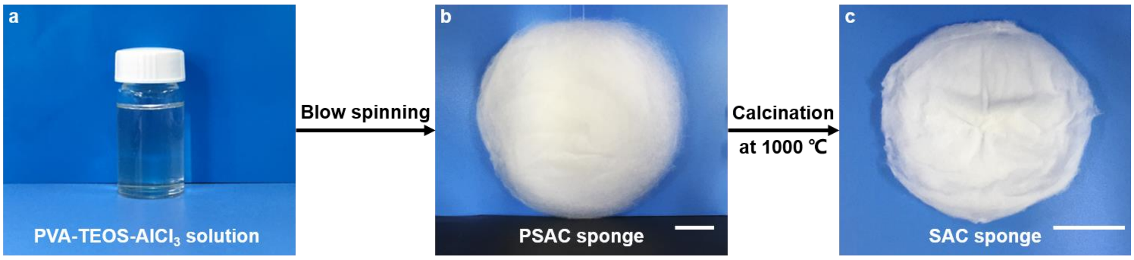

Supplementary Figure 1. Preparation process of the SiO_2-Al_2O_3 composite (SAC) sponges, which includes three procedures: preparation of a PVA-TEOS-$AlCl_3$ sol-gel solution, blow spinning of the sol-gel solution, and calcination of the as-spun PVA-SiO_2-Al_2O_3 composite (PSAC) sponges. **a** Optical image of the PVA-TEOS-$AlCl_3$ solution. **b** Optical image of the PSAC sponge. **c** Optical image of the SAC sponge. Scale bars in **b** and **c**, 5 cm.

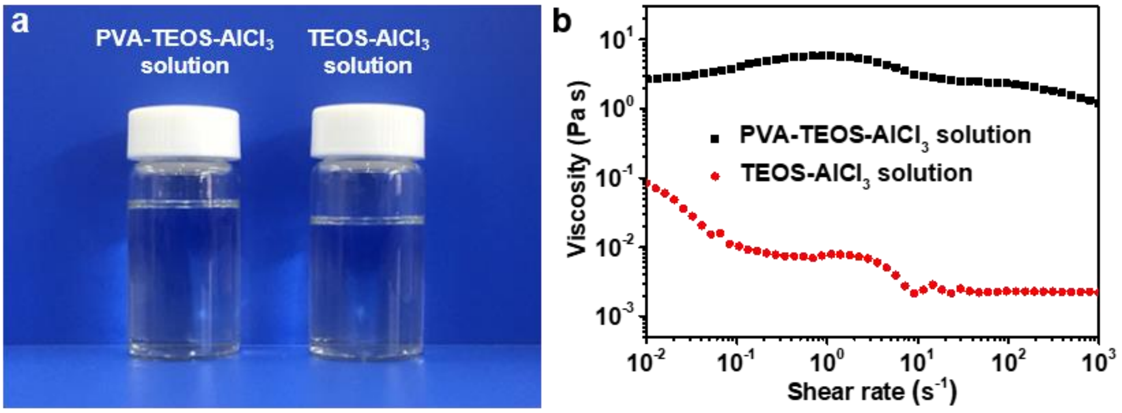

Supplementary Figure 2. a Optical image and **b** viscosity curves of the PVA-TEOS-$AlCl_3$ solution and TEOS-$AlCl_3$ solution.

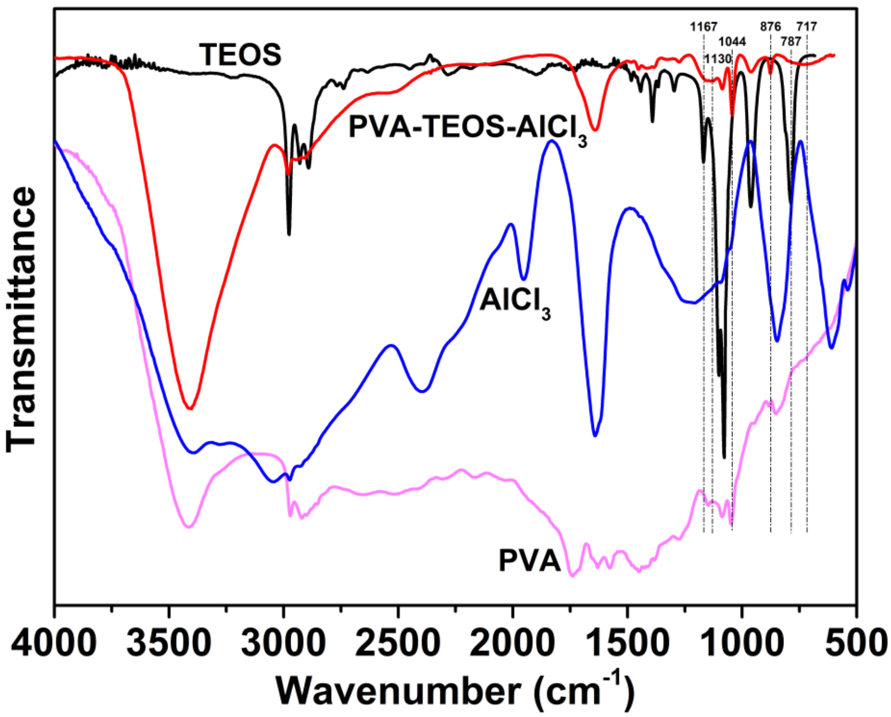

Supplementary Figure 3. FTIR spectra of PVA, TEOS, $AlCl_3$ and PVA-TEOS-$AlCl_3$ spinning solution.

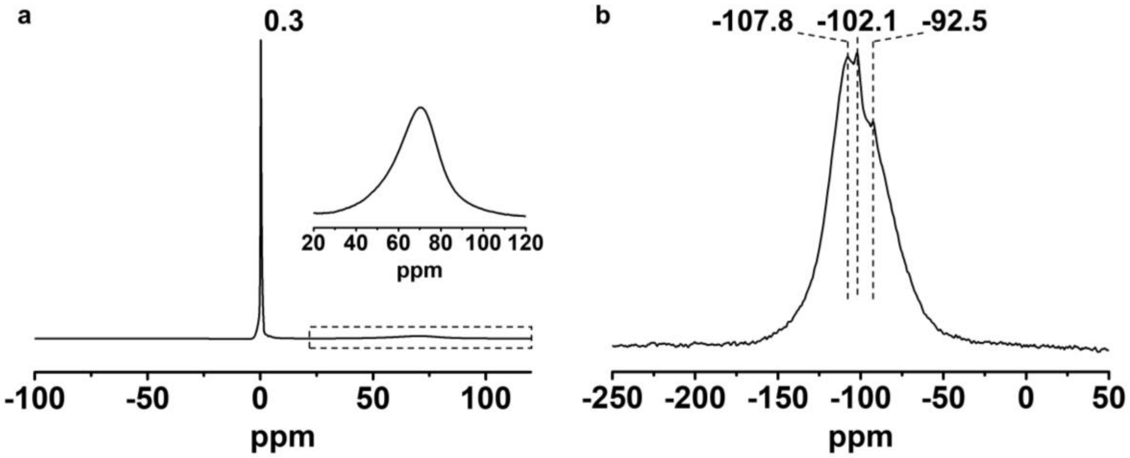

Supplementary Figure 4. a ^{27}Al and **b** ^{29}Si NMR spectra of the PVA-TEOS-$AlCl_3$ solution. The inset in **a** is the magnified NMR spectrum in the range of 20-120 ppm.

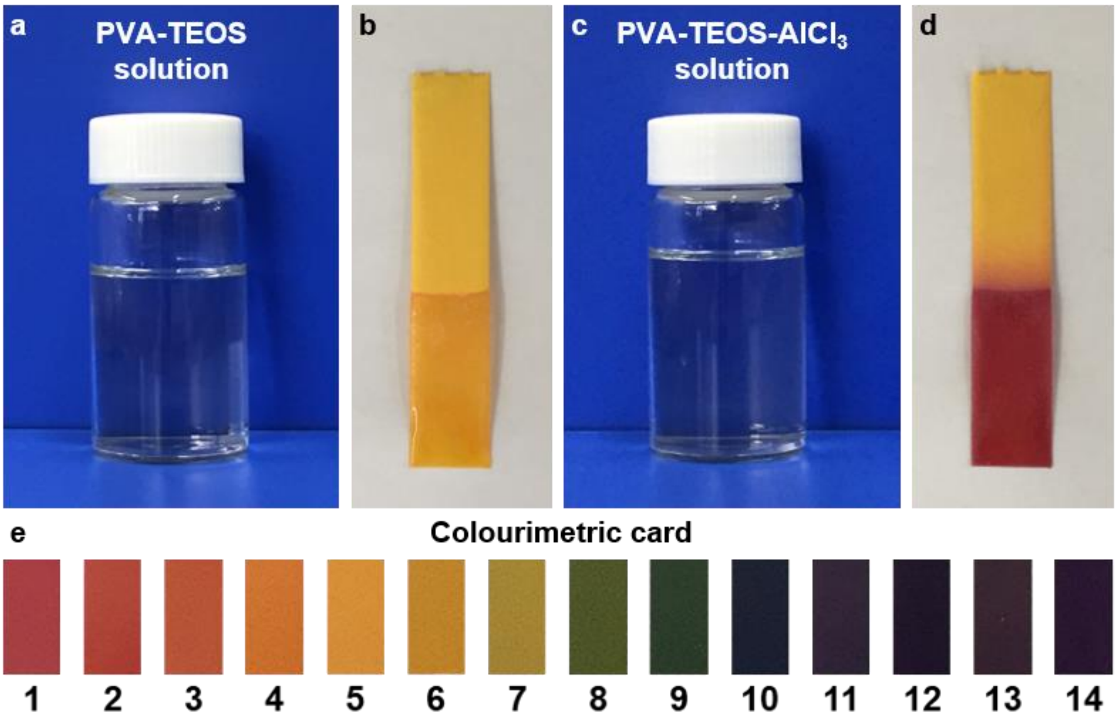

Supplementary Figure 5. pH determination of the PVA-TEOS solution and PVA-TEOS-$AlCl_3$ solution. **a, b** Optical images of the PVA-TEOS solution and pH indicator paper impregnated with the solution. **c, d** Optical images of the PVA-TEOS-$AlCl_3$ solution and pH indicator paper impregnated with the solution. **e** Optical image of the colourimetric card.

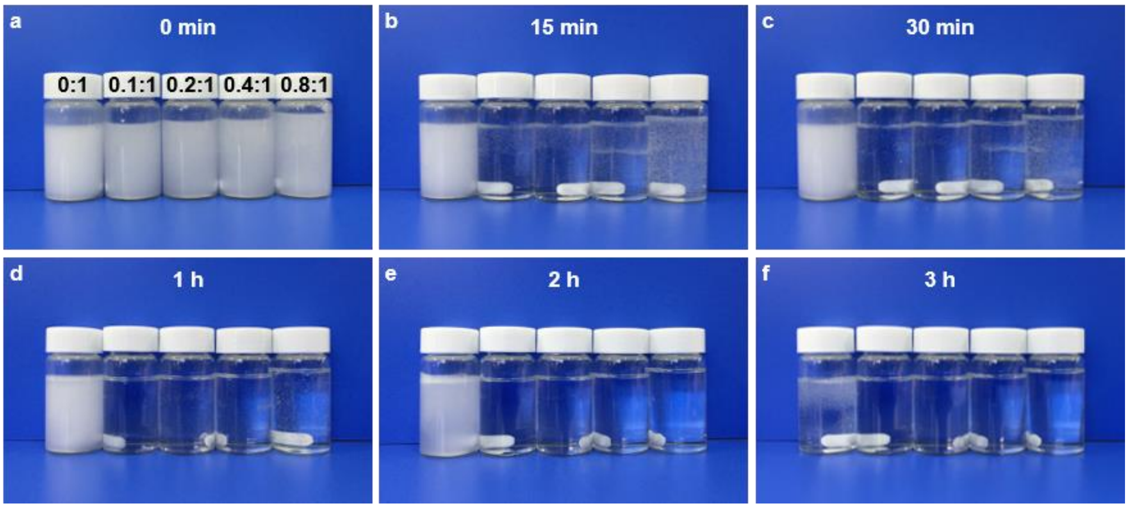

Supplementary Figure 6. Optical images of the spinning solutions with different molar ratio of Al and Si (From left to right: 0:1, 0.1:1, 0.2:1, 0.4:1 and 0.8:1) at **a** 0 min, **b** 15 min, **c** 30 min, **d** 1 h, **e** 2 h, **f** 3 h after all chemicals were added.

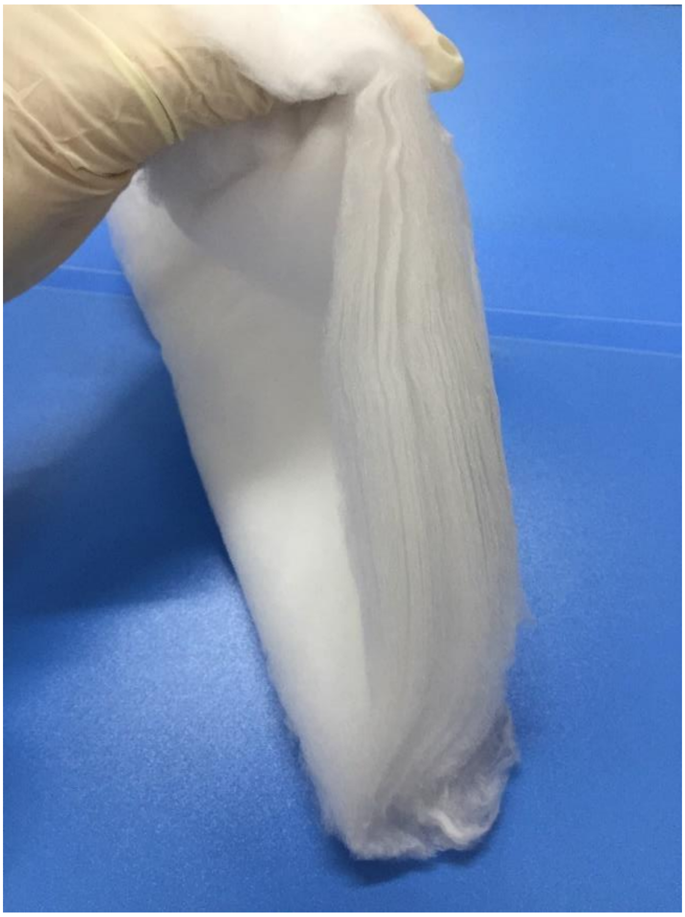

Supplementary Figure 7. Photograph showing the lamellar structure of the prepared PSAC sponge.

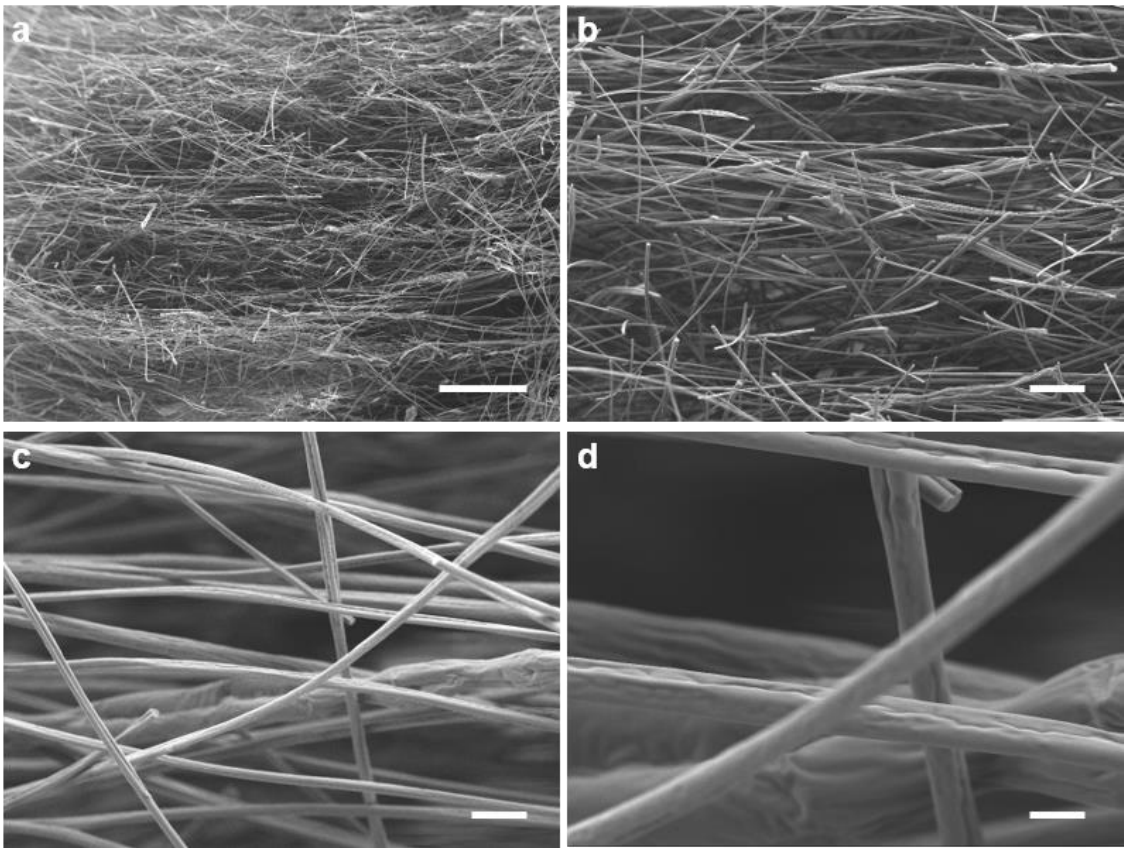

Supplementary Figure 8. Microstructure characterization of the PSAC sponges. **a** Cross-sectional SEM image of the PSAC sponge. Scale bar in **a**, 500 μm. **b-d** Magnified cross-sectional SEM images of the PSAC sponge. Scale bar in **b**, 100 μm. Scale bar in **c**, 20 μm. Scale bar in **d**, 5 μm.

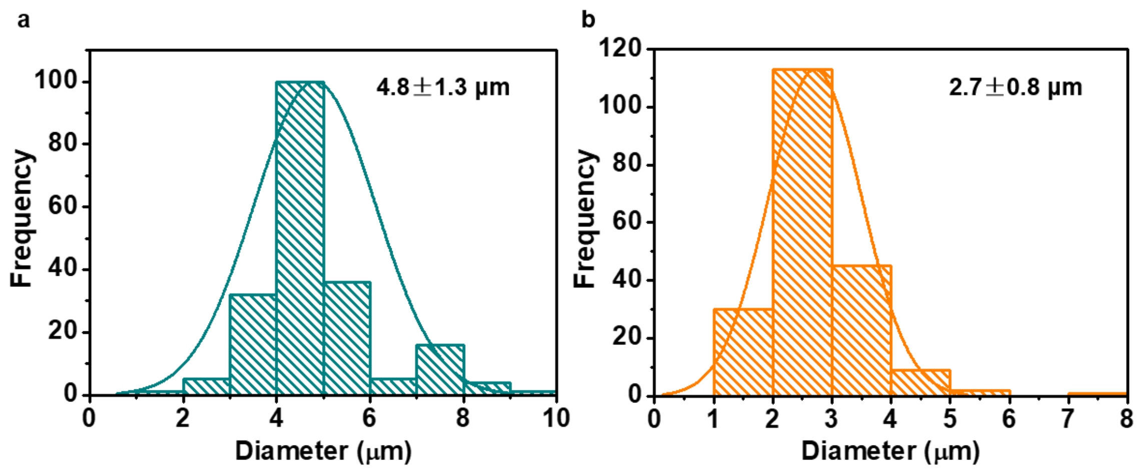

Supplementary Figure 9. Diameter distribution of the microfibers in the **a** PSAC sponges and **b** SAC sponges. The diameter of the microfibers significantly decreases after calcination.

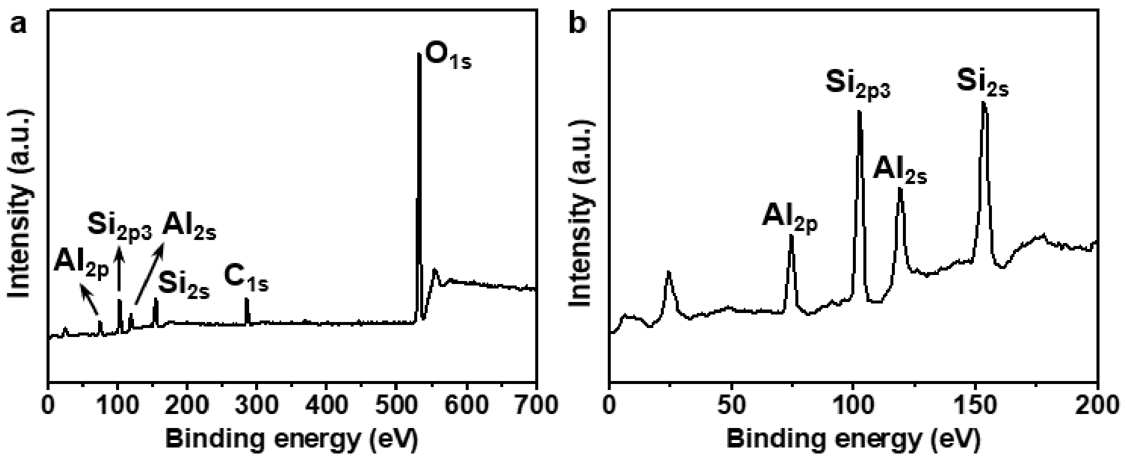

Supplementary Figure 10. XPS spectrum of the SAC sponges. **a** XPS spectrum of the SAC sponges for all elements. **b** Magnified XPS spectrum of the SAC sponges at binding energy ranging from 0 to 200 eV.

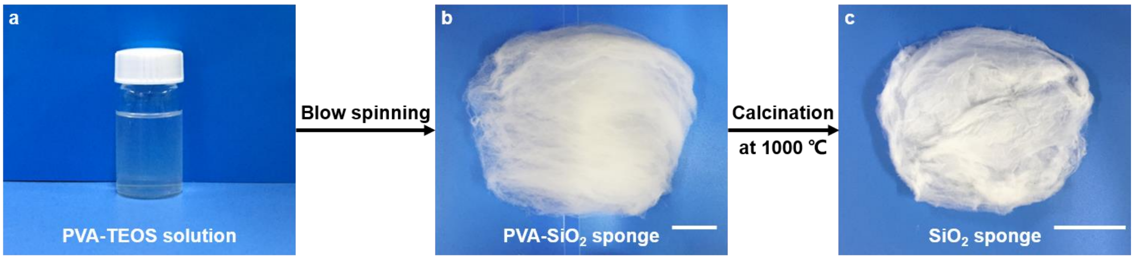

Supplementary Figure 11. Preparation process for the SiO_2 sponges, which includes three procedures: preparation of the PVA-TEOS sol-gel solution, blow spinning of the sol-gel solution, and calcination of the as-spun PVA-SiO_2 composite sponges. **a** Optical image of the PVA-TEOS solution. **b** Optical image of the PVA-SiO_2 sponge. **c** Optical image of the SiO_2 sponge. Scale bars in **b** and **c**, 5 cm.

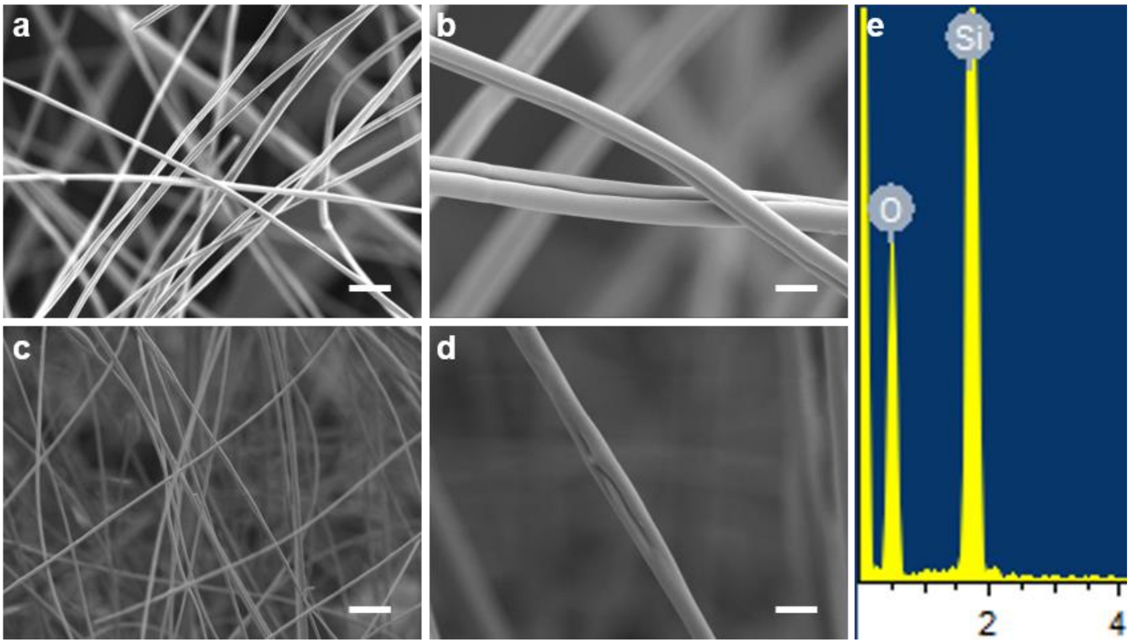

Supplementary Figure 12. Morphology characterization of the PVA-SiO_2 composite microfibers and SiO_2 microfibers. **a-b** SEM images of the PVA-SiO_2 composite microfibers. Scale bar in **a**, 10 μm. Scale bar in **b**, 2 μm. **c-d** SEM images of the SiO_2 microfibers. Scale bar in **c**, 10 μm. Scale bar in **d**, 2 μm. **e** SEM-EDS image of the SiO_2 microfibers. The SiO_2 sponges primarily consist of O and Si, indicating the successful fabrication of SiO_2 sponges.

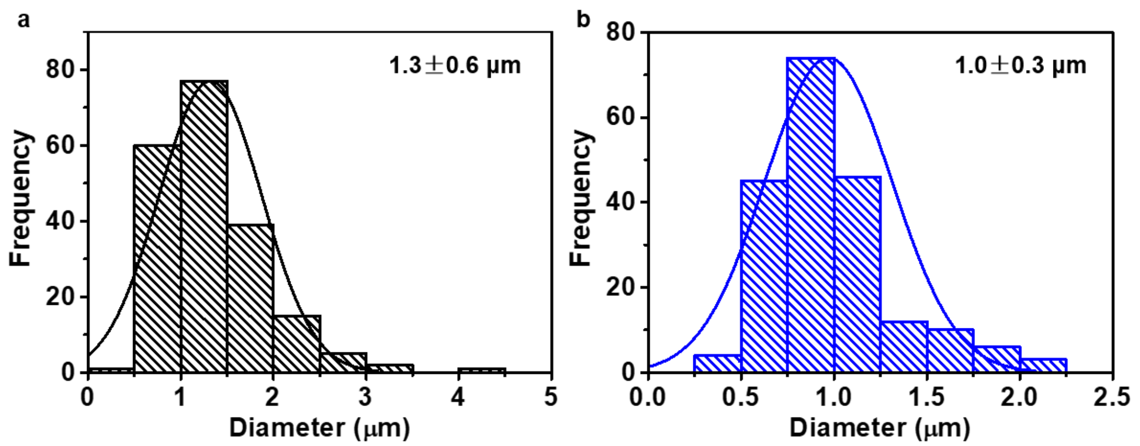

Supplementary Figure 13. Diameter distribution of **a** PVA-SiO_2 composite microfibers and **b** SiO_2 microfibers.

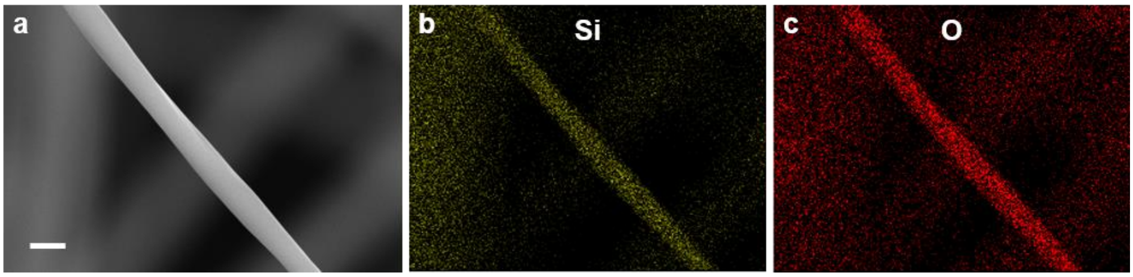

Supplementary Figure 14. a SEM image of an SiO_2 microfiber and the corresponding elemental mapping images of **b** Si and **c** O, respectively. Scale bar in **a**, 1 μm.

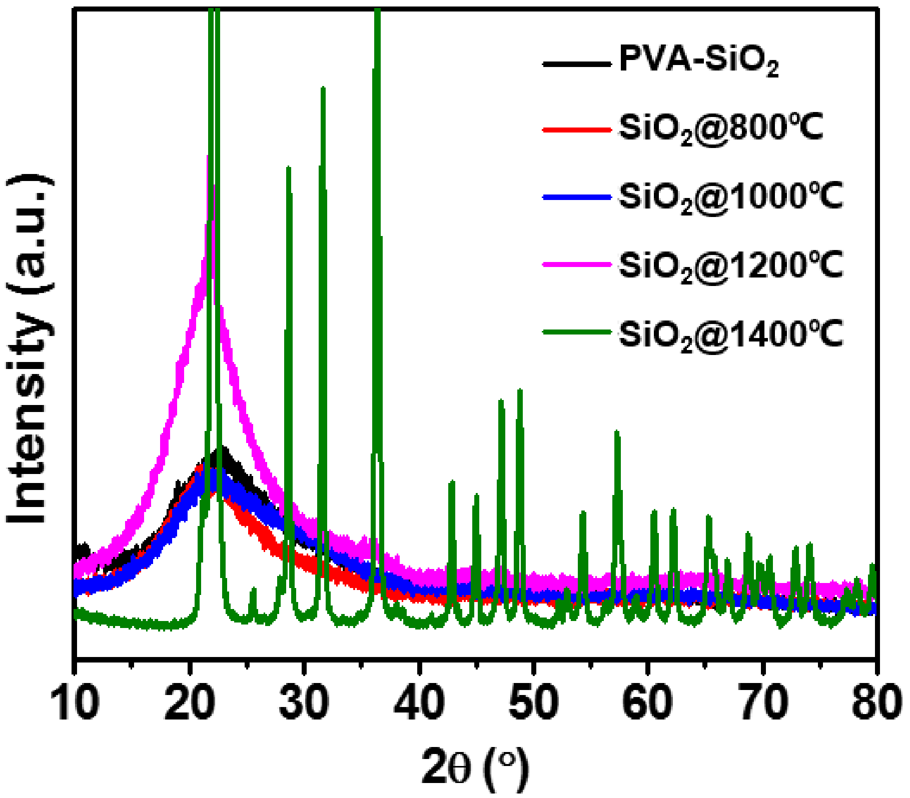

Supplementary Figure 15. XRD patterns of the PVA-SiO_2 composite microfibers and SiO_2 microfibers obtained at different calcination temperatures.

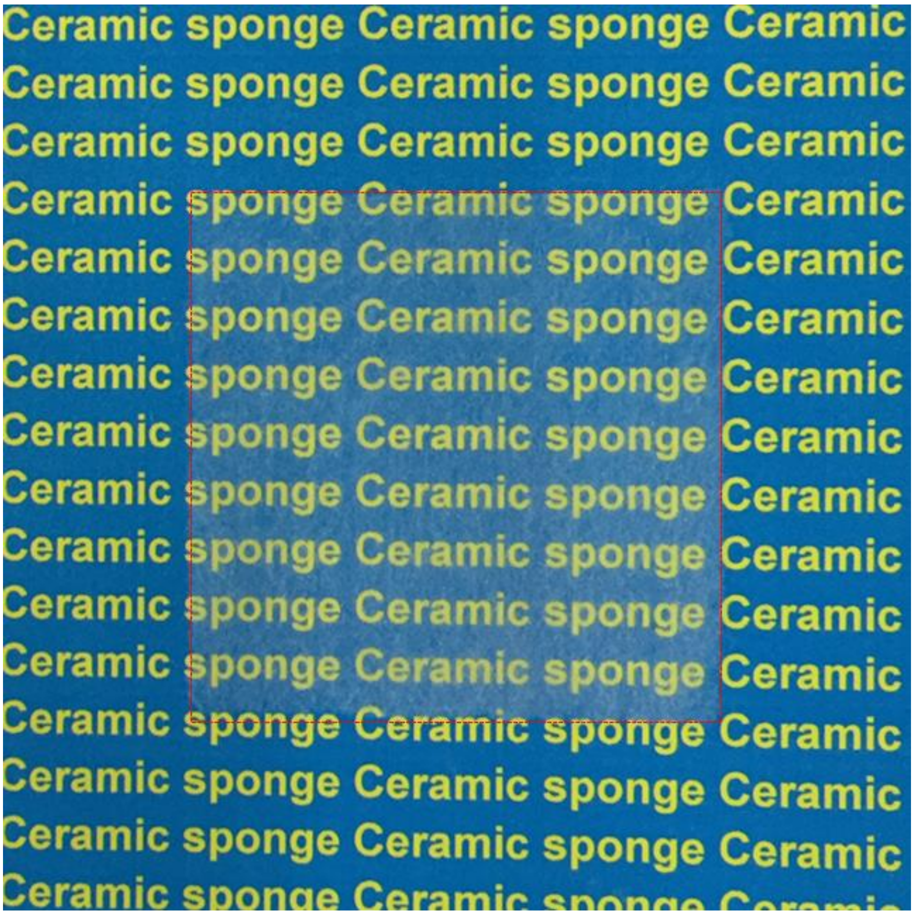

Supplementary Figure 16. Photograph of a single microfiber layer to show its transparency.

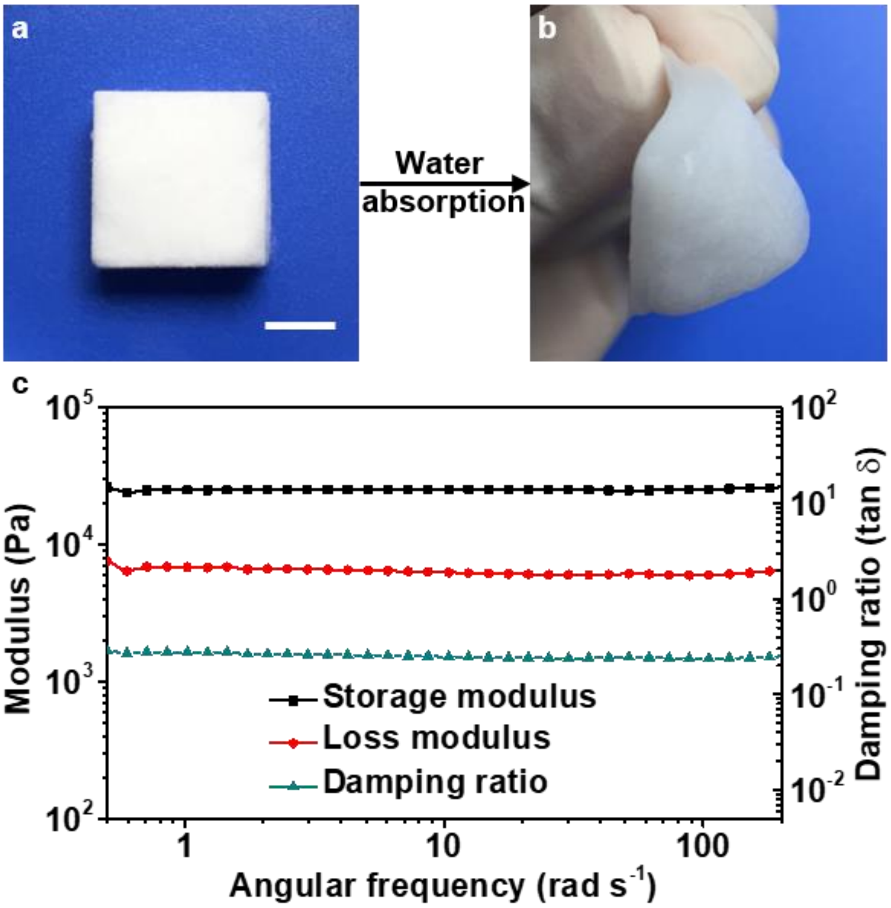

Supplementary Figure 17. Optical images of the SAC sponge **a** before and **b** after water absorption. Scale bar in **a**, 1 cm. **c** Frequency dependence of the storage modulus, loss modulus, and damping ratio for the wet sponge gel (shear strain of 1%) at room temperature.

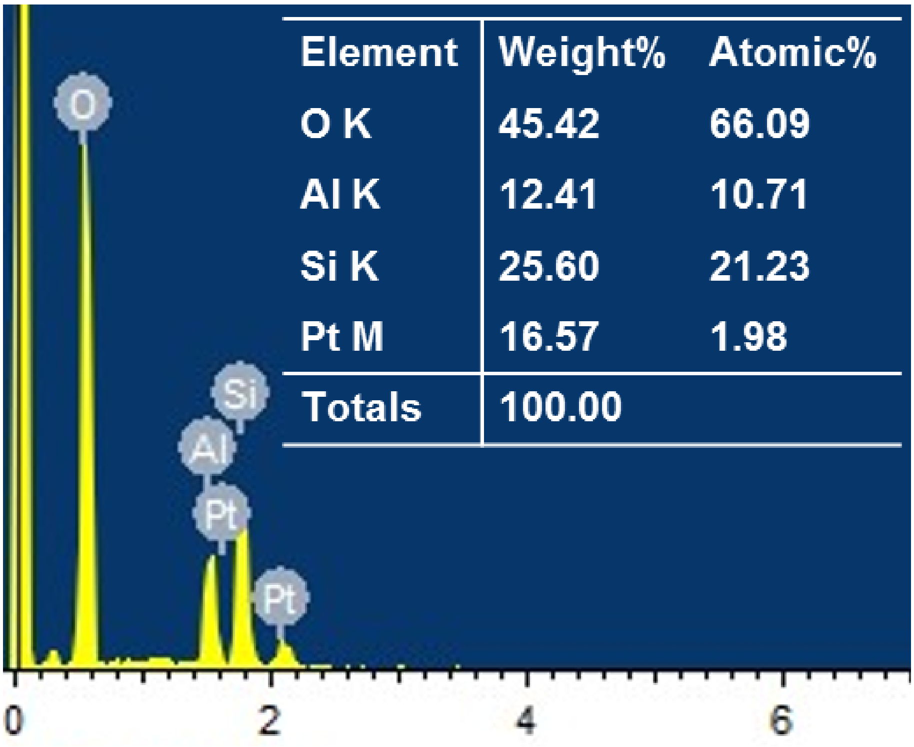

Element	Weight%	Atomic%
O K	45.42	66.09
Al K	12.41	10.71
Si K	25.60	21.23
Pt M	16.57	1.98
Totals	100.00	

Supplementary Figure 18. SEM-EDS image of the SAC sponges and the content of various elements. The SAC sponges primarily contain O, Si, and Al. A small amount of Pt can be seen, which was used to increase the electrical conductivity during SEM imaging.

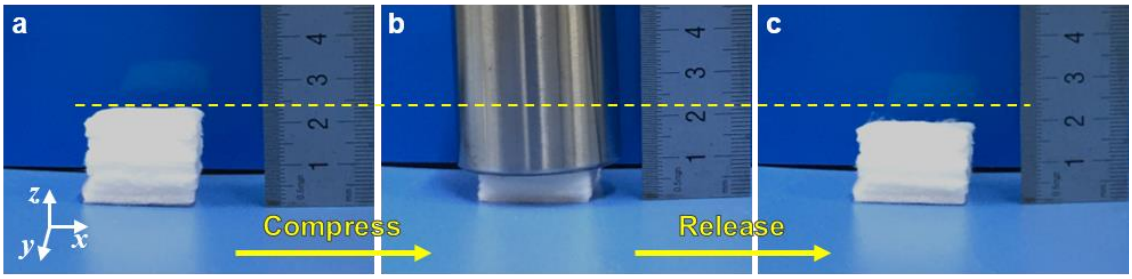

Supplementary Figure 19. Photographs of the PSAC sponge **a** before compression, **b** during compression, and **c** after release.

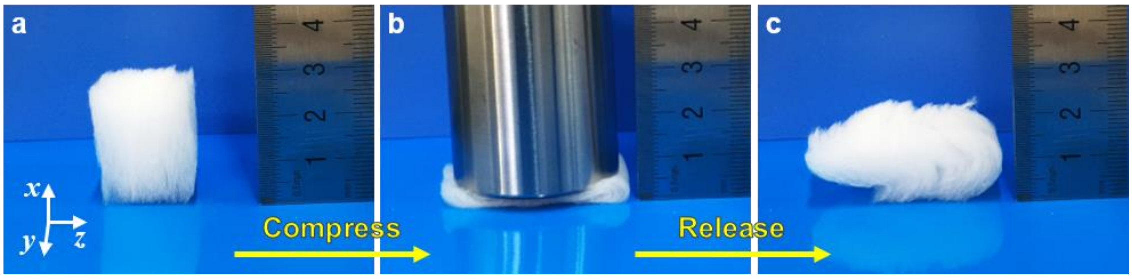

Supplementary Figure 20. Photographs of the SAC sponge compressed along the *x* direction **a** before compression, **b** during compression, and **c** after release.

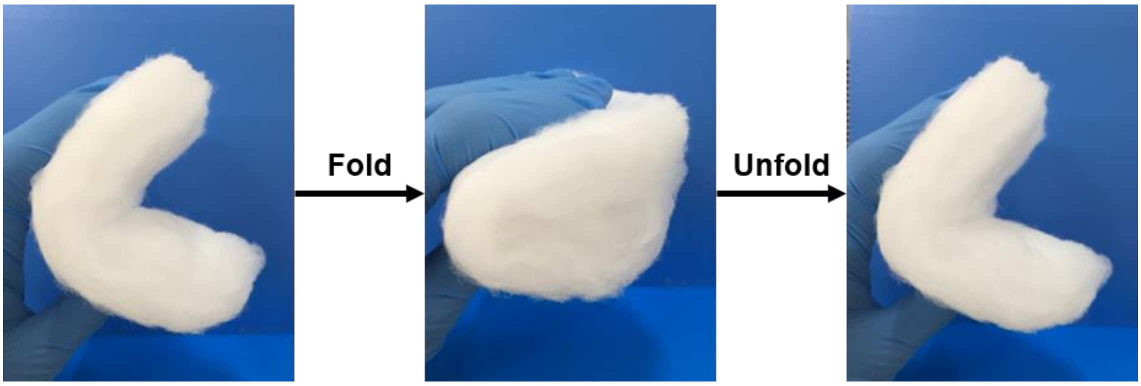

Supplementary Figure 21. Photographs showing the flexibility of the SAC sponges.

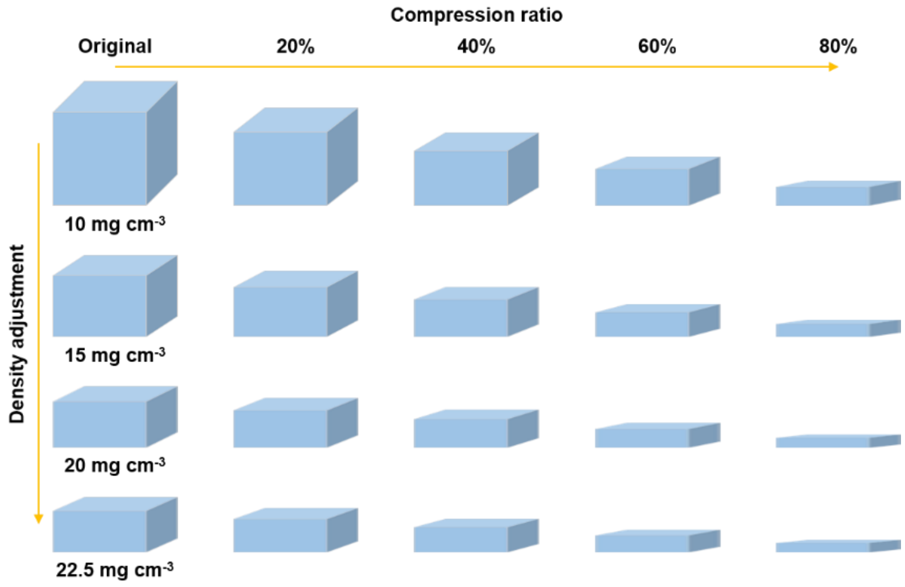

Supplementary Figure 22. Schematic showing the density adjustment of the SAC sponges and the sponges with different compression ratios.

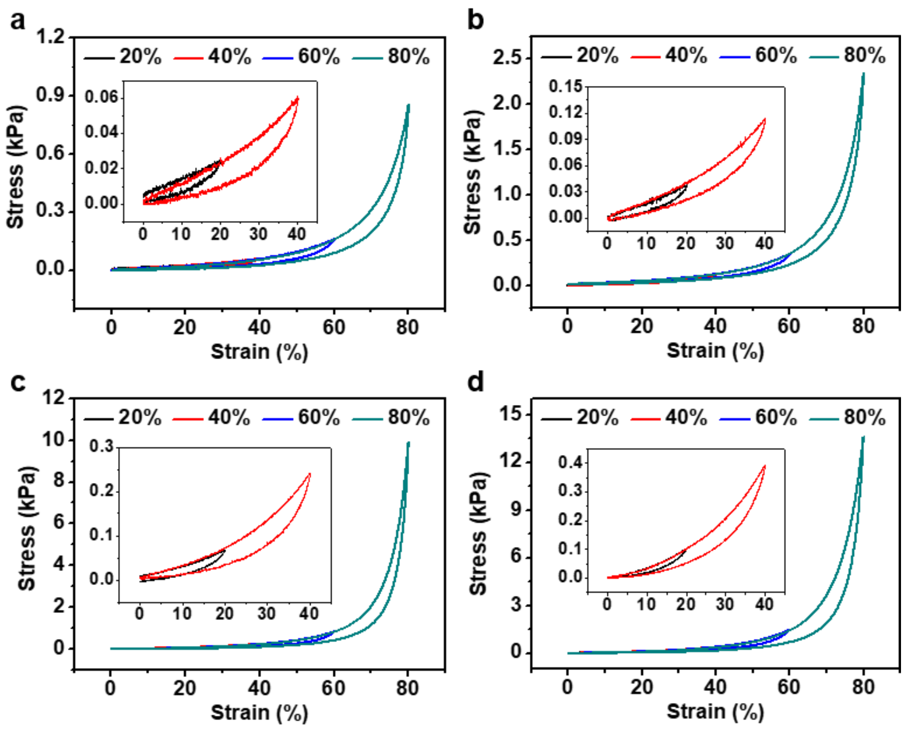

Supplementary Figure 23. Compressive stress-strain curves from the SAC sponges with different densities during loading and unloading cycles at different maximum strain: **a** 10 mg cm^{-3}, **b** 15 mg cm^{-3}, **c** 20 mg cm^{-3}, and **d** 22.5 mg cm^{-3}.

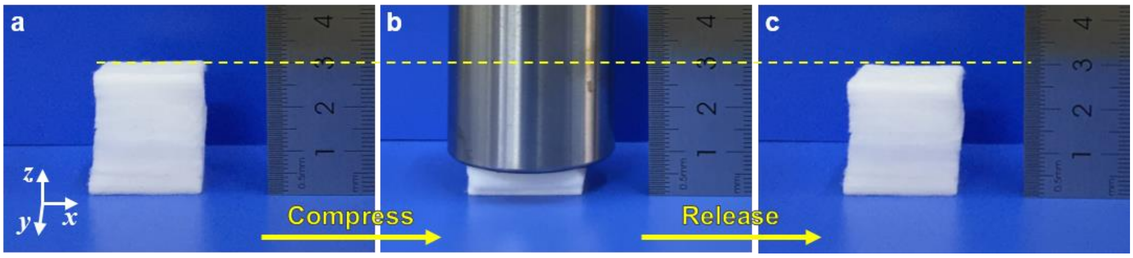

Supplementary Figure 24. Optical images of an SAC sponge stored at room temperature for 2 months, **a** before compression, **b** during compression, and **c** after release.

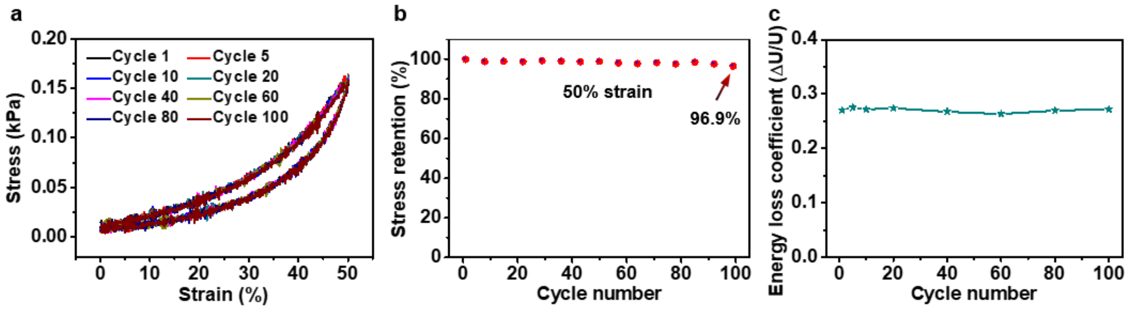

Supplementary Figure 25. Mechanical compressibility of the SAC sponge stored at room temperature for 2 months. **a** Compressive stress-strain curves of the SAC sponge (with a density of 15 mg cm^{-3}) after 2 months storage at 50% maximum strain for different numbers of cycles. **b** Maximum stress retention during 100 cycles at 50% maximum strain. **c** Energy loss coefficient △U/U of the SAC sponge at different numbers of cycles.

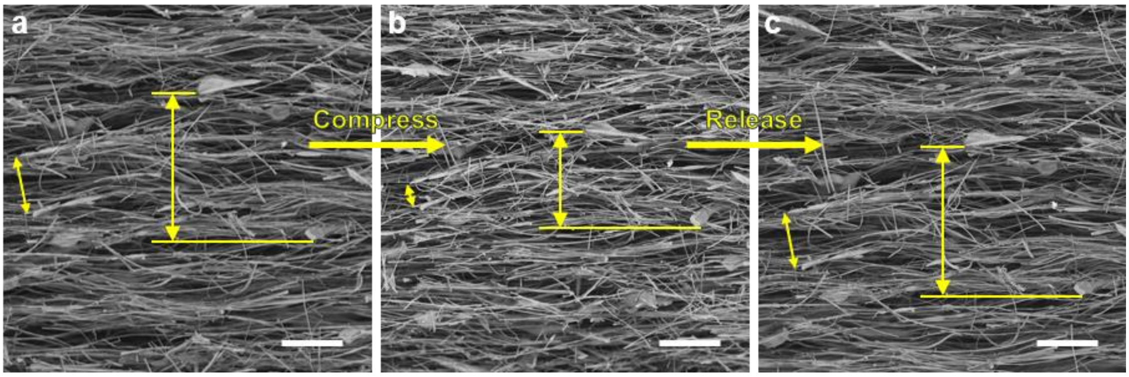

Supplementary Figure 26. *In-situ* SEM images of the SAC sponges **a** before compression, **b** during compression, and **c** after release. Scale bars in **a-c**, 150 μm.

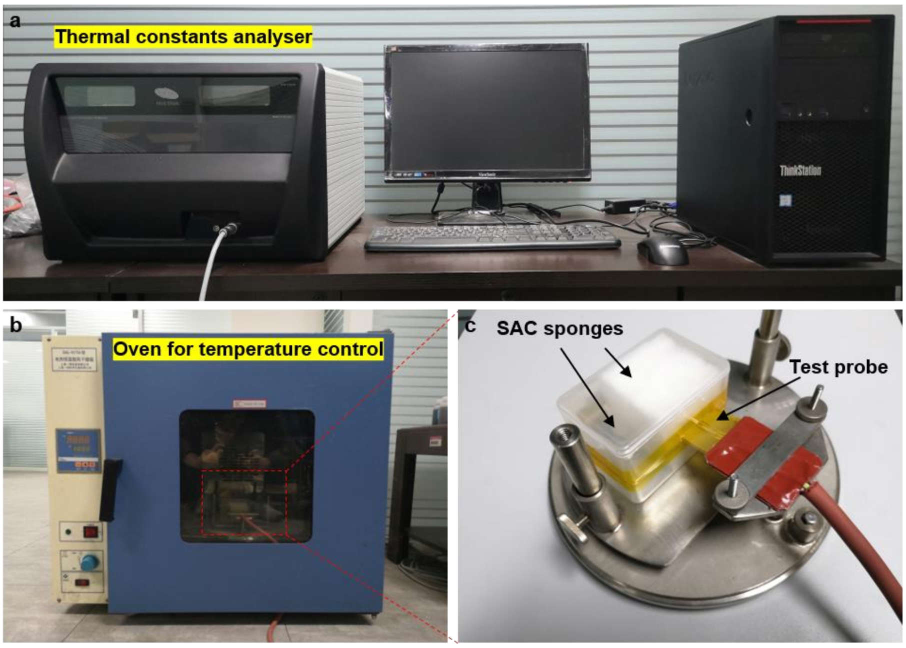

Supplementary Figure 27. Optical image of the thermal conductivity determination system. **a** Optical image of the thermal constants analyser, **b** optical image of the oven for temperature control, and **c** optical image of the test probe and SAC sponge samples.

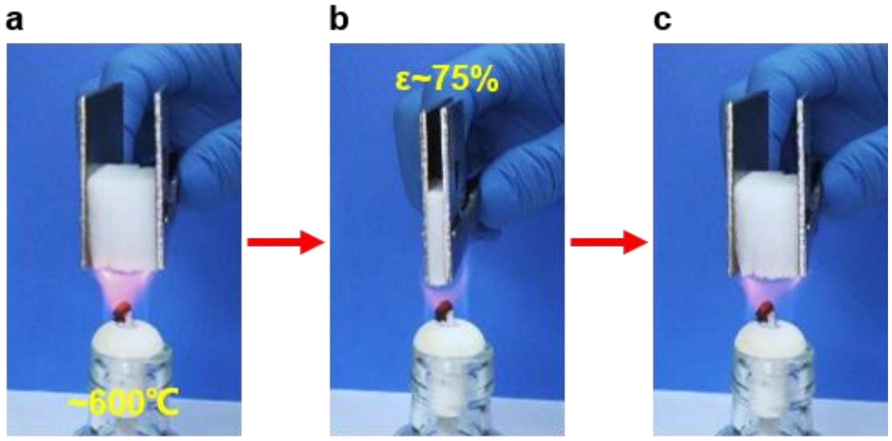

Supplementary Figure 28. Photographs showing compression recovery and fire resistance of the SAC sponges burned with an alcohol lamp. **a** Before compression, **b** during compression, and **c** after release.

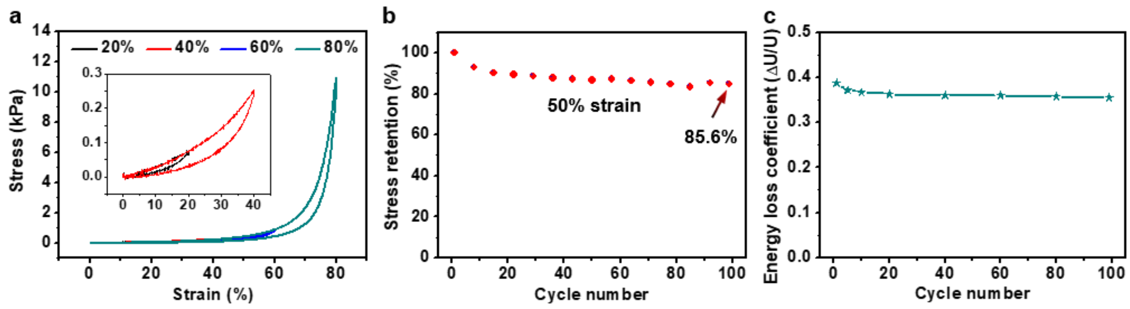

Supplementary Figure 29. a Compressive stress-strain curves of the SAC sponges treated at 1000°C for 24 h during loading and unloading cycles at different maximum strains. **b** Maximum stress retention during 100 cycles at 50% maximum strain. **c** Energy loss coefficient △U/U of the SAC sponges at different numbers of cycles.

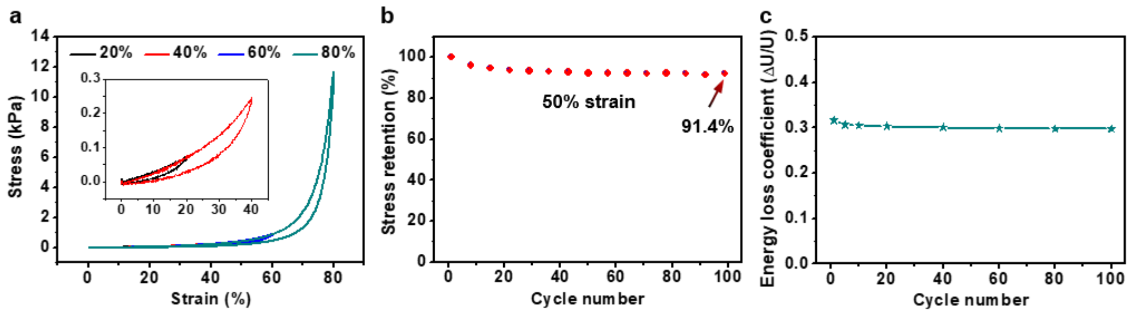

Supplementary Figure 30. a Compressive stress-strain curves of the SAC sponges treated at -196°C for 24 h during loading and unloading cycles with different maximum strain. **b** Maximum stress retention during 100 cycles at 50% maximum strain. **c** Energy loss coefficient △U/U of the SAC sponges for different numbers of cycles.

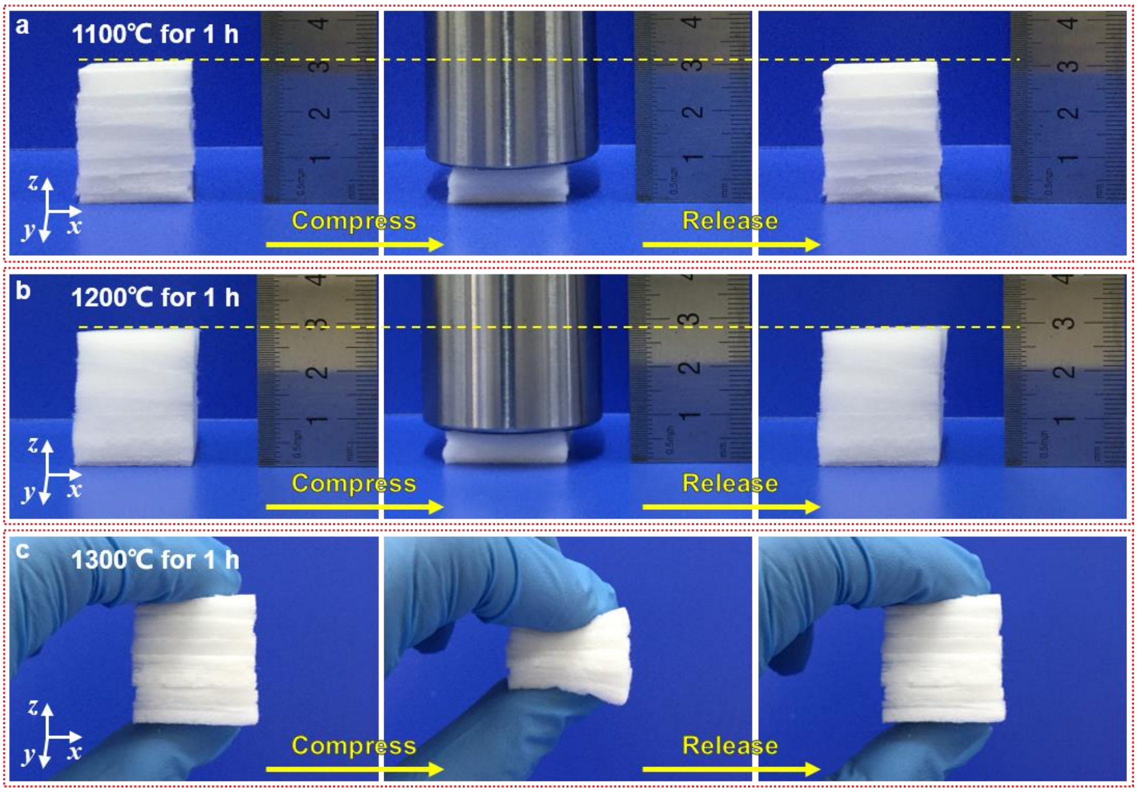

Supplementary Figure 31. Photographs of the SAC sponges prepared by calcination at **a** 1100, **b** 1200, and **c** 1300°C for 1 h before compression, during compression, and after release.

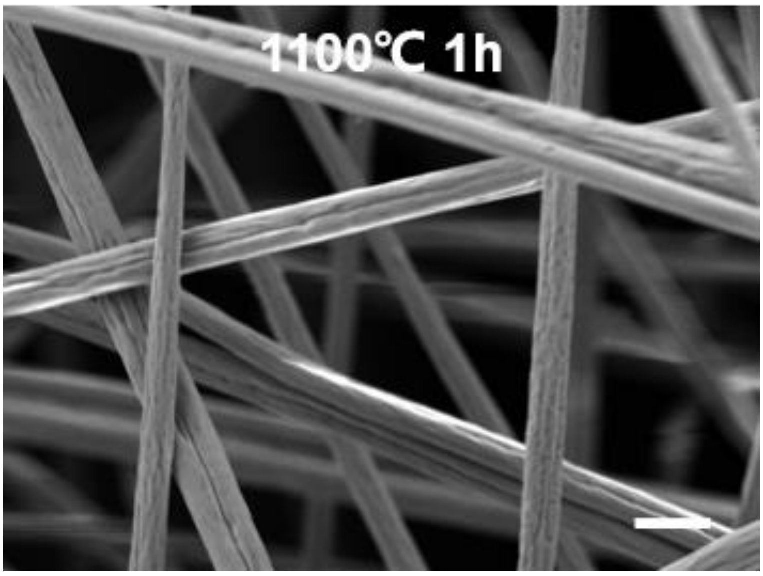

Supplementary Figure 32. SEM image of the SAC sponges obtained by calcination at 1100°C for 1 h. Scale bar, 5 μm.

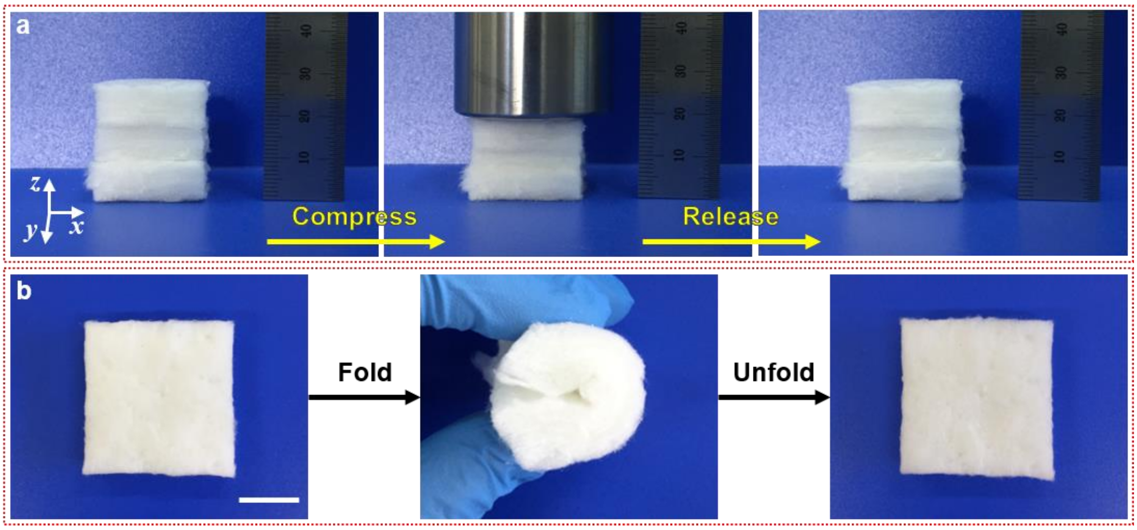

Supplementary Figure 33. **a** Photographs of a commercial ceramic fiber blanket before compression, during compression, and after release. **b** Photographs showing the flexibility of the commercial ceramic fiber blanket. Scale bar in **b**, 2 cm.

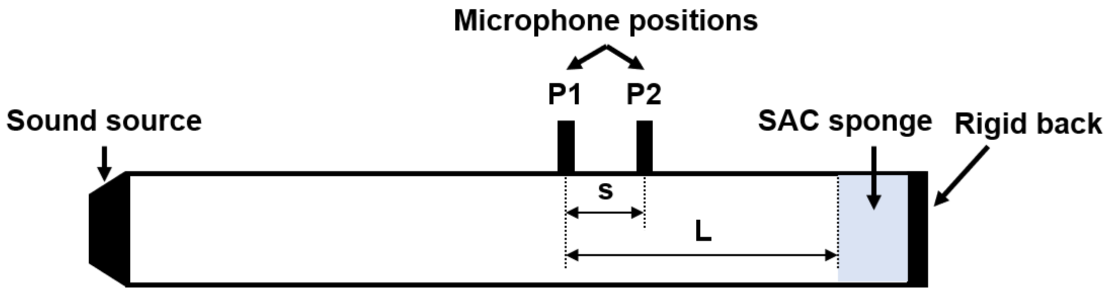

Supplementary Figure 34. Schematic showing the determination principle of the sound absorption property using impendence tubes.

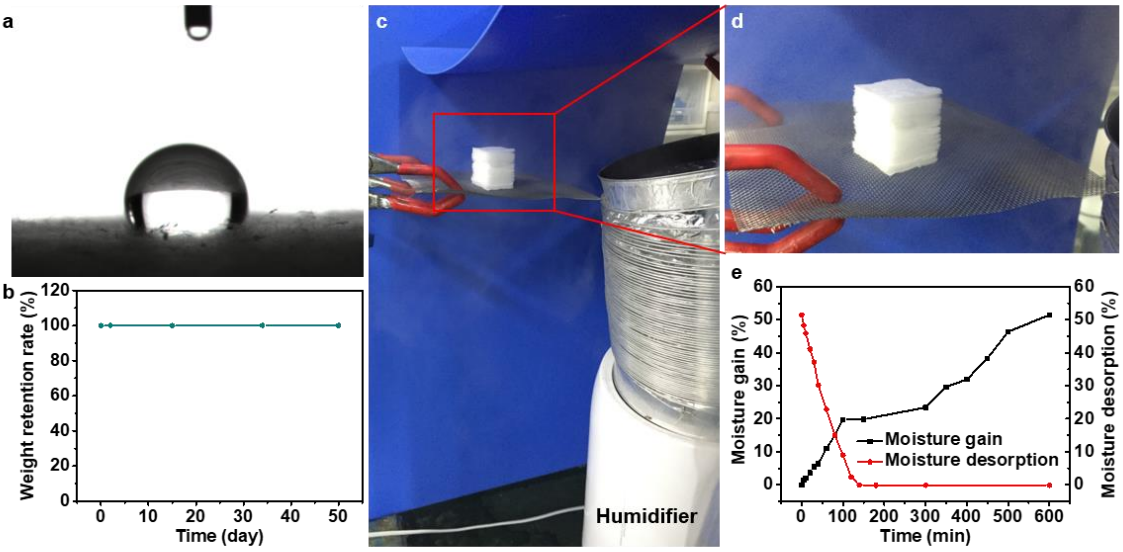

Supplementary Figure 35. Moisture resistance property of the SAC sponges. **a** Water contact angle of the SAC sponges. **b** Weight retention rate of the SAC sponges kept at ambient environment for different time. **c** Optical image of the setup for the moisture resistance property determination at high-humidity atmosphere. **d** Optical image of the SAC sponge in the water mist produced by a commercial humidifier. **e** Moisture gain and desorption properties of the SAC sponges.

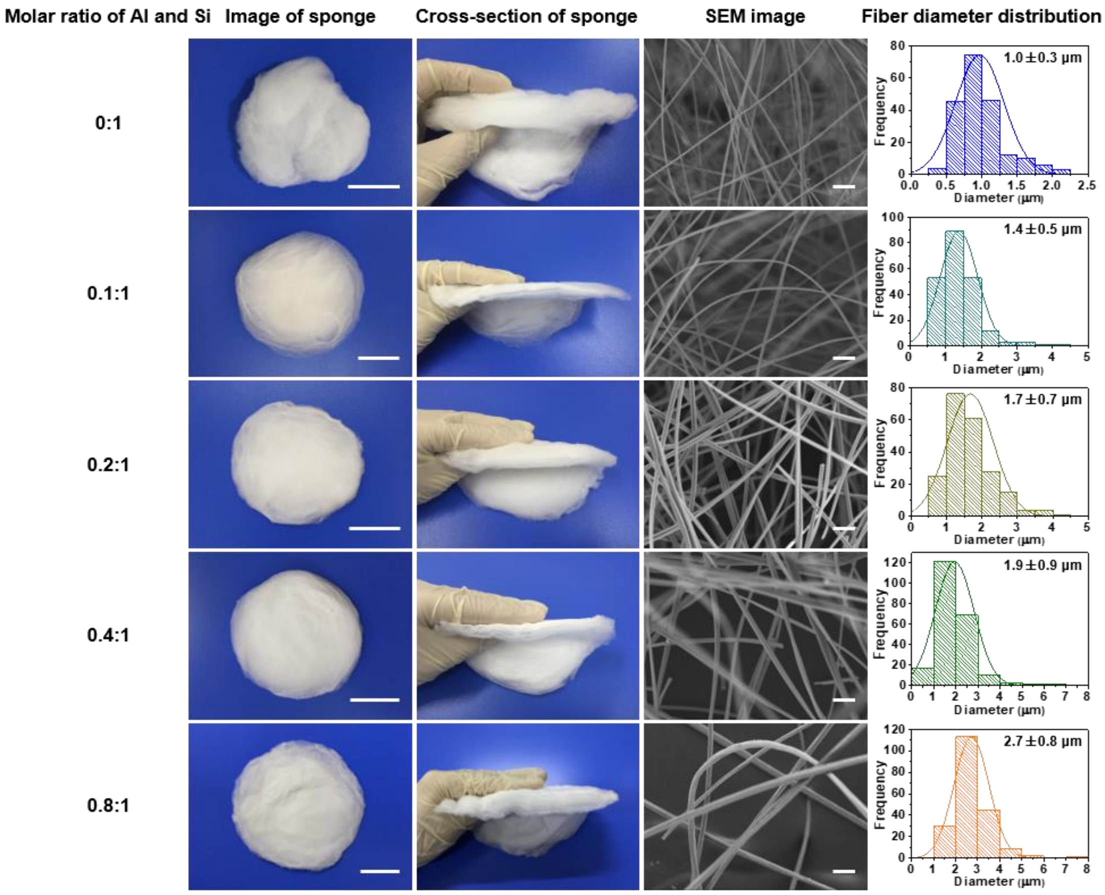

Supplementary Figure 36. Optical images of the sponges prepared from spinning solutions with different molar ratio of Al and Si, and SEM images and diameter distribution of microfibers. Scale bars in the images of sponge, 5 cm. Scale bars in the SEM images, 10 μm.

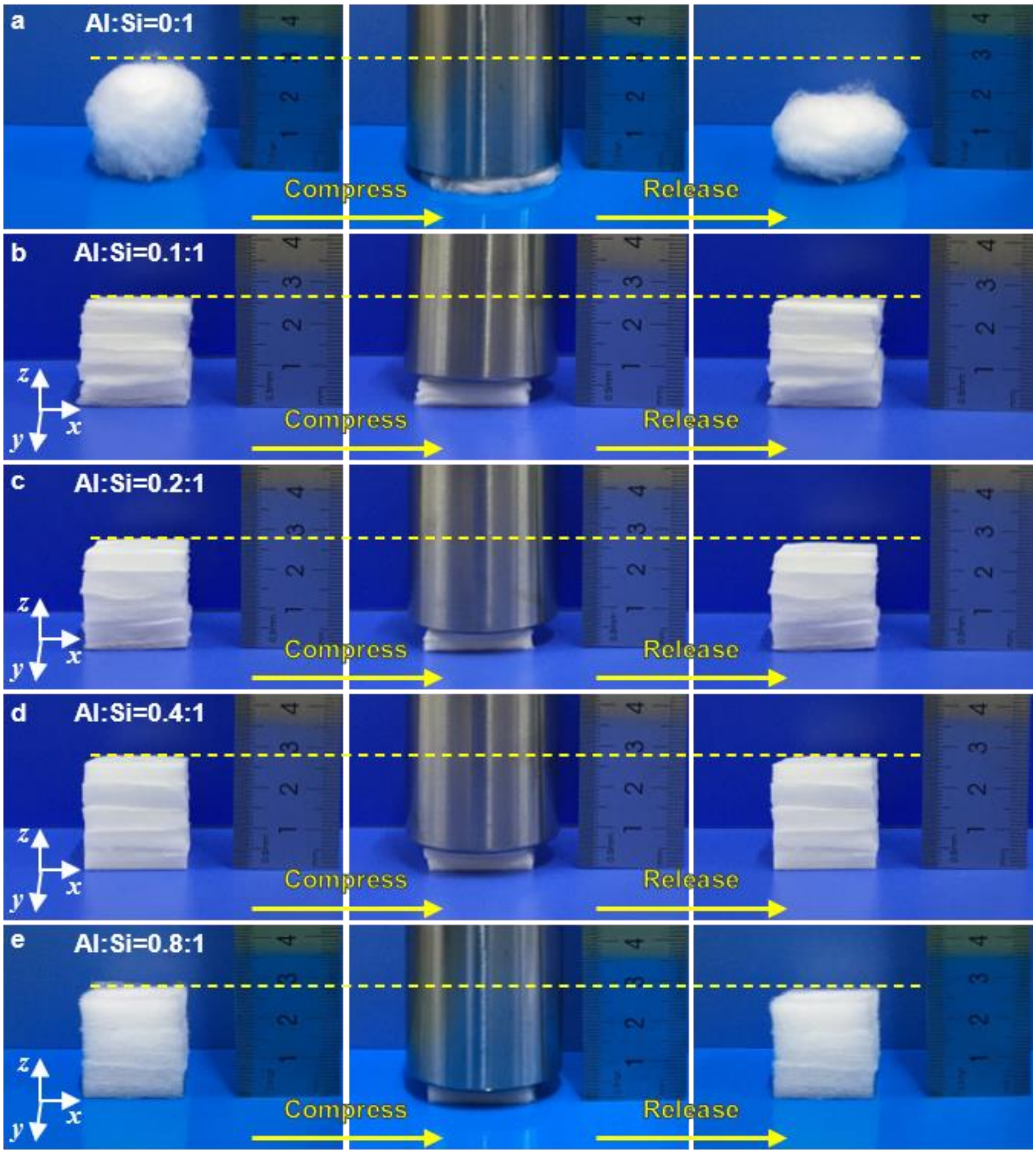

Supplementary Figure 37. Optical images of the sponges prepared from spinning solutions with different molar ratio of Al and Si to show their compression resilience. Molar ratio of Al and Si: **a** 0:1, **b** 0.1:1, **c** 0.2:1, **d** 0.4:1, **e** 0.8:1.

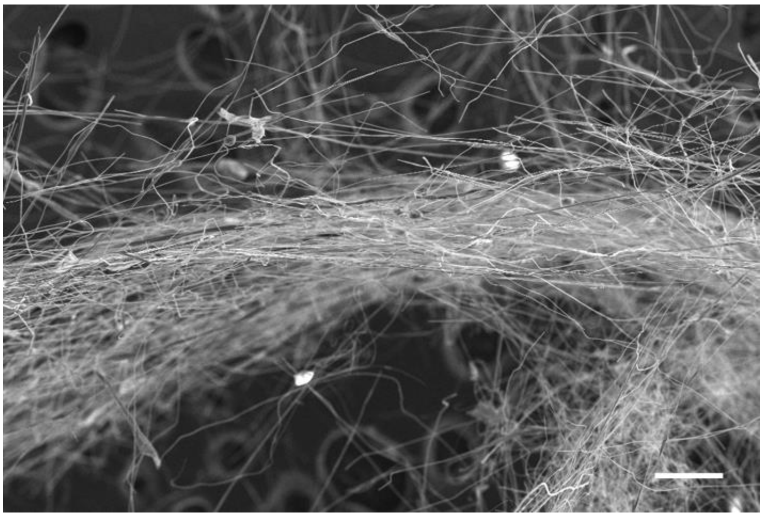

Supplementary Figure 38. SEM image of the single fiber layer peeled off from the SAC sponge. Scale bar, 200 μm.

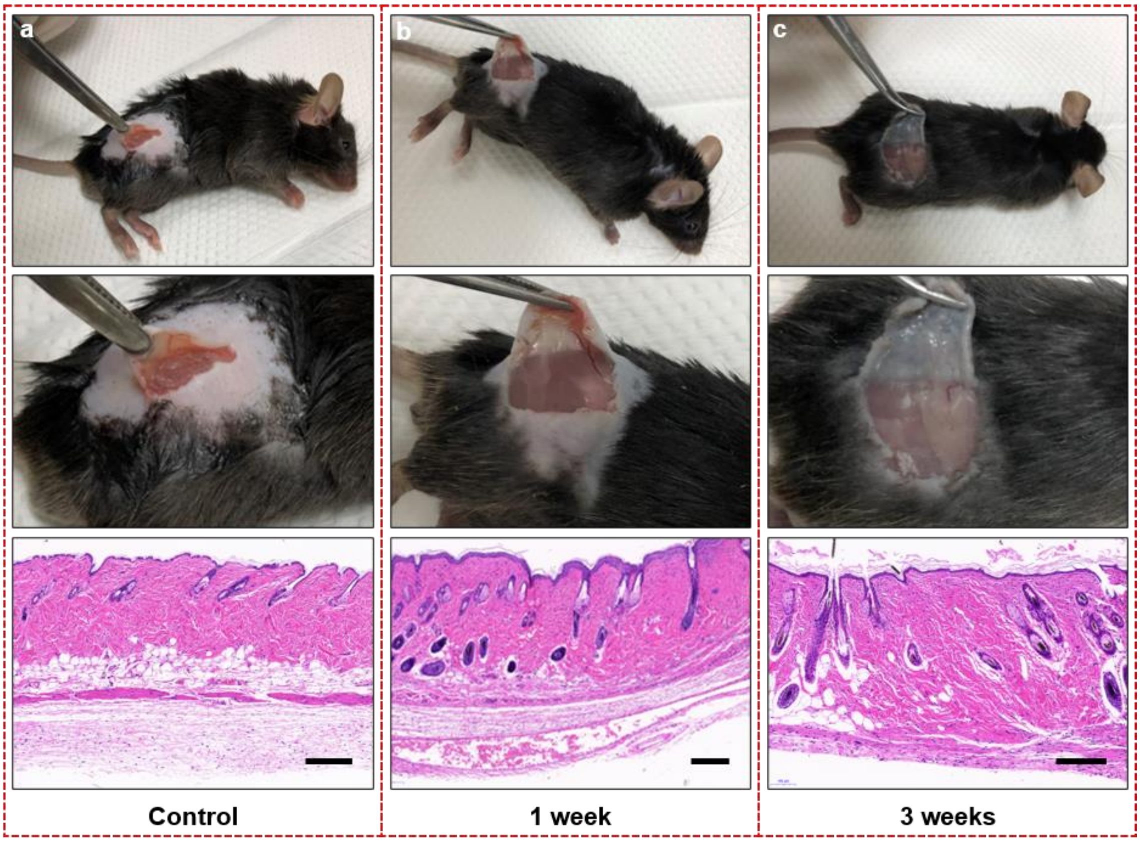

Supplementary Figure 39. Biocompatibility evaluation of the SAC sponge. Photographs of the subcutaneous region (top) and histological sections stained with hematoxylin and eosin (H&E) (bottom) in the C57 black 6 (C57BL/6N) mice **a** without and **b, c** with the implantation of SAC sponge after 1 week and 3 weeks. Scale bars in **a-c**, 200 μm.

Supplementary Tables

Supplementary Table 1. Comparison of the preparation methods and properties of ceramic sponges.

Preparation method	Material components	Preparation process		Property					Application potential		Reference
		Preparation procedure	Drying method	Density (mg cm^{-3})	Thermal conductivity (W m^{-1} K^{-1})	Compressibility	Flexibility	Maximum working temperature (Time) (°C)	Cost	Scalability	
Solution blow spinning	SiO_2-Al_2O_3	Blow spinning+Calcination	NA[a]	10	0.034	600 cycles at 50% strain, 88.3% stress retention	Good	1000 (>24 h)	Low	High	This work
Electrospun fiber reconstruction	Al, B-doped SiO_2	Nanofiber preparation+Dispersion+Ice formation+Freeze drying+Calcination	Freeze drying	0.15	0.025	500 cycles at 60% strain, 70% stress retention	NA	1100 (NA)	High	Low	1
Chemical vapor deposition	Boron nitride	Template preparation+Chemical vapor deposition+Crystalization in Ar+Thermal etch	NA	0.1	0.02	100 cycles at 90% strain, 90% stress retention	NA	900 (NA)	High	Low	2
Hydrogen bonding assembly	Boron nitride	Ultrasonic dispersion+Freeze drying+Calcination in NH_3 at 1200°C	Freeze drying	15.5	0.0346	50 cycles at 40% strain, 90% stress retention	Good	1000 (NA)	High	Low	3
Atomic layer deposition	Al_2O_3	Nanofiber preparation+Atomic layer deposition+Calcination	NA	0.68	0.022	100 cycles at 40% strain, 77% stress retention	NA	900 (2 h)	High	Low	4
Melt spinning	Aluminosilicate	Melt spinning+Double-sided needling process	NA	118	0.038	Poor	Good	1000 (for continuous use)	Low	High	Commercial product

[a] NA: Not applicable or not available.

Supplementary Table 2. Comparison of the sound absorption properties of our SAC sponges with other sound absorption materials.

Material	Areal density (mg cm^{-2})	Noise reduction coefficient (NRC)	References
SAC sponges	58	0.77	Our work
Kenaf	300	0.50	5
Wood	600	0.66	5
Mineralized wood	780	0.21	5
Hemp	150	0.39	5
Coconut	300	0.49	5
Straw	600	0.69	5
Cane	1600	0.36	5
Cardboard	1610	0.48	5
Sheep wool	160	0.53	5
Cork	300	0.26	5
Ramie	1772	0.60	6
Jute	1644	0.65	6
Flax	1364	0.65	6
Kenaf	74	0.11	7
Regular fiber polyester	83	0.11	7
Hollow fiber polyester	77	0.13	7
Jute	73	0.13	7
PAN/PVB/PET	21	0.41	8
PAN/PVB/PET	14	0.37	8
PAN/PVB/PET	27	0.53	8
Commercial melamine foam	27	0.30	8
Commercial nonwoven felt	53	0.43	8
PLA	113	0.21	9
PP	137	0.25	9
Coir	325	0.27	10
Coir	455	0.37	10
Coir	585	0.54	10

Polyamide	54	0.15	11
Kapok	50	0.66	12
Kapok	150	0.67	12
Kapok	252	0.61	12
Kapok	348	0.45	12
Yucca Gloriosa	300	0.18	13
Yucca Gloriosa	600	0.40	13
Wool	22	0.25	14
Wool	35	0.30	14
Wool	45	0.45	14
Wool	90	0.80	14
Wool	108	0.50	14
PET/sodium silicate	0.88	0.53	15
Polyurethane	120	0.55	16
Bamboo	600	0.68	17
Glass wool	160	0.62	17
Mineral wool	232	0.69	18
Porous metal	376	0.04	19
Porous metal	667	0.04	19
Porous metal	521	0.08	19
PAN/SiO_2	4.8	0.22	20
Melamine/GO	62.7	0.59	21

Supplementary Notes

Histological examination for toxicity test

All procedures of the toxicity test followed the institutional guidelines of Tsinghua University. The Institutional Animal Care and Use Committee (IACUC) of Tsinghua University approved the protocol. A single fiber layer peeled off from the SAC sponge was implanted in the subcutaneous tissues of mice under inhalational anesthesia. After administration of ketamine, xylazine and acepromazine, transcardial perfusion was performed with PBS and then 4% paraformaldehyde in the mice. The skin tissue around the fibers was collected and fixed in formaldehyde for 2 days. After embedded in paraffin, the tissue slices with a thickness of 4 μm were obtained using a Leica RM2016 slicer and stained with hematoxylin and eosin (H&E). The photographs of the histological sections were taken using an optical microscope (Nikon, Eclipse E100) and a digital camera (Nikon, DS-U3).

Moisture resistance property determination

The moisture resistance property of the sponges was characterized by placing them in the outlet of a commercial humidifier for moisture absorption and then keeping them in an atmosphere with a relative humidity of ~60% and a temperature of ~25°C for moisture desorption. The water content (w) in the sponges was calculated using the following equation: $w(\%) = \frac{m_2 - m_1}{m_1} \times 100\%$, where m_1 is the initial weight of the samples, and m_2 is the weight of the samples after moisture absorption.

Sound absorption property determination

The sound absorption properties were measured using impedance tubes (SW477, SW422, BSWA Technology Co., Ltd) in the frequency range of 63-6300 Hz according to the test criteria of ISO 10534-2:1998. The sponges with diameters of 100 mm and 30 mm were used to determine the sound absorption coefficients in the frequency ranges of 63-1600 Hz and 1000-6300 Hz, respectively.

The determination principle of the sound absorption property using impendence tubes is shown in Supplementary Fig. 34[8]. The transfer function $H_{12} = P_2/P_1$ between the two microphone positions P1 and P2 was obtained, and the reflection coefficient (r) was calculated according to the following equation:

$$r = \frac{H_{12} - e^{-jk_0 s}}{e^{jk_0 s} - H_{12}} e^{2jk_0 L} \qquad (1)$$

where s (m) is the distance between the two microphones, and L (m) is the distance between the microphone #1 and the front of the sponge. j is the imaginary unit ($j^2 = -1$). k_0 (m^{-1}) is the wavenumber in air which is expressed as: $k_0 = \omega/c_0$, $\omega = 2\pi f$, where ω (Hz) denotes the pulsation related to the frequency f (Hz), c_0 is the sound velocity (340 m s^{-1}) in air. The absorption coefficient at normal incidence is calculated according to the following equation:

$$\alpha = 1 - |r|^2 \qquad (2)$$

Molecular dynamics simulation

For molecular dynamics (MD) simulation, Newton's law was applied to describe the atomic interactions and MD was used to predict failure at the nanoscale by understanding the atomistic behavior of materials. MD simulations in this work were carried out using LAMMPS (large-scale atomic/molecular massively parallel simulator), an open-source software package[22], to investigate the mechanical properties of single fibers. The results were visualized using OVITO software (Open Visualization Tool)[23].

Force field

In this work, the Matsui force field was selected for the structural analysis of the ceramic fibers at both high and low temperatures[24, 25]. The Born-Mayer-Huggins potential V was used to express the interaction between atoms, as shown in equation 3. The three parts in the force field equation represent Coulombic, repulsive, and van der Waals interactions, respectively.

$$V = \frac{q_i q_j}{r_{ij}} + f(B_i + B_j) exp\left(\frac{(A_i + A_j - r_{ij})}{B_i + B_j}\right) - \frac{C_i C_j}{r_{ij}^6} \qquad (3)$$

where i and j are the atom species, q_i is the net charge on the atom i, r_{ij} is the distance between the atoms i and j, f is a constant (4.184 kJ A^{-1} mol^{-1}), A_i is the repulsive radius for atom i, B_i is the softness parameter for atom i, C_i is the van der Waals coefficient for atom i. The relevant values are summarized in Supplementary Table 3.

Supplementary Table 3. Parameters of different elements for force field.

Element	q (e)	A (Å)	B (Å)	C (Å^3[kJ mol^{-1}]$^{1/2}$)
Al	1.417	0.785	0.034	36.82
Si	1.890	0.720	0.023	49.30
O	-0.945	1.821	0.138	90.61

Molecular dynamics modeling of the tensile tests

For the sake of simplification, the molar ratio of Al and Si in the SiO_2-Al_2O_3 composite was set at 1:1 during the simulation process. The SiO_2 and SiO_2-Al_2O_3 composite were made by quenching the crystal SiO_2 and SiO_2-Al_2O_3 samples. The crystal samples made of approximately 160,000 atoms (10×10×20 nm) were equilibrated at 5000 K for 20 picoseconds in an NVT (constant number of atoms, constant volume, and constant temperature) ensemble. Then the high temperature melts were quenched to 300 K continuously over a period of 40 picoseconds in an NPT (constant number of atoms, constant pressure, and constant temperature) ensemble under ambient pressure. Finally, the samples were relaxed at 300 K under atmospheric pressure for 10 picoseconds. The Nosé-Hoover thermostat and barostat were used to control the temperature and pressure, respectively. The time step used for all simulations was 0.5 femtosecond. Periodic boundary conditions were applied in all directions.

The mechanical properties of the SiO_2 fibers and SAC fibers were investigated by carrying out uniaxial tensile tests on the two samples. The samples were uniformly loaded in the z direction at 300 K at the strain rate of 2×10^8 s^{-1} until the maximum strain of 30% in an NPT ensemble.

Supplementary Discussion

Role of PVA in the spinning solution

In our work, PVA was used as a tackifier for TEOS and $AlCl_3$ to promote the gelation process and also used as a template for forming the microfibers during the blow spinning process. PVA was used to assist fiber forming during the blow spinning process, so we call PVA a template. Note that polymers have been widely used as template to prepare ceramic fibers[26-30]. PVA and ceramic precursors were uniformly mixed together in the spinning solution (Supplementary Fig. 1a), and PVA was decomposed and removed from the fibers during the calcination process. The successful removal of PVA can be confirmed by the TGA result (Fig. 5m). The uniform distribution of the ceramic components in the fibers can be confirmed by the elemental mapping images (Fig. 2g). Therefore, from these results, we confirmed that PVA was used as a template to help shape the ceramic fibers.

PVA was also used as a tackifier for TEOS and $AlCl_3$ to promote the gelation process. PVA-TEOS-$AlCl_3$ solution and TEOS-$AlCl_3$ solution were prepared and their viscosity was determined using a rheometer (Supplementary Fig. 2). The PVA-TEOS-$AlCl_3$ solution and TEOS-$AlCl_3$ solution are transparent, but they show different viscosity. The viscosity of the PVA-TEOS-$AlCl_3$ solution is much higher than that of the TEOS-$AlCl_3$ solution due to the addition of PVA. We cannot obtain fibers from the TEOS-$AlCl_3$ solution due to its low viscosity. Many studies have also shown that a low viscosity solution cannot yield fibers by spinning[31-33]. In addition, these results also confirmed that PVA was used as a template to assist fiber forming during the blow spinning process.

Interaction between the spinning solution components

We obtained the FTIR spectra of PVA, TEOS, $AlCl_3$ and PVA-TEOS-$AlCl_3$ spinning solution to investigate the interaction between the solution components. TEOS shows characteristic peaks at 1167 and 787 cm^{-1}, which correspond to the asymmetric stretching and

symmetric stretching vibrations of Si-O-C bonds. TEOS will be hydrolyzed in the spinning solution. We added some H_3PO_4 in the spinning solution to promote the hydrolysis reaction. During the hydrolysis process, Si-O-Si bond networks are formed due to the hydrolysis and condensation reactions of TEOS molecules, which is demonstrated by the disappearance of Si-O-C bonds at 1167 and 787 cm^{-1} and the presence of Si-O-Si bond at 1044 and 876 cm^{-1}. A new weak peak can be found at 1130 cm^{-1} in the spectrum of the spinning solution, which can be ascribed to the formation of new Si-O-C bond between PVA and TEOS through crosslinking[34]. In addition, a wide and weak peak at 717 cm^{-1} was found, which can be ascribed to Si-O-Al vibration[35, 36]. Al^{3+} from $AlCl_3$ can react with partially hydrolyzed TEOS to occupy the proton sites of silanol and form Si-O-Al bond[37].

We further explored the possible interaction among the components in the PVA-TEOS-$AlCl_3$ solution via ^{27}Al and ^{29}Si nuclear magnetic resonance (NMR) spectroscopy. A sharp peak at 0.3 ppm can be observed in the ^{27}Al spectrum (Supplementary Fig. 4), which can be ascribed to non-bonded but partly hydrolyzed, octahedral-coordinated Al ions[38-41]. The results indicate that Al^{3+} was hydrolyzed in the spinning solution, and octahedral-coordinated Al ions ($[Al(H_2O)_6]^{3+}$) were formed[35, 41]. The partly hydrolyzed but non-bonded Al ions will form associates connected by weak hydrogen bonds[38-40]. In addition, the very weak peak at 70.2 ppm can be attributed to tetrahedral coordinated Al[40, 41], and the amount of tetrahedral coordinated Al is very small.

The ^{29}Si spectrum of the PVA-TEOS-$AlCl_3$ solution shows three peaks at -107.8, -102.1 and -92.5 ppm. The peaks at -92.5 and -102.1 ppm correspond to the silicon of geminal silanol and single silanol, respectively[42], which proves the presence of Si-OH bond. The peak at -107.8 ppm can be attributed to the silicon of siloxane[43], which demonstrates the existence of Si-O-Si bond. These results also indicate that TEOS hydrolyzes in the spinning solution. In addition,

compared with pure silica gel[43, 44], the ^{29}Si spectrum of the PVA-TEOS-$AlCl_3$ solution shows a little of chemical shift, which can be ascribed to the formation of Si-O-Al bond[42].

Role of $AlCl_3$ serving as an acidic catalyst

We prepared PVA-TEOS solution and PVA-TEOS-$AlCl_3$ solution, and determined their pH using pH indicator papers. We found that the pH significantly decreases from ~5 to ~1 when added some $AlCl_3$ in the solution (Supplementary Fig. 5). Al^{3+} from $AlCl_3$ can react with partially hydrolyzed TEOS to release some hydrochloric acid[37], and the strong acid significantly reduces the pH of PVA-TEOS-$AlCl_3$ solution. Some studies have shown that the hydrolysis rate of TEOS increases with decreasing pH in the acidic range[45]. In addition, $AlCl_3$ is a well-known strong acid catalyst, and has been widely used in various organic reactions[42]. Therefore, $AlCl_3$ can serve as an acidic catalyst to promote the hydrolysis of TEOS in our work. The addition of $AlCl_3$ significantly reduces the hydrolysis time of TEOS, so we can obtain a transparent solution in a shorter time.

Comparison with commercial ceramic fiber blanket

The commercial ceramic fiber blanket has a density of 118 mg cm^{-3}, which is much higher than that of our sponge. High density is undesirable in some application fields, such as aerospace application. For melt spinning method, the raw materials need to be melted at ultrahigh temperature (the melting point of aluminosilicate is 1545°C), which leads to a lot of energy consumption. Although the obtained composite fibers from our method also need to be calcinated at high temperature, the calcination temperature is much lower than that used in melt spinning. We have demonstrated that the SAC sponge can be obtained at a calcination temperature of only 1000°C. Therefore, our method for fabricating ceramic fibers is more energy-efficient. In addition, compared with commercial ceramic fiber blanket, our sponges demonstrated better compressibility, and they can be compressed beyond 80% strain thanks to their high flexibility and porosity. They can completely recover to their original shape without

obvious dimensional change after pressure release (Fig. 3b). In contrast, the commercial ceramic fiber blanket can only be compressed 30% strain due to its high density although it has good flexibility (Supplementary Fig. 33). In summary, our sponges have an ultralow density, relatively higher energy efficiency, and better compressibility than commercial ceramic fiber blanket.

Supplementary References

1. Si, Y., Wang, X., Dou, L., Yu, J. & Ding, B. Ultralight and fire-resistant ceramic nanofibrous aerogels with temperature-invariant superelasticity. *Sci. Adv.* **4,** eaas8925 (2018).

2. Xu, X. et al. Double-negative-index ceramic aerogels for thermal superinsulation. *Science* **363,** 723-727 (2019).

3. Li, G. et al. Boron nitride aerogels with super-flexibility ranging from liquid nitrogen temperature to 1000°C. *Adv. Funct. Mater.* **29,** 1900188 (2019).

4. Xu, C. et al. Ultralight and resilient Al_2O_3 nanotube aerogels with low thermal conductivity. *J. Am. Ceram. Soc.* **101,** 1677-1683 (2018).

5. Berardi, U. & Iannace, G. Predicting the sound absorption of natural materials: best-fit inverse laws for the acoustic impedance and the propagation constant. *Appl. Acoust.* **115,** 131-138 (2017).

6. Yang, W. D. & Li, Y. Sound absorption performance of natural fibers and their composites. *Sci. China: Technol. Sci.* **55,** 2278-2283 (2012).

7. Na, Y. & Cho, G. Sound absorption and viscoelastic property of acoustical automotive nonwovens and their plasma treatment. *Fibers Polym.* **11,** 782-789 (2010).

8. Cao, L. et al. Ultralight, superelastic and bendable lashing-structured nanofibrous aerogels for effective sound absorption. *Nanoscale* **11,** 2289-2298 (2019).

9. Yilmaz, N. D., Banks-Lee, P., Powell, N. B. & Michielsen, S. Effects of porosity, fiber size, and layering sequence on sound absorption performance of needle-punched nonwovens. *J. Appl. Polym. Sci.* **121,** 3056-3069 (2011).

10. Taban, E., Tajpoor, A., Faridan, M., Samaei, S. E. & Beheshti, M. H. Acoustic absorption characterization and prediction of natural coir fibers. *Acoust. Aust.* **47,** 67-77 (2019).

11. Na, Y., Agnhage, T. & Cho, G. Sound absorption of multiple layers of nanofiber webs and the comparison of measuring methods for sound absorption coefficients. *Fibers Polym.* **13,** 1348-1352 (2012).

12. Xiang, H.-f., Wang, D., Liu, H.-c., Zhao, N. & Xu, J. Investigation on sound absorption properties of kapok fibers. *Chin. J. Polym. Sci.* **31,** 521-529 (2013).

13. Soltani, P., Taban, E., Faridan, M., Samaei, S. E. & Amininasab, S. Experimental and computational investigation of sound absorption performance of sustainable porous material: yucca gloriosa fiber. *Appl. Acoust.* **157,** 106999 (2020).

14. Ballagh, K. O. Acoustical properties of wool. *Appl. Acoust.* **48,** 101-120 (1996).

15. Mazrouei-Sebdani, Z., Khoddami, A., Hadadzadeh, H. & Zarrebini, M. Synthesis and performance evaluation of the aerogel-filled PET nanofiber assemblies prepared by electro-spinning. *RSC Adv.* **5,** 12830-12842 (2015).

16. Sung, G. & Kim, J. H. Effect of high molecular weight isocyanate contents on manufacturing polyurethane foams for improved sound absorption coefficient. *Korean J. Chem. Eng.* **34,** 1222-1228 (2017).

17. Koizumi, T., Tsujiuchi, N. & Adachi, A. The development of sound absorbing materials using natural bamboo fibers. in: *High Performance Structures and Composites* (WIT Press, Southampton, 2002).

18. Asdrubali, F. Survey on the acoustical properties of new sustainable materials for noise control. *Euronoise* **30,** (2006).

19. Wang, J. et al. Sound absorption performance of porous metal fiber materials with different structures. *Appl. Acoust.* **145,** 431-438 (2019).

20. Si, Y., Yu, J., Tang, X., Ge, J. & Ding, B. Ultralight nanofibre-assembled cellular aerogels with superelasticity and multifunctionality. *Nat. Commun.* **5,** 5802 (2014).

21. Nine, M. J. et al. Graphene oxide-based lamella network for enhanced sound absorption. *Adv. Funct. Mater.* **27,** 1703820 (2017).

22. Plimpton, S. Fast parallel algorithms for short-range molecular-dynamics. *J. Comput. Phys.* **117,** 1-19 (1995). Also see http://lammps.sandia.gov.

23. Stukowski, A. Visualization and analysis of atomistic simulation data with OVITO-the open visualization tool. *Modell. Simul. Mater. Sci. Eng.* **18,** 015012 (2010).

24. Matsui, M. Molecular dynamics study of the structures and bulk moduli of crystals in the system CaO-MgO-Al_2O_3-SiO_2. *Phys. Chem. Miner.* **23,** 345-353 (1996).

25. Adabifiroozjaei, E., Hart, J. N., Koshy, P., Mitchell, D. R. G. & Sorrell, C. C. Mullite-glass and mullite-mullite interfaces: analysis by molecular dynamics (MD) simulation and high-resolution TEM. *J. Am. Ceram. Soc.* **101,** 428-439 (2018).

26. Yan, J. et al. Polymer template synthesis of flexible $BaTiO_3$ crystal nanofibers. *Adv. Funct. Mater.* **29,** 1907919 (2019).

27. Yan, J. et al. Polymer template synthesis of soft, light, and robust oxide ceramic films. *iScience* **15,** 185-195 (2019).

28. Wang, Y., Li, W., Jiao, X. L. & Chen, D. R. Electrospinning preparation and adsorption properties of mesoporous alumina fibers. *J. Mater. Chem. A* **1,** 10720-10726 (2013).

29. Wang, X. et al. Flexible hierarchical ZrO_2 nanoparticle-embedded SiO_2 nanofibrous membrane as a versatile tool for efficient removal of phosphate. *ACS Appl. Mater. Interfaces* **8,** 34668-34676 (2016).

30. Li, D. & Xia, Y. N. Fabrication of titania nanofibers by electrospinning. *Nano Lett.* **3,** 555-560 (2003).

31. Gupta, P., Elkins, C., Long, T. E. & Wilkes, G. L. Electrospinning of linear homopolymers of poly(methyl methacrylate): exploring relationships between fiber formation, viscosity, molecular weight and concentration in a good solvent. *Polymer* **46,** 4799-4810 (2005).

32. Vicini, S., Mauri, M., Vita, S. & Castellano, M. Alginate and alginate/hyaluronic acid membranes generated by electrospinning in wet conditions: relationship between solution viscosity and spinnability. *J. Appl. Polym. Sci.* **135,** 46390 (2018).

33. Nezarati, R. M., Eifert, M. B. & Cosgriff-Hernandez, E. Effects of humidity and solution viscosity on electrospun fiber morphology. *Tissue Eng., Part C* **19,** 810-819 (2013).

34. Pirzada, T., Arvidson, S. A., Saquing, C. D., Shah, S. S. & Khan, S. A. Hybrid silica-PVA nanofibers via sol-gel electrospinning. *Langmuir.* **28,** 5834-5844 (2012).

35. Wu, X., Shao, G., Cui, S., Wang, L. & Shen, X. Synthesis of a novel Al_2O_3-SiO_2 composite aerogel with high specific surface area at elevated temperatures using inexpensive inorganic salt of aluminum. *Ceram. Int.* **42,** 874-882 (2016).

36. Ling, X., Li, B., Li, M., Hu, W. & Chen, W. Thermal stability of Al-modified silica aerogels through epoxide-assisted sol-gel route followed by ambient pressure drying. *J. Sol-Gel Sci. Technol.* **87,** 83-94 (2018).

37. Chakrabarti, S. & Pramanik, P. Binary silicate gels using tetraethyl orthosilicate and weak acid salts. *J. Mater. Sci. Lett.* **9,** 1135-1136 (1990).

38. Sinkó, K., Mezei, R. & Zrinyi, M. Gelation of aluminosilicate systems under different chemical conditions. *J. Sol-Gel Sci. Technol.* **21,** 147-156 (2001).

39. Sinkó, K. & Neményi, A. Effect of Al- and Si-containing precursors on the structures formed by sol-gel methods. *Prog. Colloid Polym. Sci.* **125,** 103-110 (2004).

40. Sinkó, K., Mezei, R., Rohonczy, J. & Fratzl, P. Gel structures containing Al (III). *Langmuir.* **15,** 6631-6636 (1999).

41. Komarneni, S., Roy, R., Fyee, C. A., Kennedy, G. J. & Strobl, H. Solid-state ^{27}Al and ^{29}Si magic-angle spinning NMR of aluminosilicate gels. *J. Am. Ceram. Soc.* **69,** C-42-C-44 (1986).

42. Sato, S. & Maciel, G. E. Structures of aluminum chloride grafted on silica surface. *J. Mol. Catal. A: Chem.* **101,** 153-161 (1995).

43. Chuang, I.-S., Kinney, D. R., Bronnimann, C. E., Zeigler, R. C. & Maciel, G. E. Effects of 1H-1H spin exchange in the ^{29}Si CP-MAS NMR spectra of the silica surface. *J. Phys. Chem.* **96,** 4027-4034 (1992).

44. Maciel, G. E. & Sindorf, D. W. Silicon-29 nuclear magnetic resonance study of the surface of silica gel by cross polarization and magic-angle spinning. *J. Am. Chem. Soc.* **102,** 7606-7607 (1980).

45. Cihlář, J. Hydrolysis and polycondensation of ethyl silicates. 1. effect of pH and catalyst on the hydrolysis and polycondensation of tetraethoxysilane (TEOS). *Colloids Surf., A* **70,** 239-251 (1993).
