# Supplementary material for: Highly compressible and anisotropic lamellar ceramic sponges with superior thermal insulation and acoustic absorption performances
Source: Nat Commun. 2020 Jul 24;11:3732. doi: 10.1038/s41467-020-17533-6 (PMC7382455; doi:10.1038/s41467-020-17533-6)
Supplement: Supplementary file 2 — Peer Review File [file 41467_2020_17533_MOESM2_ESM.docx]

Peer Review Information

**Journal:** Nature Communications

**Manuscript title:** Highly compressible and anisotropic lamellar ceramic sponges with superior thermal insulation and acoustic absorption performances

**Corresponding author name(s):** Prof Hui Wu

**Editorial notes:**

**Reviewer comments & decisions:**

**Reviewer comments, first version:**

Reviewer #1 (Remarks to the Author):

This paper deals with preparation and characterization of porous ceramic thin fibrous materials by using blow spinning. Scalable preparation method of thin fibers is interesting. However, blow spinning is not a novel technique and SiO2-Al2O3 composite is also not a novel material. I cannot understand the novelty and advantages of “ceramic sponge” as a functional material. The authors should discuss the novelty and/or outstanding functions of anisotropic, lamellar ceramic fiber sponges based on the experimental data in addition to their preparation and fundamental properties. I strongly recommend that the authors should resubmit their completed research including outstanding performances of the advanced ceramic sponges in the fields such as thermal insulators, energy absorbers, catalyst carriers, and high temperature air filters. Other concerns are below.


Title: Anisotropic, Lamellar Ceramic Fiber Sponges with Temperature-Invariant High Compressibility from -196 to 1000°C
I think the tile is misleading. In the present manuscript, the compressibility in liquid nitrogen is reported, while the compressibility of the samples after treatment under the high temperature conditions is reported (not under the high temperature conditions).


Reviewer #2 (Remarks to the Author):

Authors have reported fabrication of highly compressible, temperature resistant SiO2-Al2O3 composite ceramic sponges via blow spinning a polyvinyl alcohol (PVA)-ceramic precursor solution and subsequent calcination, the detailed mechanical property of the resulting composite ceramic sponges has been performed and the thermal property of the obtained samples have been demonstrated. Temperature resistant (from liquid nitrogen temperature to 1000 degree C) ceramic materials have been found from literatures recently, and silica-alumina composite ceramics have been investigated deeply. Authors have reported compressible silica-alumina composite ceramics in their manuscript, but only given similar results reported from other ceramic materials. In such a way, it seems that the analysis in this manuscript is superficial. The detailed comments are as follows:
1. What is the role of the PVA in this process? Serving as the molecular template for forming the microfibers? What is the interaction among PVA, TEOS and AlCl3?
2. What is the specific role for AlCl3? In the absence of the AlCl3, the products did not show good flexibility. Does it ascribe to molecular mechanism or structural mechanism? If we tune the mass ratios of TEOS to AlCl3, what will we observe?
3. What is the intrinsic flexibility mechanism of the silica-alumina composite ceramic sponges? How to avoid plastic deformation during stress? Is it ascribing to chemical or structural mechanism? Could authors give chemical or structural model to simulate? I remind authors to pay much attention to the joint points of the composite fibers.
4. Please measure the thermal conductivity along different directions to see whether samples possess anisotropic thermal property
5. Home-made hot-wire method has been used to measure the thermal conductivity. Please report the calibration results of authors home-made apparatus in the revised manuscript. In addition, hot-wire only gives the in-plane thermal conductivity of the samples, it is not suitable for the samples reported by authors. I suggest authors give the out-of-plane thermal conductivity result.
6. In the sentence “which is comparable to those of other inorganic aerogels”, why authors compare the physical properties of their samples with those of aerogels? A lot of such comparison can be seen. What is the relationship between sponge and aerogel? Why not compare those with that of any other spongy materials or other lightweight materials?


Reviewer #3 (Remarks to the Author):

The authors developed highly compressible, temperature resistant SiO2-Al2O3 composite ceramic sponges by blow spinning polyvinyl alcohol-ceramic precursor solution and subsequent calcination. The sponges have a unique anisotropic lamellar structure, which provides the sponges with excellent mechanical compressibility, high compression fatigue resistance and robust mechanical stability. In addition, the composite sponges possess robust fire resistance, temperature-invariant compression resilience and excellent thermal insulation property. The advantages of their preparation method have been highlighted by comparison with the most commonly used ceramic sponge preparation methods.
The authors have presented a well written paper supported by numerous figures both in the paper and in the Supplemental Information. The properties of the sponges were thoroughly and satisfactorily characterized and discussed, and the results are impressive. Taking these points into account, I consider that the manuscript is of interest to the readers of Nature Communications and I recommend this work to be accepted for publication after minor revision.

1. Are the sponges hydrophobic? I wonder if the sponges will absorb water in air? It will be more interesting to give some data about that.
2. I'm very concerned about the stability of the sponges. Whether the sponges can maintain good compression resilience after a long-term storage, such as 1 or 2 months? The authors should give some data to prove this point.

**Author rebuttal, first version:**

**Reviewer #1**

**Comment 1:** *This paper deals with preparation and characterization of porous ceramic thin fibrous materials by using blow spinning. Scalable preparation method of thin fibers is interesting. However, blow spinning is not a novel technique and SiO2-Al2O3 composite is also not a novel material. I cannot understand the novelty and advantages of “ceramic sponge” as a functional material. The authors should discuss the novelty and/or outstanding functions of anisotropic, lamellar ceramic fiber sponges based on the experimental data in addition to their preparation and fundamental properties. I strongly recommend that the authors should resubmit their completed research including outstanding performances of the advanced ceramic sponges in the fields such as thermal insulators, energy absorbers, catalyst carriers, and high temperature air filters.*

**Response:** We thank the reviewer for the constructive comments.

We agree with the reviewer that it is very important to report more functions of our materials. Accordingly, we have applied our SAC sponges in the field of acoustic absorption (sound energy absorber), and demonstrated excellent sound absorption performance of the ceramic sponges.

Corresponding changes:

Page 1: **Title: Highly Compressible, Anisotropic, Lamellar Ceramic Sponges with Superior Thermal Insulation and Acoustic Absorption Performances**

Page 4: In addition, we also demonstrated that the lightweight SAC sponges with a density of 20 mg cm^-3^ and a thickness 29 mm possess excellent sound absorption properties (NRC value of 0.77).

Pages14-16:

**Acoustic absorption property**

Noise pollution from vehicles, machinery equipment, engineering construction has become a major health and environmental concern, and eliminating or reducing noise pollution is of great importance to human life. Sound absorption materials can be used to consume sound energy in the process of noise transmission, and some efficient sound absorption materials have been developed in recent years45, ^46^. However, simultaneous achievement of excellent thermal insulation and acoustic absorption remains a huge challenge.

In addition to good thermal insulation properties, we also demonstrated that our SAC sponges possess excellent sound absorption property. The sound absorption properties were measured using impedance tubes in the frequency range of 63-6300 Hz according to the test criteria of ISO 10534-2:1998 (Supplementary Fig. 31 and Supplementary Methods). The SAC sponges were cut into cylinders with diameters of 100 mm and 30 mm for the determination of sound absorption properties in the frequency ranges of 63-1600 Hz and 1000-6300 Hz, respectively (Fig. 7a). The absorption coefficient of the SAC sponges increases sharply with the increase of sound frequency, specifically for the SAC sponge with a thickness of 29 mm. The effect of thickness on the sound absorption properties was investigated, and the results are shown in Figs 7b-c. The sound absorption coefficient of the SAC sponges increases with the increasing thickness, which can be explained by the increased sound propagation path. The sound absorption properties of porous materials can be evaluated by noise reduction coefficient (NRC), which is the average of the sound absorption coefficients at 250, 500, 1000 and 2000 Hz. The NRC value significantly increases from 0.05 for the SAC sponge with a thickness of 4 mm to 0.77 for the SAC sponge with a thickness of 29 mm.

We compared the sound absorption property of our SAC sponges with that of other sound absorption materials in terms of NRC and areal density (Fig. 7d and Supplementary Table 2)^29,^ 45, 47-61. Our SAC sponges show lightweight characteristic and better sound absorption property. In addition, compared with commercial melamine foam and nonwoven felt with similar thickness, the SAC sponges also exhibit better sound absorption property^45^. We ascribe the excellent sound absorption properties of our SAC sponges to the following reasons. First, the SAC sponges possess a lamellar structure, which leads to multiple absorption of sound waves

between layers (Fig. 7e). Second, the microfibers in our SAC sponges have a rough surface (Fig. 2c), which can increase the friction between sound waves and our materials, thus causing more consumption of sound energy during the sound propagation progress.

Moisture resistance property is important for the practical application of sound absorption materials. Our SAC sponges exhibit a water contact angle of 128° (Supplementary Fig. 32a), which can prevent them from swelling and maintain the stable acoustic absorption properties. We placed our SAC sponges in ambient environment, and weighed them at different time to determine the weight retention rate. The weight of the samples remained stable at different times and no weight change can be observed (Supplementary Fig. 32b), indicating that our sponges do not absorb water in air. The moisture resistance property of our sponges was further demonstrated by moisture absorption and desorption test (Supplementary Figs 32c and 32d, Supplementary Methods). The weight of the samples was increased by 51% after exposing them in a high-humidity atmosphere for 10 hours. The adsorbed water in the samples was desorbed quickly when keeping them in ambient environment with a relative humidity of 60% and the samples returned to their original weight in about 2 hours (Supplementary Fig. 32e), indicating that the SAC sponges possess good moisture resistance property. Water is mainly distributed on the surface of the fibers due to their hydrophobic property, which results in the rapid moisture desorption.

Page 21: Water contact angles (WCA) of the sponges were determined using a contact angle goniometer (JCY, Shanghai Fangrui Instrument Co., Ltd, China).

Page 38:

**Figure 7.** Acoustic absorption properties of the SAC sponges. (a) Optical images of the SAC sponges for the acoustic absorption property determination. (b) Sound absorption coefficient of the SAC sponges with different thicknesses. (c) Noise reduction coefficient (NRC) of the SAC sponges with different thicknesses. (d) Comparison of the sound absorption properties of our SAC sponges with other sound absorption materials. (e) Schematic showing the sound transmission though the SAC sponges.

Page 36 in Supplementary Information:

**Supplementary Figure 31.** Schematic showing the determination principle of the sound absorption property using impendence tubes.

Page 37 in Supplementary Information:

**Supplementary Figure 32.** Moisture resistance property of the SAC sponges. (a) Water contact angle of the SAC sponges. (b) Weight retention rate of the SAC sponges kept at ambient environment for different time. (c) Optical image of the setup for the moisture resistance property determination at high-humidity atmosphere. (d) Optical image of the SAC sponge in the water mist produced by a commercial humidifier. (e) Moisture gain and desorption properties of the SAC sponges.

Pages 43-44 in Supplementary Information:

**Supplementary Table 2.** Comparison of the sound absorption properties of our SAC sponges

with other sound absorption materials.

| Material | Areal density (mg  cm^-2^) | Noise reduction  coefficient (NRC) | References |
| --- | --- | --- | --- |
| SAC sponges | 58 | 0.77 | Our work |
| Kenaf | 300 | 0.50 | 5 |
| Wood | 600 | 0.66 | 5 |
| Mineralized wood | 780 | 0.21 | 5 |
| Hemp | 150 | 0.39 | 5 |
| Coconut | 300 | 0.49 | 5 |

| Straw | 600 | 0.69 | 5 |
| --- | --- | --- | --- |
| Cane | 1600 | 0.36 | 5 |
| Cardboard | 1610 | 0.48 | 5 |
| Sheep wool | 160 | 0.53 | 5 |
| Cork | 300 | 0.26 | 5 |
| Ramie | 1772 | 0.60 | 6 |
| Jute | 1644 | 0.65 | 6 |
| Flax | 1364 | 0.65 | 6 |
| Kenaf | 74 | 0.11 | 7 |
| Regular fiber polyester | 83 | 0.11 | 7 |
| Hollow fiber polyester | 77 | 0.13 | 7 |
| Jute | 73 | 0.13 | 7 |
| PAN/PVB/PET | 21 | 0.41 | 8 |
| PAN/PVB/PET | 14 | 0.37 | 8 |
| PAN/PVB/PET | 27 | 0.53 | 8 |
| Commercial melamine foam | 27 | 0.30 | 8 |
| Commercial nonwoven felt | 53 | 0.43 | 8 |
| PLA | 113 | 0.21 | 9 |
| PP | 137 | 0.25 | 9 |
| Coir | 325 | 0.27 | 10 |
| Coir | 455 | 0.37 | 10 |
| Coir | 585 | 0.54 | 10 |
| Polyamide | 54 | 0.15 | 11 |
| Kapok | 50 | 0.66 | 12 |
| Kapok | 150 | 0.67 | 12 |
| Kapok | 252 | 0.61 | 12 |
| Kapok | 348 | 0.45 | 12 |
| Yucca Gloriosa | 300 | 0.18 | 13 |
| Yucca Gloriosa | 600 | 0.40 | 13 |

| Wool | 22 | 0.25 | 14 |
| --- | --- | --- | --- |
| Wool | 35 | 0.30 | 14 |
| Wool | 45 | 0.45 | 14 |
| Wool | 90 | 0.80 | 14 |
| Wool | 108 | 0.50 | 14 |
| PET/sodium silicate | 0.88 | 0.53 | 15 |
| Polyurethane | 120 | 0.55 | 16 |
| Bamboo | 600 | 0.68 | 17 |
| Glass wool | 160 | 0.62 | 17 |
| Mineral wool | 232 | 0.69 | 18 |
| Porous metal | 376 | 0.04 | 19 |
| Porous metal | 667 | 0.04 | 19 |
| Porous metal | 521 | 0.08 | 19 |
| PAN/SiO2 | 4.8 | 0.22 | 20 |
| Melamine/GO | 62.7 | 0.59 | 21 |

Pages 45-46 in Supplementary Information:

**Supplementary Methods**

**Moisture resistance property determination**

The moisture resistance property of the sponges was characterized by placing them in the

outlet of a commercial humidifier for moisture absorption and then keeping them in an

atmosphere with a relative humidity of ~60% and a temperature of ~25°C for moisture

desorption. The water content (*w*) in the sponges was calculated using the following equation:

| *(%) = 2−1*  *1* | *× 100%*, where *m*1 is the initial weight of the samples, and *m*2 is the weight |
| --- | --- |

of the samples after moisture absorption.

**Sound absorption property determination**

The sound absorption properties were measured using impedance tubes (SW477, SW422,

BSWA Technology Co., Ltd) in the frequency range of 63-6300 Hz according to the test criteria

of ISO 10534-2:1998. The sponges with diameters of 100 mm and 30 mm were used to

determine the sound absorption coefficients in the frequency ranges of 63-1600 Hz and 1000­6300 Hz, respectively.

The determination principle of the sound absorption property using impendence tubes is shown in Supplementary Fig. 31^8^. The transfer function *H*12 = *P*2/*P*1 between the two microphone positions P1 and P2 was obtained, and the reflection coefficient (*r*) was calculated according to the following equation:

12−^−0^

= 0−12 20	(1)

where *s* (m) is the distance between the two microphones, and *L* (m) is the distance between the microphone #1 and the front of the sponge. *j* is the imaginary unit (*j*^2^ = -1). *k*0 (m^-1^) is the wavenumber in air which is expressed as: *k*0 = *co*/*c*0, *co = 2πf,* where *co* (Hz) denotes the pulsation related to the frequency *f* (Hz), c0 is the sound velocity (340 m s^-1^) in air. The absorption coefficient at normal incidence is calculated according to the following equation:

*α* = 1− |*r*|^2^	(2)

Pages 50-52 in Supplementary Information:
**Supplementary References:**

5. Berardi, U., Iannace, G. Predicting the sound absorption of natural materials: Best-fit inverse laws for the acoustic impedance and the propagation constant. *Appl. Acoust.* **115**, 131­138 (2017).
6. Yang, W. D., Li, Y. Sound absorption performance of natural fibers and their composites. *Sci. China: Technol. Sci.* **55**, 2278-2283 (2012).
7. Na, Y., Cho, G. Sound absorption and viscoelastic property of acoustical automotive nonwovens and their plasma treatment. *Fibers Polym.* **11**, 782-789 (2010).
8. Cao, L., Si, Y., Wu, Y., Wang, X., Yu, J., Ding, B. Ultralight, superelastic and bendable lashing-structured nanofibrous aerogels for effective sound absorption. *Nanoscale* **11**, 2289­2298 (2019).
9. Yilmaz, N. D., Banks-Lee, P., Powell, N. B., Michielsen, S. Effects of porosity, fiber size, and layering sequence on sound absorption performance of needle-punched nonwovens. *J. Appl. Polym. Sci.* **121**, 3056-3069 (2011).
10. Taban, E., Tajpoor, A., Faridan, M., Samaei, S. E., Beheshti, M. H. Acoustic absorption characterization and prediction of natural coir fibers. *Acoust. Aust.* **47**, 67-77 (2019).
11. Na, Y., Agnhage, T., Cho, G. Sound absorption of multiple layers of nanofiber webs and the comparison of measuring methods for sound absorption coefficients. *Fibers Polym.* **13**, 1348-1352 (2012).
12. Xiang, H.-f., Wang, D., Liu, H.-c., Zhao, N., Xu, J. Investigation on sound absorption properties of kapok fibers. *Chin. J. Polym. Sci.* **31**, 521-529 (2013).
13. Soltani, P., Taban, E., Faridan, M., Samaei, S. E., Amininasab, S. Experimental and computational investigation of sound absorption performance of sustainable porous material: Yucca gloriosa fiber. *Appl. Acoust.* **157**, (2020).
14. Ballagh, K. O. Acoustical properties of wool. *Appl. Acoust.* **48**, 101-120 (1996).
15. Mazrouei-Sebdani, Z., Khoddami, A., Hadadzadeh, H., Zarrebini, M. Synthesis and performance evaluation of the aerogel-filled pet nanofiber assemblies prepared by electro-spinning. *RSC Adv.* **5**, 12830-12842 (2015).
0. Sung, G., Kim, J. H. Effect of high molecular weight isocyanate contents on manufacturing polyurethane foams for improved sound absorption coefficient. *Korean J. Chem. Eng.* **34**, 1222-1228 (2017).
16. Koizumi, T., Tsujiuchi, N., Adachi, A. The development of sound absorbing materials using natural bamboo fibers. In: *High Performance Structures and Composites* (ed^(eds Brebbia CA, DeWilde WP) (2002).
17. Asdrubali, F. Survey on the acoustical properties of new sustainable materials for noise control. *Euronoise* **30**, (2006).
18. Wang, J. et al. Sound absorption performance of porous metal fiber materials with different structures. *Appl. Acoust.* **145**, 431-438 (2019).
19. Si, Y., Yu, J., Tang, X., Ge, J., Ding, B. Ultralight nanofibre-assembled cellular aerogels with superelasticity and multifunctionality. *Nat. Commun.* **5**, 5802 (2014).
20. Nine, M. J., Ayub, M., Zander, A. C., Tran, D. N. H., Cazzolato, B. S., Losic, D. Graphene oxide-based lamella network for enhanced sound absorption. *Adv. Funct. Mater.* **27**, 1703820 (2017).

**Comment 2:** *Other concerns are below.*

*Title: Anisotropic, Lamellar Ceramic Fiber Sponges with Temperature-Invariant High Compressibility from -196 to 1000°C*

*I think the title is misleading. In the present manuscript, the compressibility in liquid nitrogen is reported, while the compressibility of the samples after treatment under the high temperature conditions is reported (not under the high temperature conditions).*

**Response:** We thank the reviewer for the comments.

We agree with the reviewer. During the revision process, we applied our SAC sponges in the field of acoustic absorption and determined their sound absorption properties, therefore, we have changed the title to “Highly Compressible, Anisotropic, Lamellar Ceramic Sponges with Superior Thermal Insulation and Acoustic Absorption Performances”.

Corresponding changes:

Page 1: **Title: Highly Compressible, Anisotropic, Lamellar Ceramic Sponges with**

**Superior Thermal Insulation and Acoustic Absorption Performances**

**Reviewer #2**

*Authors have reported fabrication of highly compressible, temperature resistant SiO2-Al2O3 composite ceramic sponges via blow spinning a polyvinyl alcohol (PVA)-ceramic precursor solution and subsequent calcination, the detailed mechanical property of the resulting composite ceramic sponges has been performed and the thermal property of the obtained samples have been demonstrated. Temperature resistant (from liquid nitrogen temperature to 1000 degree C) ceramic materials have been found from literatures recently, and silica-alumina composite ceramics have been investigated deeply. Authors have reported compressible silica-alumina composite ceramics in their manuscript, but only given similar results reported from other ceramic materials. In such a way, it seems that the analysis in this manuscript is superficial.*

**Response:** We thank the reviewer for reading and understanding our manuscript.

*The detailed comments are as follows:*

**Comment 1:** *What is the role of the PVA in this process? Serving as the molecular template*

*for forming the microfibers? What is the interaction among PVA, TEOS and AlCl3?*

**Response:** We thank the reviewer for the comments.

In our work, PVA was used as a tackifier for tetraethyl orthosilicate (TEOS) and aluminum chloride (AlCl3) to promote the gelation process and also used as a template for forming the microfibers during the blow spinning process.

We obtained the FTIR spectra of PVA, TEOS, AlCl3 and PVA-TEOS-AlCl3 spinning solution to investigate the interaction between the solution components (Supplementary Fig. 2). TEOS shows characteristic peaks at 1167 and 787 cm^-1^, which correspond to the asymmetric stretching and symmetric stretching vibrations of Si-O-C bonds. TEOS will be hydrolyzed in the spinning solution. We added some H3PO4 in the spinning solution to promote the hydrolysis reaction. During the hydrolysis process, Si-O-Si bond networks are formed due to the hydrolysis and condensation reactions of TEOS molecules, which is demonstrated by the disappearance of Si-O-C bonds at 1167 and 787 cm^-1^ and the presence of Si-O-Si bond at 1044 and 876 cm^-1^. A new weak peak can be found at 1130 cm^-1^ in the spectrum of the spinning solution, which can be ascribed to the formation of new Si-O-C bond between PVA and TEOS through crosslinking.

Compared with the spinning solution without AlCl3, the PVA-TEOS-AlCl3 spinning solution becomes transparent and spinnable in a shorter time. As shown in Supplementary Fig. 3, the spinning solutions with some AlCl3 become transparent in 15 minutes, while the SiO2 precursor solution remains an emulsion state in 3 hours.

Corresponding changes:

Page 5: In our work, PVA was used as a tackifier for TEOS and AlCl3 to promote the gelation

process and also used as a template for forming the microfibers during the blow spinning

process. TEOS was hydrolyzed under the catalysis of H3PO4 in the spinning solution, which can be confirmed by the Fourier transform infrared spectra analysis (Supplementary Fig. 2 and Supplementary Discussion). We can obtain transparent and spinnable solutions quickly when adding some AlCl3 in the solutions. As shown in Supplementary Fig. 3, the spinning solutions with some AlCl3 become transparent in 15 minutes, while the SiO2 precursor solution without AlCl3 remains an emulsion state in 3 hours. Therefore, the addition of AlCl3 can greatly shorten the preparation time of spinning solutions.

Page 22: Infrared (IR) spectra were recorded with a FTIR spectrometer (VERTEX 70v, Bruker) at room temperature.

Page 7 in Supplementary Information:

**Supplementary Figure 2.** FTIR spectra of PVA, TEOS, AlCl3 and PVA-TEOS-AlCl3 spinning solution.

Page 8 in Supplementary Information:

**Supplementary Figure 3.** Optical images of the spinning solutions with different molar ratio of Al and Si (From left to right: 0:1, 0.1:1, 0.2:1, 0.4:1 and 0.8:1) at (a) 0 min, (b) 15 min, (c) 30 min, (d) 1 h, (e) 2 h, (f) 3 h after all chemicals were added.

Page 48 in Supplementary Information:

**Supplementary Discussion**

**Interaction between the spinning solution components**

We obtained the FTIR spectra of PVA, TEOS, AlCl3 and PVA-TEOS-AlCl3 spinning solution to investigate the interaction between the solution components. TEOS shows characteristic peaks at 1167 and 787 cm^-1^, which correspond to the asymmetric stretching and symmetric stretching vibrations of Si-O-C bonds. TEOS will be hydrolyzed in the spinning solution. We added some H3PO4 in the spinning solution to promote the hydrolysis reaction. During the hydrolysis process, Si-O-Si bond networks are formed due to the hydrolysis and condensation reactions of TEOS molecules, which is demonstrated by the disappearance of Si-O-C bonds at 1167 and 787 cm^-1^ and the presence of Si-O-Si bond at 1044 and 876 cm^-1^. A new weak peak can be found at 1130 cm^-1^ in the spectrum of the spinning solution, which can be ascribed to the formation of new Si-O-C bond between PVA and TEOS through crosslinking^26^.

Page 52 in Supplementary Information: Supplementary References:

26.	Pirzada, T., Arvidson, S. A., Saquing, C. D., Shah, S. S., Khan, S. A. Hybrid silica-PVA
nanofibers via sol-gel electrospinning. *Langmuir.* **28**, 5834-5844 (2012).

**Comment 2:** *What is the specific role for AlCl3? In the absence of the AlCl3, the products did*

*not show good flexibility. Does it ascribe to molecular mechanism or structural mechanism? If we tune the mass ratios of TEOS to AlCl3, what will we observe?*

**Response:** We thank the reviewer for the comments.

We added AlCl3 in the spinning solutions for several reasons. First, AlCl3 was used as the precursor of Al2O3 component in our sponges, and the presence of Al2O3 can inhibit the crystallization of SiO2. The pure SiO2 microfibers begin to crystallize at 1200°C (Supplementary Fig. 12), while the initial crystallization temperature of SiO2-Al2O3 composite microfibers is enhanced to 1300°C (Fig. 5j). Therefore, the addition of AlCl3 can increase the flexibility of our sponges obtained at higher calcination temperature. Secondly, for the SiO2 sponges without adding AlCl3 in the silica precursor solution, the deposited SiO2 fibers are easily blown away during the spinning process, which leads to a random fiber distribution. When adding some AlCl3 in the spinning solutions, the concentration of the solutions increases, which causes a larger fiber diameter (Supplementary Fig. 33). The microfibers with significantly increased diameter can be confined in a specific area and cannot be blown away, thus anisotropic lamellar sponges are obtained. Therefore, one role of AlCl3 is to increase the concentration of spinning solution, thus obtaining an anisotropic lamellar structure of sponges. Thirdly, we can obtain transparent and spinnable solutions quickly when adding some AlCl3 in the solutions. As shown in Supplementary Fig. 3, the spinning solutions with some AlCl3 become transparent in 15 minutes, while the SiO2 precursor solution without AlCl3 remains an emulsion state in 3 hours. Therefore, the addition of AlCl3 can greatly shorten the preparation time of spinning solutions.

Corresponding changes:

Page 5: We can obtain transparent and spinnable solutions quickly when adding some AlCl3 in the solutions. As shown in Supplementary Fig. 3, the spinning solutions with some AlCl3 become transparent in 15 minutes, while the SiO2 precursor solution without AlCl3 remains an emulsion state in 3 hours. Therefore, the addition of AlCl3 can greatly shorten the preparation time of spinning solutions.

Page 13: Compared with the pure SiO2 sponges, the presence of Al2O3 can inhibit the

crystallization of SiO2. The pure SiO2 microfibers begin to crystallize at 1200°C (Supplementary Fig. 12), while the initial crystallization temperature of the SAC microfibers is enhanced to 1300°C (Fig. 5j). Therefore, the addition of AlCl3 can increase the flexibility of the sponges obtained at higher calcination temperature.

Page 17: For the SiO2 sponges without adding AlCl3 in the silica precursor solution, the deposited SiO2 fibers are easily blown away during the spinning process, which leads to a random fiber distribution. Note that the average diameter of the PVA-SiO2 composite microfibers is only 1.3 μm (Supplementary Fig. 10a). When adding some AlCl3 in the spinning solutions, the concentration of the solutions increases, which causes a larger fiber diameter. It can be observed that the diameter of the microfibers increases with the increasing AlCl3 content (Supplementary Fig. 33). The microfibers with significantly increased diameter can be confined in a specific area and cannot be blown away, thus anisotropic lamellar sponges are obtained.

Page 8 in Supplementary Information:

**Supplementary Figure 3.** Optical images of the spinning solutions with different molar ratio of Al and Si (From left to right: 0:1, 0.1:1, 0.2:1, 0.4:1 and 0.8:1) at (a) 0 min, (b) 15 min, (c) 30 min, (d) 1 h, (e) 2 h, (f) 3 h after all chemicals were added.

Page 17 in Supplementary Information:

**Supplementary Figure 12.** XRD patterns of the PVA-SiO2 composite microfibers and SiO2 microfibers obtained at different calcination temperatures.

Page 35:

**Figure 5j.** XRD patterns of the SAC sponges calcinated at 1000, 1100, 1200, and 1300°C for 1 h.

Page 15 in Supplementary Information:

**Supplementary Figure 10**. Diameter distribution of (a) PVA-SiO2 composite microfibers and (b) SiO2 microfibers.

Page 38 in Supplementary Information:

**Supplementary Figure 33.** Optical images of the sponges prepared from spinning solutions with different molar ratio of Al and Si, and SEM images and diameter distribution of microfibers.

We prepared sponges from spinning solutions with different molar ratio of Al and Si. When adding some AlCl3 in the spinning solutions, we can obtain sponges with lamellar structure. It

can be observed that the diameter of the microfibers increases with the increasing AlCl3 content (Supplementary Fig. 33). We ascribe the lamellar structure of our SiO2-Al2O3 composite sponges to the significantly increased diameter, which is beneficial to the confinement of our sponges in a specific area. As shown in Supplementary Fig.34, all the SiO2-Al2O3 composite sponges prepared from spinning solutions with different Al and Si molar ratio show good compression resilience due to their anisotropic lamellar structure.

Corresponding changes:

Page 17: As shown in Supplementary Fig. 34, all the SAC sponges prepared from spinning solutions with different Al and Si molar ratio show good compression resilience.

Page 20: The spinning solution was prepared using the following procedures. First, 1.125 g PVA powder was dissolved in 10.8 g deionized (DI) water by magnetically stirring at 90°C for 1 h. 3.9 g TEOS, 0.0183 g phosphoric acid, and different masses of AlCl3·6H2O were subsequently added to the PVA solution. The mixed solutions were magnetically stirred at ambient temperature for approximately 2 h, yielding transparent solutions for preparing SAC sponges with Al and Si molar ratio of 0.1:1, 0.2:1, 0.4:1 and 0.8:1.

Unless otherwise specified, the SAC sponges used for characterization and determination were prepared from the spinning solutions with Al and Si molar ratio of 0.8:1.

Page 39 in Supplementary Information:

Supplementary Figure 34. Optical images of the sponges prepared from spinning solutions with different molar ratio of Al and Si to show their compression resilience.

In the absence of the AlCl3, the resulted SiO2 sponges also show good flexibility (Figure 3a). In our original manuscript, we have emphasized this point, as shown in Page 7, “The two sponges can be compressed beyond 80% strain thanks to their high flexibility and porosity.”. Compared with SAC sponges, the SiO2 sponges exhibit worse compression resilience, as shown in Figure 3. For the SiO2 sponges, the fibers in the sponges exhibit disordered distribution (Supplementary Fig. 9c). When the fibers are compressed with a large strain, the fibers with a large inclination angle cannot fully return to their original state due to the interference between the fibers. In addition, the SiO2 fibers have a smaller diameter (Supplementary Fig. 10b), and they are softer than SAC fibers, which is also responsible to the poor compressibility of the SiO2 sponges. We explained the flexibility mechanism of the fibers

through molecular dynamics simulation.

Corresponding changes:

Page 8: The flexibility mechanism of the SiO2 fibers and SAC fibers was investigated by molecular dynamics (MD) simulation (see details in Supplementary Methods). The modeling structures of the SiO2 fibers and SAC fibers with a size of 10×10×20 nm were built and stretched along *z* axis (Figs 3d and 3e). Figure 3f shows the tensile stress-strain curves of the two samples. From the results, the Young’s moduli of the SiO2 fibers and SAC fibers are found to be 142.0 GPa and 168.6 GPa, respectively. The tensile strength of the SiO2 fibers is 13.8 GPa and the corresponding tensile strain is up to 16.8%, demonstrating excellent flexibility. When the Al2O3 phase is added, the SAC fibers still possess a tensile strength of 11.6 GPa (corresponding to a tensile strain of 12.1%), which indicates that the SAC fibers also possess good ductility. In summary, the MD simulations suggest that the SAC fibers exhibit a higher Young’s modulus than that of SiO2 fibers, which can explain why the SAC sponges have better compression resilience. In addition, both SiO2 fibers and SAC fibers exhibit comparable ductility and flexibility.

Page 33:

**Figure 3. Flexibility and mechanical compressibility of the SiO2 and SAC sponges.** (a) Photographs of an SiO2 sponge before compression, during compression, and after release. (b) Photographs of an SAC sponge before compression, during compression, and after release. (c) Photographs showing the flexibility of the SAC sponges. The schematics in the top right corner of the photographs are used to show the folding and unfolding of the samples. Atomistic models of (d) SiO2 fiber and (e) SAC fiber. (f) Stress-strain curves from the molecular dynamics simulations of the SiO2 fibers and SAC fibers under uniaxial tension.

Page 46 in Supplementary Information:

**Molecular dynamics simulation**

For molecular dynamics (MD) simulation, Newton’s law was applied to describe the atomic interactions and MD was used to predict failure at the nanoscale by understanding the atomistic behavior of materials. MD simulations in this work were carried out using LAMMPS

(large-scale atomic/molecular massively parallel simulator), an open-source software package^22^, to investigate the mechanical properties of single fibers. The results were visualized using OVITO software (Open Visualization Tool)^23^.

**Force field**

In this work, the Matsui force field was selected for the structural analysis of the ceramic fibers at both high and low temperatures24, ^25^. The Born-Mayer-Huggins potential *V* was used to express the interaction between atoms, as shown in equation 3. The three parts in the force field equation represent Coulombic, repulsive, and van der Waals interactions, respectively.

*= + (1 + ) ((+−)*

(3)

*+	) −*

*^6^*

where *i* and *j* are the atom species, *qi* is the net charge on the atom *i*, *rij* is the distance between the atoms *i* and *j*, *f* is a constant (4.184 kJ/A mol), *Ai* is the repulsive radius for atom *i*, *Bi* is the softness parameter for atom *i*, *Ci* is the van der Waals coefficient for atom *i*. The relevant values are summarized in Supplementary Table 3.

**Supplementary Table 3.** Parameters of different elements for force field.

| Element | *q* (e) | *A* (Å) | *B* (Å) | *C* (*Å^3^ [/]^1^/^2^*) |
| --- | --- | --- | --- | --- |
| Al | 1.417 | 0.785 | 0.034 | 36.82 |
| Si | 1.890 | 0.720 | 0.023 | 49.30 |
| O | -0.945 | 1.821 | 0.138 | 90.61 |

**Molecular dynamics modeling of the tensile tests**

For the sake of simplification, the molar ratio of Al and Si in the SiO2-Al2O3 composite was set at 1:1 during the simulation process. The SiO2 and SiO2-Al2O3 composite were made by quenching the crystal SiO2 and SiO2-Al2O3 samples. The crystal samples made of approximately 160,000 atoms (10×10×20 nm) were equilibrated at 5000 K for 20 picoseconds in an NVT (constant number of atoms, constant volume, and constant temperature) ensemble. Then the high temperature melts were quenched to 300 K continuously over a period of 40 picoseconds in an NPT (constant number of atoms, constant pressure, and constant temperature) ensemble under ambient pressure. Finally, the samples were relaxed at 300 K under atmospheric pressure for 10 picoseconds. The Nose-Hoover thermostat and barostat were used to control the temperature and pressure, respectively. The time step used for all simulations was

0.5 femtosecond. Periodic boundary conditions were applied in all directions.

The mechanical properties of the SiO2 fibers and SAC fibers were investigated by carrying

out uniaxial tensile tests on the two samples. The samples were uniformly loaded in the *z*

direction at 300 K at the strain rate of 2×10^8^/s until the maximum strain of 30% in an NPT

ensemble.

Page 52 in Supplementary Information:

Supplementary References:

22. Plimpton, S. Fast parallel algorithms for short-range molecular-dynamics. *J. Comput. Phys.* **117,** 1-19 (1995). Also see <http://lammps.sandia.gov>.
23. Stukowski, A. Visualization and analysis of atomistic simulation data with ovito-the open visualization tool. *Modell. Simul. Mater. Sci. Eng.* **18,** 015012 (2010).
24. Matsui, M. Molecular dynamics study of the structures and bulk moduli of crystals in the system CaO-MgO-Al2O3-SiO2. *Phys. Chem. Miner.* **23,** 345-353 (1996).
25. Adabifiroozjaei, E., Hart, J. N., Koshy, P., Mitchell, D. R. G., Sorrell, C. C. Mullite-glass and mullite-mullite interfaces: Analysis by molecular dynamics (MD) simulation and high-resolution tem. *J. Am. Ceram. Soc.* **101,** 428-439 (2018).

**Comment 3:** *What is the intrinsic flexibility mechanism of the silica-alumina composite ceramic sponges? How to avoid plastic deformation during stress? Is it ascribing to chemical or structural mechanism? Could authors give chemical or structural model to simulate? I remind authors to pay much attention to the joint points of the composite fibers.*

**Response:** We thank the reviewer for the comments.

We explained the flexibility mechanism of our sponges through molecular dynamics simulation, please see response for Comment 2.

In order to avoid plastic deformation during stress, we should control the strain of materials within a certain limit. For our SAC sponges, it’s better to compress them within 80% strain. Our sponges possess an anisotropic lamellar structure. Most microfibers are distributed horizontally in the single fiber layer in the SAC sponges (Figs 2d-e), and they show good

compression resilience (Figs 4g-j), which results in the excellent compression mechanical properties of the SAC sponges. In addition, because of the horizontal distribution of microfibers in the SAC sponges, their deformation during compression is relatively small, which is also an important reason for the small plastic deformation after the multi-cycle compression test.

Apart from the high Young’s modulus of the SAC fibers, the lap joints among fibers also play an important role on the excellent compressibility of the sponges. The fibers in the SAC sponges become straighter and the fiber layers become denser under compression (Fig. 4h). Many lap joints among fibers can be observed from the SAC sponges (Fig. 4h). When the stress releases, the SAC fibers repel each other at the lap joints, which combines with the high Young’s modulus of the fibers make them to fully return to their original shapes (Fig. 4i).

Corresponding changes:

Page 9: In addition, the stress-strain curves did not change obviously after 600 cycles, showing that the SAC sponges have excellent compression fatigue resistance and they do not show obvious plastic deformation.

Page 10: Many lap joints among fibers can be observed from the SAC sponges (Fig. 4h), and they play an important role on the compressibility of the sponges. When the stress releases, the SAC fibers repel each other at the lap joints, which combines with the high Young’s modulus of the fibers make them to fully return to their original shapes (Fig. 4i).

Page 34:

**Figure 4. Mechanical properties of the SAC sponges under compression.** (a) Photographs of the multi-cycle compression test. (b) Compressive stress-strain curves during loading-unloading cycles with different maximum strain. (c) Compressive stress-strain curves at 50% maximum strain and different numbers of cycles. (d) Energy loss coefficient △U/U of the SAC sponges at different numbers of cycles. (e) Maximum stress retention during 600 cycles at 50% maximum strain. (f) Poisson’s ratio of the SAC sponges *versus* strain. (g-i) *In-situ* SEM images of the SAC sponges (g) before compression, (h) during compression, and (i) after release. The ellipses in (h) show the lap joints between fibers. (j) Schematic showing the morphological changes of the microfibers in the SAC sponges during a compression cycle.

**Comment 4:** *Please measure the thermal conductivity along different directions to see whether*

*samples possess anisotropic thermal property.*

**Response:** We thank the reviewer for the comments.

According to the suggestion of the reviewer, we determined the thermal conductivity of the sponge with a density of 16 mg cm^-3^ in the direction parallel to the fiber layer (Fig. 5b). The thermal conductivity slowly increases from 0.059 W _m_-1 K-1 to 0.091 W _m_-1 K-1 when the temperature increases from 20°C to 120°C. Compared with the thermal conductivity vertical to the fiber layer direction, the sponges show higher thermal conductivity in the direction parallel to the fiber layer, demonstrating anisotropic thermal property of our sponges. We ascribe the anisotropic thermal property of the sponges to the anisotropic lamellar structure, and the air within and between fiber layers can effectively inhibit the heat transport.

Corresponding changes:

Page 11: We also demonstrated the anisotropic thermal property of the SAC sponges by determining the thermal conductivity of the SAC sponges with a density of 16 mg cm^-3^ along different fiber layer directions. The thermal conductivity along different directions was found to increase as the temperature increased from 20 to 120°C (Fig. 5b), which can be attributed to the increased thermal radiation from trapped air at higher temperature^44^. The thermal conductivity in the direction vertical to the fiber layer slowly increases from 0.035 W _m_-1 _K_-1 to 0.046 W _m_-1 K-1 when the temperature increases from 20°C to 120°C. Compared with the thermal conductivity vertical to the fiber layer direction, the SAC sponges show higher thermal conductivity in the direction parallel to the fiber layer. We ascribe the anisotropic thermal property of the SAC sponges to the anisotropic lamellar structure, and the air within and between fiber layers can effectively inhibit the heat transport.

Page 35:

Figure 5b. Thermal conductivity of the SAC sponges with a density of 16 mg cm^-3^ along different fiber layer directions at different temperatures.

**Comment 5:** *Home-made hot-wire method has been used to measure the thermal conductivity. Please report the calibration results of authors home-made apparatus in the revised manuscript. In addition, hot-wire only gives the in-plane thermal conductivity of the samples, it is not suitable for the samples reported by authors. I suggest authors give the out-of-plane thermal conductivity result.*

**Response:** We thank the reviewer for the comments.

We reconfirmed the test method of the thermal conductivity of our sponges with the test engineer at the Department of Energy and Power Engineering at Tsinghua University. The thermal conductivities of our materials were actually determined using a thermal conductivity analyser (TPS 2500S, Hot Disk Instruments, Sweden) with the transient plane source method according to the testing standard of [ISO 22007-2:2015.](https://www.iso.org/standard/61190.html) The thermal conductivity analyser is shown in Fig. R1 and Supplementary Fig. 24. In the thermal conductivity determination system, the thermal constants analyser was used to determine the thermal conductivity of our samples (Supplementary Fig. 24a). The temperature was regulated via an oven (Supplementary Fig. 24b) and the test probe was placed between two sponge samples (Supplementary Fig. 24c). We apologize for the mistake caused by the communication problem with the test engineer. We

have corrected the determination method in the Characterization section in our revised manuscript. In addition, we have added the test engineer in our paper for his contribution to the thermal conductivity determination.

The thermal conductivity results from Hot Disk TPS 2500S thermal conductivity analyser are out-of-plane thermal conductivity, which can be confirmed from the specifications of this instrument (<https://www.hotdiskinstruments.com/products-services/instruments/tps-2500-s/).>

Corresponding changes:

Page 10: The thermal conductivities of the SAC sponges were determined using a Hot Disk thermal conductivity analyser with the transient plane source method according to the testing standard of [ISO 22007-2:2015](https://www.iso.org/standard/61190.html) (Supplementary Fig. 24).

Page 21: The thermal conductivity of the sponges was measured on a thermal conductivity analyser (TPS 2500S, Hot Disk Instruments, Sweden) with the transient plane source method according to the testing standard of [ISO 22007-2:2015.](https://www.iso.org/standard/61190.html)

Figure R1. Optical image of the thermal conductivity determination system, which includes

thermal constants analyser, an oven, and a computer.

Page 29 in Supplementary Information:

**Supplementary Figure 24.** Optical image of the thermal conductivity determination system. (a) Optical image of the thermal constants analyser, (b) optical image of the oven for temperature control, and (c) optical image of the test probe and SAC sponge samples.

**Comment 6:** *In the sentence “which is comparable to those of other inorganic aerogels”, why authors compare the physical properties of their samples with those of aerogels? A lot of such comparison can be seen. What is the relationship between sponge and aerogel? Why not compare those with that of any other spongy materials or other lightweight materials?*

**Response:** We thank the reviewer for the comments.

In our original manuscript, we compared the properties of our material with aerogels based on the following reasons. First, our material has similar microstructures with many reported aerogels, including SiC nanowire aerogel^1^, boron nitride aerogel^2^, SiC/SiOx aerogel^3^, and so on. Secondly, our material possesses similar properties with aerogels, including low density (10 mg cm^-3^), excellent flexibility and compressibility (retained 88.3% initial maximum stress after 600 compression cycles), and low thermal conductivity (0.034 W _m_-1 K-1 at room temperature). Finally, our material can be potentially applied in some fields where aerogels are commonly used, such as thermal insulation, water treatment, catalyst carriers and energy absorption. In our manuscript, we demonstrated the excellent thermal insulation and acoustic

absorption performance of our material. Therefore, comparing the properties of our material with aerogels should be appropriate.

In addition, we mainly compared our material with inorganic materials that can withstand high temperature and have a porous structure in our manuscript. Currently, most published literatures define a material with these properties as aerogel. Actually, according to the definition of [International Union of Pure and Applied Chemistry](https://www.baidu.com/link?url=Ny6kmMRd0qtjWHi4nsF_qo2B2ft3b3bjCPUVLHPG1Vi&wd=&eqid=ab7ef7e60025254c000000035d84760a) (IUPAC), aerogel is defined as a gel comprised of a microporous solid in which the dispersed phase is a gas^4^. According to the definition of aerogel, there are a large number of micropores in aerogels. We think that the materials presented in some papers are porous lightweight materials rather than aerogels. Therefore, we actually compared our material with some porous sponge-like materials and porous lightweight materials in our manuscript.

Actually, in our original manuscript, we compared our SAC sponges with some sponge-like materials, for example, Page 7, “The significantly different compression of the SAC sponges along two orthogonal directions shows that the SAC sponges have a unique anisotropic mechanical property, which results from the anisotropic lamellar structure; many other types of sponge-like materials with anisotropic structures also exhibit the similar behavior3, 34-36.” Page 8, “The observed phenomena in the compressive stress-strain curves are similar to those reported for other sponge-like materials3, 28, 29, 37.”

**References:**

1. Su, L. et al. Ultralight, recoverable, and high-temperature-resistant SiC nanowire aerogel. *ACS Nano* **12,** 3103-3111 (2018).
2. Li, G. et al. Boron nitride aerogels with super‐flexibility ranging from liquid nitrogen temperature to 1000°C. *Adv. Funct. Mater.***,** 1900188 (2019).
3. Ren B*,* et al. Nanofibrous aerogel bulk assembled by cross-linked SiC/SiOx core-shell nanofibers with multifunctionality and temperature-invariant hyperelasticity. *ACS Nano* **13,** 11603-11612 (2019).
4. Alemán JV*,* et al. Definitions of terms relating to the structure and processing of sols, gels, networks, and inorganic-organic hybrid materials (IUPAC recommendations 2007). *Pure Appl.*

*Chem.* **79,** 1801-1829 (2007).

**Reviewer #3**

*The authors developed highly compressible, temperature resistant SiO2-Al2O3 composite ceramic sponges by blow spinning polyvinyl alcohol-ceramic precursor solution and subsequent calcination. The sponges have a unique anisotropic lamellar structure, which provides the sponges with excellent mechanical compressibility, high compression fatigue resistance and robust mechanical stability. In addition, the composite sponges possess robust fire resistance, temperature-invariant compression resilience and excellent thermal insulation property. The advantages of their preparation method have been highlighted by comparison with the most commonly used ceramic sponge preparation methods. The authors have presented a well written paper supported by numerous figures both in the paper and in the Supplemental Information. The properties of the sponges were thoroughly and satisfactorily characterized and discussed, and the results are impressive. Taking these points into account, I consider that the manuscript is of interest to the readers of Nature Communications and I recommend this work to be accepted for publication after minor revision.*

**Response:** We thank the reviewer for the positive comments.

**Comment 1:** *Are the sponges hydrophobic? I wonder if the sponges will absorb water in air? It will be more interesting to give some data about that.*

**Response:** We thank the reviewer for the comments.

We determined the hydrophobicity of the SAC sponges using a contact angle goniometer, and they show a water contact angle of 128° (Supplementary Fig. 32a), indicating that our sponges are not very hydrophilic. We placed our SAC sponges in ambient environment, and weighed them at different time to determine the weight retention rate. The weight of the samples remained stable at different times and no weight change can be observed (Supplementary Fig. 32b), indicating that our sponges do not absorb water in air. The moisture resistance property

of our sponges was further demonstrated by moisture absorption and desorption test (Supplementary Figs 32c and 32d, Supplementary Methods). The sponges were placed in the water mist produced by a commercial humidifier for 10 hours, and they were weighed at different times. The weight of the samples was increased by 51% after exposing them in a high-humidity atmosphere in 10 hours. The adsorbed water in the samples was desorbed quickly when keeping them in ambient environment with a relative humidity of 60% and the samples returned to their original weight in about 2 hours (Supplementary Fig. 32e), indicating that the SAC sponges possess good moisture resistance property. Water is mainly distributed on the surface of the fibers due to their hydrophobic property, which results in the rapid moisture desorption.

Corresponding changes:

Page 16: Moisture resistance property is important for the practical application of sound absorption materials. Our SAC sponges exhibit a water contact angle of 128° (Supplementary Fig. 32a), which can prevent them from swelling and maintain the stable acoustic absorption properties. We placed our SAC sponges in ambient environment, and weighed them at different time to determine the weight retention rate. The weight of the samples remained stable at different times and no weight change can be observed (Supplementary Fig. 32b), indicating that our sponges do not absorb water in air. The moisture resistance property of our sponges was further demonstrated by moisture absorption and desorption test (Supplementary Figs 32c and 32d, Supplementary Methods). The weight of the samples was increased by 51% after exposing them in a high-humidity atmosphere for 10 hours. The adsorbed water in the samples was desorbed quickly when keeping them in ambient environment with a relative humidity of 60% and the samples returned to their original weight in about 2 hours (Supplementary Fig. 32e), indicating that the SAC sponges possess good moisture resistance property. Water is mainly distributed on the surface of the fibers due to their hydrophobic property, which results in the rapid moisture desorption.

Page 22: Water contact angles (WCA) of the sponges were determined using a contact angle goniometer (JCY, Shanghai Fangrui Instrument Co., Ltd, China).

Page 37 in Supplementary Information:

**Supplementary Figure 32.** Moisture resistance property of the SAC sponges. (a) Water contact angle of the SAC sponges. (b) Weight retention rate of the SAC sponges kept at ambient environment for different time. (c) Optical image of the setup for the moisture resistance property determination at high-humidity atmosphere. (d) Optical image of the SAC sponge in the water mist produced by a commercial humidifier. (e) Moisture gain and desorption properties of the SAC sponges.

Page 45 in Supplementary Information:

**Supplementary Methods**

**Moisture resistance property determination**

The moisture resistance property of the sponges was characterized by placing them in the

outlet of a commercial humidifier for moisture absorption and then keeping them in an

atmosphere with a relative humidity of ~60% and a temperature of ~25°C for moisture

desorption. The water content (*w*) in the sponges was calculated using the following equation:

| *(%) = 2−1*  *1* | *× 100%*, where *m*1 is the initial weight of the samples, and *m*2 is the weight |
| --- | --- |

of the samples after moisture absorption.

**Comment 2:** *I'm very concerned about the stability of the sponges. Whether the sponges can maintain good compression resilience after a long-term storage, such as 1 or 2 months? The authors should give some data to prove this point.*

**Response:** We thank the reviewer for the comments.

We have qualitatively studied the mechanical compressibility of the SAC sponge with a density of 15 mg cm^-3^ stored at room temperature for 2 months, as shown in Supplementary Fig. 21. The sponges can be compressed beyond 80% strain and almost completely return to their initial state without obvious dimensional change after pressure release. In addition, quantitative study also shows that there is no obvious deterioration in the mechanical properties of the SAC sponge after long-term storage at room temperature (Supplementary Fig. 22). The SAC sponges maintain 96.9% of the initial maximum stress after 100 compression cycles (Supplementary Fig. 22b), indicating that our sponges can maintain good compression resilience after a long-term storage.

Corresponding changes:

Page 10: The mechanical compressibility of the SAC sponges with a density of 15 mg cm^-3^ stored at room temperature for 2 months was studied to evaluate their mechanical stability after a long-term storage. The sponges can be compressed beyond 80% strain and almost completely return to their initial state without obvious dimensional change after pressure release (Supplementary Fig. 21). In addition, quantitative study also shows that there is no obvious deterioration in the mechanical properties of the SAC sponge after long-term storage at room temperature. The SAC sponges maintain 96.9% of the initial maximum stress after 100 compression cycles (Supplementary Fig. 22), indicating that our sponges can maintain good compression resilience after a long-term storage.

Page 26 in Supplementary Information:

**Supplementary Figure 21.** Optical images of an SAC sponge stored at room temperature for 2 months, (a) before compression, (b) during compression, and (c) after release.

Page 27 in Supplementary Information:

**Supplementary Figure 22.** Mechanical compressibility of the SAC sponge stored at room temperature for 2 months. (a) Compressive stress-strain curves of the SAC sponge (with a density of 15 mg cm^-3^) after 2 months storage at 50% maximum strain for different numbers of cycles. (b) Maximum stress retention during 100 cycles at 50% maximum strain. (c) Energy loss coefficient △U/U of the SAC sponge at different numbers of cycles.

**Reviewer comments, second version:**

Reviewer #1 (Remarks to the Author):

I have carefully read the authors’ response letter and the revised manuscript. The authors have adequately responded to the reviewers’ comments. My additional concerns on acoustic adsorption properties are below.

Page 15-16, Lines 358-363: “We ascribe the excellent sound absorption properties of our SAC sponges to the following reasons. First, the SAC sponges possess a lamellar structure, which leads to multiple absorption of sound waves between layers (Fig. 7e). Second, the microfibers in our SAC sponges have a rough surface (Fig. 2c), which can increase the friction between sound waves and our materials, thus causing more consumption of sound energy during the sound propagation progress.”
Some researchers pointed out the effect of the vibration of nanofibers (thinner than 3 μm) induced by sound waves is substantial for sound absorption property (e.g., S. Akasaka et al., Applied Acoustics, 152, 13-20, 2019, DOI: 10.1016/j.apacoust.2019.03.016).
Do the SAC sponges composed of SiO2-Al2O3 thin fibers have a similar effect? The authors should make this point clear.


Reviewer #2 (Remarks to the Author):

Authors have revised their manuscript according to my comments, however their understandings on the concerns related to chemistry and mechanism are still superficial, the further revisions should be given before publish:
1)What the role of the PVA? Authors have claimed that PVA serving as the soft template as well as the tackifier, however no experimental evidences have been provided in the revised manuscript. If PVA can serve as the soft template, the cryo-TEM images or other observed results should be given in the manuscript to confirm it. If PVA can serve as the tackifier, the viscosity comparison or other comparisons before and after addition of PVA should be provided in the manuscript to confirm it. Only text description is not sufficient to convince readers.
2)What kind of the interactions among PVA, TEOS and AlCl3? Authors have just provided IR spectra to confirm the existence of the PVA, no interaction description could be found in the revised manuscript. According to my understanding, one or more than one of the interactions among hydrogen bonding, coordination bonding, donor-acceptor interaction, etc. should be existed among PVA, TEOS and AlCl3. Authors should design experiments to confirm these interactions and report their results in the revised manuscript.
3)What is the specific role of AlCl3? Authors have claimed that “We can obtain transparent and spinnable solutions quickly when adding some AlCl3 in the solutions”. But why? Why AlCl3 can make solution transparent? Please give the scientific explanation rather than non-scientific description in the revised manuscript. According to my understanding, AlCl3 might serve as the acidic catalyst to promote further hydrolysis of TEOS. Please design experiments to confirm it.
4)What is the intrinsic flexibility mechanism of the silica-alumina composite ceramic sponges? How to avoid plastic deformation during stress? Is it ascribing to chemical or structural mechanism? Could authors give chemical or structural model to simulate? Actually, I am not satisfied with the explanation authors made in the revised manuscript. Authors have done the additional MD simulation in the revised manuscript to explain above-mentioned concerns, but not appropriate as well as correct. According to MD simulation, individual SiO2/Al2O3 composite fibers process young’s modulus in the magnitude of GPa order, however the real sponges possess Young’s modulus in the magnitude of only KPa order, which shows the MD simulation is not appropriate and not suitable for the case in explanation on the flexibility of the resulting composite sponges. I do suggest authors use a finite element model to simulate and explain the flexibility of the resulting composite sponges in the revised manuscript.


Reviewer #3 (Remarks to the Author):

The authors described the fabrication of highly compressible, temperature resistant SiO2-Al2O3 composite ceramic sponges via blow spinning. And the authors have revised the manuscript carefully according to the reviewers’ comments. I am satisfied with the revised manuscript and the present form of the manuscript can be published.

**Author rebuttal, second version:**

*I have carefully read the authors’ response letter and the revised manuscript. The authors have adequately responded to the reviewers’ comments.*

**Response:** We thank the reviewer for the positive comments.

**Comment 1:** *My additional concerns on acoustic adsorption properties are below. Page 15-16, Lines 358-363: “We ascribe the excellent sound absorption properties of our SAC sponges to the following reasons. First, the SAC sponges possess a lamellar structure, which leads to multiple absorption of sound waves between layers (Fig. 7e). Second, the microfibers in our SAC sponges have a rough surface (Fig. 2c), which can increase the friction between sound waves and our materials, thus causing more consumption of sound energy during the sound propagation progress.” Some researchers pointed out the effect of the vibration of nanofibers (thinner than 3 μm) induced by sound waves is substantial for sound absorption property (e.g., S. Akasaka et al., Applied Acoustics, 152, 13-20, 2019, DOI: 10.1016/j.apacoust.2019.03.016). Do the SAC sponges composed of SiO2-Al2O3 thin fibers have a similar effect? The authors should make this point clear.*

**Response:** We thank the reviewer for the comments.

Per the reviewer’s suggestion, we carefully read the relevant literatures and further analyzed our data to explore the possible sound absorption mechanism of our SAC sponges. We found a sound absorption peak at the frequency of about 1 kHz in the

sound absorption curves (Figure 7b). The appearance of sound absorption peak can be attributed to the fiber vibration^1^. The resonant frequency of the SAC sponges at the absorption peak shows a tendency to move to a lower frequency with the increasing thickness, which can be ascribed to the increased areal density of the SAC sponges^2-4^. The fiber vibration caused by the sound waves may also play an important role in sound absorption.

Corresponding changes:

**Page 16:** We ascribe the excellent sound absorption properties of our SAC sponges to the following reasons. First, the SAC sponges possess a lamellar structure, which leads to multiple absorption of sound waves between layers (Fig. 7e). Second, the microfibers in our SAC sponges have a rough surface (Fig. 2c), which can increase the friction between sound waves and our materials, thus causing more consumption of sound energy during the sound propagation progress. Third, a sound absorption peak can be observed at the frequency of about 1 kHz in the sound absorption curves (Fig. 7b), which can be attributed to the fiber vibration^63^. The resonant frequency of the SAC sponges at the absorption peak shows a tendency to move to a lower frequency with the increasing thickness, which can be ascribed to the increased areal density of the SAC sponges^64-66^. The fiber vibration caused by the sound waves may also play an important role in sound absorption.

**Page 38:**

**Figure 7b.** Sound absorption coefficient of the SAC sponges with different thicknesses.

**References:**

1. Akasaka, S., Kato, T., Azuma, K., Konosu, Y., Matsumoto, H., Asai, S. Structure-sound absorption property relationships of electrospun thin silica fiber sheets: Quantitative analysis based on acoustic models. *Appl. Acoust.* **152,** 13-20 (2019).
2. Rabbi, A., Bahrambeygi, H., Nasouri, K., Shoushtari, A. M., Babaei, M. R. Manufacturing of PAN or PU nanofiber layers/PET nonwoven composite as highly effective sound absorbers. *Adv. Polym. Technol.* **33,** 21425 (2014).
3. Kalinova, K. Nanofibrous resonant membrane for acoustic applications. *J. Nanomater.* **2011,** 265720 (2011).
4. Avossa, J. et al. Light electrospun polyvinylpyrrolidone blanket for low frequencies sound absorption. *Chin. J. Polym. Sci.* **36,** 1368-1374 (2018).

**Reviewer #2**

*Authors have revised their manuscript according to my comments, however their understandings on the concerns related to chemistry and mechanism are still superficial, the further revisions should be given before publish:*

**Comment 1:** *What the role of the PVA? Authors have claimed that PVA serving as the soft template as well as the tackifier, however no experimental evidences have been provided in the revised manuscript. If PVA can serve as the soft template, the cryo-TEM images or other observed results should be given in the manuscript to confirm it. If PVA can serve as the tackifier, the viscosity comparison or other comparisons before and after addition of PVA should be provided in the manuscript to confirm it. Only text description is not sufficient to convince readers.*

**Response:** We thank the reviewer for the comments.

In our work, PVA was used to assist fiber forming during the blow spinning process, so we call PVA a template. Note that polymers have been widely used as template to prepare ceramic fibers^1-5^. PVA and ceramic precursors were uniformly mixed together in the spinning solution (Supplementary Fig. 1a), and PVA was decomposed and

removed from the fibers during the calcination process. The successful removal of PVA can be confirmed by the TGA result (Figure 5m). The uniform distribution of the ceramic components in the fibers can be confirmed by the elemental mapping images (Figure 2g). Therefore, from these results, we confirmed that PVA was used as a template to help shape the ceramic fibers.

**Supplementary Figure 1a.** Optical image of the PVA-TEOS-AlCl3 solution.

**Figure 5m.** Thermogravimetric analysis of the SAC sponges and PSAC sponges in air.

**Figure 2g.** SEM image of a microfiber in the SAC sponge and corresponding elemental mapping images of Si, Al, and O.

PVA was also used as a tackifier for TEOS and AlCl3 to promote the gelation process. PVA-TEOS-AlCl3 solution and TEOS-AlCl3 solution were prepared and their viscosity was determined using a rheometer (Supplementary Fig. 2). The PVA-TEOS-AlCl3 solution and TEOS-AlCl3 solution are transparent, but they show different viscosity. The viscosity of the PVA-TEOS-AlCl3 solution is much higher than that of the TEOS-AlCl3 solution due to the addition of PVA. We cannot obtain fibers from the TEOS-AlCl3 solution due to its low viscosity. Many studies have also shown that a low viscosity solution cannot yield fibers by spinning^6-8^. In addition, these results also confirmed that PVA was used as a template to assist fiber forming during the blow spinning process.

Corresponding changes:

**Page 5:** In our work, PVA was used as a tackifier for TEOS and AlCl3 to promote the gelation process and also used as a template for forming the microfibers during the blow spinning process (Supplementary Fig. 2 and Supplementary Discussion).

**Page 22:** The viscosity of the solutions was determined using a rheometer (Physica MCR 302, Anton Paar, Austria) in the shear rate range of 0.01-1000 s^-1^ at a constant temperature of 25°C.

**Page 7** in Supplementary Information:

**Supplementary Figure 2.** (a) Optical image and (b) viscosity curves of the PVA-TEOS-AlCl3 solution and TEOS-AlCl3 solution.

**Page 51** in Supplementary Information:

**Role of PVA in the spinning solution**

In our work, PVA was used as a tackifier for TEOS and AlCl3 to promote the gelation process and also used as a template for forming the microfibers during the blow spinning process. PVA was used to assist fiber forming during the blow spinning process, so we call PVA a template. Note that polymers have been widely used as template to prepare ceramic fibers^26-30^. PVA and ceramic precursors were uniformly mixed together in the spinning solution (Supplementary Fig. 1a), and PVA was decomposed and removed from the fibers during the calcination process. The successful removal of PVA can be confirmed by the TGA result (Figure 5m). The uniform distribution of the ceramic components in the fibers can be confirmed by the elemental mapping images (Figure 2g). Therefore, from these results, we confirmed that PVA was used as a template to help shape the ceramic fibers.

PVA was also used as a tackifier for TEOS and AlCl3 to promote the gelation process. PVA-TEOS-AlCl3 solution and TEOS-AlCl3 solution were prepared and their viscosity was determined using a rheometer (Supplementary Fig. 2). The PVA-TEOS-AlCl3 solution and TEOS-AlCl3 solution are transparent, but they show different viscosity. The viscosity of the PVA-TEOS-AlCl3 solution is much higher than that of the TEOS-AlCl3 solution due to the addition of PVA. We cannot obtain fibers from the TEOS-AlCl3 solution due to its low viscosity. Many studies have also shown that a low

viscosity solution cannot yield fibers by spinning^31-33^. In addition, these results also confirmed that PVA was used as a template to assist fiber forming during the blow spinning process.

**References:**

1. Yan, J. et al. Polymer template synthesis of flexible BaTiO3 crystal nanofibers. *Adv. Funct. Mater.* **29,** 1907919 (2019).
2. Yan, J. et al. Polymer template synthesis of soft, light, and robust oxide ceramic films. *iScience* **15,** 185-195 (2019).
3. Wang, Y., Li, W., Jiao, X., Chen, D. Electrospinning preparation and adsorption properties of mesoporous alumina fibers. *J. Mater. Chem. A* **1,** 10720-10726 (2013).
4. Wang, X., Dou, L., Li, Z., Yang, L., Yu, J., Ding, B. Flexible hierarchical ZrO2 nanoparticle-embedded SiO2 nanofibrous membrane as a versatile tool for efficient removal of phosphate. *ACS Appl. Mater. Interfaces* **8,** 34668-34676 (2016).
5. Li, D., Xia, Y. N. Fabrication of titania nanofibers by electrospinning. *Nano Lett.* **3,** 555-560 (2003).
6. Gupta, P., Elkins, C., Long, T. E., Wilkes, G. L. Electrospinning of linear homopolymers of poly(methyl methacrylate): Exploring relationships between fiber formation, viscosity, molecular weight and concentration in a good solvent. *Polymer* **46,** 4799-4810 (2005).
7. Vicini, S., Mauri, M., Vita, S., Castellano, M. Alginate and alginate/hyaluronic acid membranes generated by electrospinning in wet conditions: Relationship between solution viscosity and spinnability. *J. Appl. Polym. Sci.* **135,** 46390 (2018).
8. Nezarati, R. M., Eifert, M. B., Cosgriff-Hernandez, E. Effects of humidity and solution viscosity on electrospun fiber morphology. *Tissue Eng., Part C* **19,** 810­819 (2013).

**Comment 2:** *What kind of the interactions among PVA, TEOS and AlCl3? Authors have just provided IR spectra to confirm the existence of the PVA, no interaction description could be found in the revised manuscript. According to my understanding, one or more*

*than one of the interactions among hydrogen bonding, coordination bonding, donor-acceptor interaction, etc. should be existed among PVA, TEOS and AlCl3. Authors should design experiments to confirm these interactions and report their results in the revised manuscript.*

**Response:** We thank the reviewer for the constructive comments.

According to the reviewer’s suggestion, we further explored the possible interaction among the components in the PVA-TEOS-AlCl3 solution via ^27^Al and ^29^Si nuclear magnetic resonance (NMR) spectroscopy. A sharp peak at 0.3 ppm can be observed in the ^27^Al spectrum (Supplementary Fig. 4), which can be ascribed to non-bonded but partly hydrolyzed, octahedral-coordinated Al ions^1-4^. The results indicate that Al^3+^ was hydrolyzed in the spinning solution, and octahedral-coordinated Al ions ([Al(H2O)6]^3+^) were formed4, ^5^. The partly hydrolyzed but non-bonded Al ions will form associates connected by weak hydrogen bonds^1-3^. In addition, the very weak peak at 70.2 ppm can be attributed to tetrahedral coordinated Al3, ^4^, and the amount of tetrahedral coordinated Al is very small.

The ^29^Si spectrum of the PVA-TEOS-AlCl3 solution shows three peaks at -107.8, -102.1 and -92.5 ppm. The peaks at -92.5 and -102.1 ppm correspond to the silicon of geminal silanol and single silanol, respectively^6^, which proves the presence of Si-OH bond. The peak at -107.8 ppm can be attributed to the silicon of siloxane^7^, which demonstrates the existence of Si-O-Si bond. These results indicate that TEOS hydrolyzes in the spinning solution. In addition, compared with pure silica gel7, ^8^, the ^29^Si spectrum of the PVA-TEOS-AlCl3 solution shows a little of chemical shift, which can be ascribed to the formation of Si-O-Al bond^6^.

In addition, we further analyzed the FTIR spectrum of the PVA-TEOS-AlCl3 solution (Supplementary Fig. 3), and a wide and weak peak at 717 cm^-1^ was found, which can be ascribed to Si-O-Al vibration5, ^9^. Al^3+^ from AlCl3 can react with partially hydrolyzed TEOS to occupy the proton sites of silanol and form Si-O-Al bond^10^. In summary, Al^3+^ exist mainly in the form of [Al(H2O)6]^3+^ in the spinning solution, and a small amount of Al^3+^ reacted with TEOS and formed Si-O-Al bond.

Corresponding changes:

**Page 5:** TEOS was hydrolyzed under the catalysis of H3PO4 in the spinning solution, and the interactions among PVA, TEOS and AlCl3 were examined by the Fourier transform infrared spectra and ^27^Al, ^29^Si nuclear magnetic resonance (NMR) spectra analysis (Supplementary Fig. 3, Supplementary Fig. 4 and Supplementary Discussion). **Page 22:** ^27^Al and ^29^Si nuclear magnetic resonance (NMR) measurement was performed on a 600MHz NMR spectrometer (AVANCE NEO, Bruker, Germany) at room temperature.

**Page 8** in Supplementary Information:

**Supplementary Figure 3.** FTIR spectra of PVA, TEOS, AlCl3 and PVA-TEOS-AlCl3

spinning solution.

**Page 9** in Supplementary Information:

**Supplementary Figure 4.** (a) ^27^Al and (b) ^29^Si NMR spectra of the PVA-TEOS-AlCl3

solution. The inset in (a) is the magnified NMR spectrum in the range of 20-120 ppm.

**Page 52** in Supplementary Information:

In addition, a wide and weak peak at 717 cm^-1^ was found, which can be ascribed to Si-O-Al vibration35, ^36^. Al^3+^ from AlCl3 can react with partially hydrolyzed TEOS to occupy the proton sites of silanol and form Si-O-Al bond^37^.

We further explored the possible interaction among the components in the PVA-TEOS-AlCl3 solution via ^27^Al and ^29^Si nuclear magnetic resonance (NMR) spectroscopy. A sharp peak at 0.3 ppm can be observed in the ^27^Al spectrum (Supplementary Fig. 4), which can be ascribed to non-bonded but partly hydrolyzed, octahedral-coordinated Al ions^38-41^. The results indicate that Al^3+^ was hydrolyzed in the spinning solution, and octahedral-coordinated Al ions ([Al(H2O)6]^3+^) were formed35, 41. The partly hydrolyzed but non-bonded Al ions will form associates connected by weak hydrogen bonds^38-40^. In addition, the very weak peak at 70.2 ppm can be attributed to tetrahedral coordinated Al40, ^41^, and the amount of tetrahedral coordinated Al is very small.

The ^29^Si spectrum of the PVA-TEOS-AlCl3 solution shows three peaks at -107.8, -102.1 and -92.5 ppm. The peaks at -92.5 and -102.1 ppm correspond to the silicon of geminal silanol and single silanol, respectively^42^, which proves the presence of Si-OH bond. The peak at -107.8 ppm can be attributed to the silicon of siloxane^43^, which demonstrates the existence of Si-O-Si bond. These results also indicate that TEOS hydrolyzes in the spinning solution. In addition, compared with pure silica gel43, ^44^, the ^29^Si spectrum of the PVA-TEOS-AlCl3 solution shows a little of chemical shift, which can be ascribed to the formation of Si-O-Al bond^42^.

**References:**

1. Sinkó, K., Mezei, R., Zrinyi, M. Gelation of aluminosilicate systems under different chemical conditions. *J. Sol-Gel Sci. Technol.* **21,** 147-156 (2001).
2. Sinkó, K., Neményi, A. Effect of Al- and Si-containing precursors on the structures
   formed by sol-gel methods. *Prog. Colloid Polym. Sci.* **125,** 103-110 (2004).
3. Sinkó, K., Mezei, R., Rohonczy, J., Fratzl, P. Gel structures containing Al (III). *Langmuir.* **15,** 6631-6636 (1999).
4. Komarneni, S., Roy, R., Fyee, C. A., Kennedy, G. J., Strobl, H. Solid-state ^27^Al and ^29^Si magic-angle spinning NMR of aluminosilicate gels. *J. Am. Ceram. Soc.* **69,** C-42-C-44 (1986).
5. Wu, X., Shao, G., Cui, S., Wang, L., Shen, X. Synthesis of a novel Al2O3-SiO2 composite aerogel with high specific surface area at elevated temperatures using inexpensive inorganic salt of aluminum. *Ceram. Int.* **42,** 874-882 (2016).
6. Sato, S., Maciel, G. E. Structures of aluminum chloride grafted on silica surface. *J. Mol. Catal. A: Chem.* **101,** 153-161 (1995).
7. Chuang, I.-S., Kinney, D. R., Bronnimann, C. E., Zeigler, R. C., Maciel, G. E. Effects of 1H-1H spin exchange in the ^29^Si CP-MAS NMR spectra of the silica surface. *J. Phys. Chem.* **96,** 4027-4034 (1992).
8. Maciel, G. E., Sindorf, D. W. Silicon-29 nuclear magnetic resonance study of the surface of silica gel by cross polarization and magic-angle spinning. *J. Am. Chem. Soc.* **102,** 7606-7607 (1980).
9. Ling, X., Li, B., Li, M., Hu, W., Chen, W. Thermal stability of Al-modified silica aerogels through epoxide-assisted sol-gel route followed by ambient pressure drying. *J. Sol-Gel Sci. Technol.* **87,** 83-94 (2018).
0. Chakrabarti, S., Pramanik, P. Binary silicate gels using tetraethyl orthosilicate and weak acid salts. *J. Mater. Sci. Lett.* **9,** 1135-1136 (1990).

**Comment 3:** *What is the specific role of AlCl3? Authors have claimed that “We can obtain transparent and spinnable solutions quickly when adding some AlCl3 in the solutions”. But why? Why AlCl3 can make solution transparent? Please give the scientific explanation rather than non-scientific description in the revised manuscript. According to my understanding, AlCl3 might serve as the acidic catalyst to promote further hydrolysis of TEOS. Please design experiments to confirm it.*

**Response:** We thank the reviewer for the constructive comments.

We prepared PVA-TEOS solution and PVA-TEOS-AlCl3 solution, and determined their pH using pH indicator papers to confirm the reviewer’s opinion. We found that the pH significantly decreases from ~5 to ~1 when added some AlCl3 in the solution (Supplementary Fig. 5). Al^3+^ from AlCl3 can react with partially hydrolyzed TEOS to release some hydrochloric acid^1^, and the strong acid significantly reduces the pH of PVA-TEOS-AlCl3 solution. Some studies have shown that the hydrolysis rate of TEOS increases with decreasing pH in the acidic range^2^. In addition, AlCl3 is a well-known strong acid catalyst, and has been widely used in various organic reactions^3^. Therefore, AlCl3 can serve as an acidic catalyst to promote the hydrolysis of TEOS in our work. The addition of AlCl3 significantly reduces the hydrolysis time of TEOS, so we can obtain a transparent solution in a shorter time.

Corresponding changes:

**Page 5:** We can obtain transparent and spinnable solutions quickly when adding some AlCl3 in the solutions because AlCl3 can also serve as an acidic catalyst to promote the hydrolysis of TEOS (Supplementary Fig. 5 and Supplementary Discussion).

**Page 10** in Supplementary Information:

**Supplementary Figure 5.** pH determination of the PVA-TEOS solution and PVA-TEOS-AlCl3 solution. (a, b) Optical images of the PVA-TEOS solution and pH indicator paper impregnated with the solution. (c, d) Optical images of the PVA-TEOS-

AlCl3 solution and pH indicator paper impregnated with the solution. (e) Optical image of the colourimetric card.

**Page 53** in Supplementary Information:

**Role of AlCl3 serving as an acidic catalyst**

We prepared PVA-TEOS solution and PVA-TEOS-AlCl3 solution, and determined their pH using pH indicator papers. We found that the pH significantly decreases from ~5 to ~1 when added some AlCl3 in the solution (Supplementary Fig. 5). Al^3+^ from AlCl3 can react with partially hydrolyzed TEOS to release some hydrochloric acid^37^, and the strong acid significantly reduces the pH of PVA-TEOS-AlCl3 solution. Some studies have shown that the hydrolysis rate of TEOS increases with decreasing pH in the acidic range^45^. In addition, AlCl3 is a well-known strong acid catalyst, and has been widely used in various organic reactions^42^. Therefore, AlCl3 can serve as an acidic catalyst to promote the hydrolysis of TEOS in our work. The addition of AlCl3 significantly reduces the hydrolysis time of TEOS, so we can obtain a transparent solution in a shorter time.

**References:**

1. Chakrabarti, S., Pramanik, P. Binary silicate gels using tetraethyl orthosilicate and weak acid salts. *J. Mater. Sci. Lett.* **9,** 1135-1136 (1990).
2. Cihlář, J. Hydrolysis and polycondensation of ethyl silicates. 1. Effect of pH and catalyst on the hydrolysis and polycondensation of tetraethoxysilane (TEOS). *Colloids Surf., A* **70,** 239-251 (1993).
3. Sato, S., Maciel, G. E. Structures of aluminum chloride grafted on silica surface. *J.
   Mol. Catal. A: Chem.* **101,** 153-161 (1995).

**Comment 4:** *What is the intrinsic flexibility mechanism of the silica-alumina composite ceramic sponges? How to avoid plastic deformation during stress? Is it ascribing to chemical or structural mechanism? Could authors give chemical or structural model to simulate? Actually, I am not satisfied with the explanation authors made in the revised manuscript. Authors have done the additional MD simulation in the revised*

*manuscript to explain above-mentioned concerns, but not appropriate as well as correct. According to MD simulation, individual SiO2/Al2O3 composite fibers process young’s modulus in the magnitude of GPa order, however the real sponges possess Young’s modulus in the magnitude of only KPa order, which shows the MD simulation is not appropriate and not suitable for the case in explanation on the flexibility of the resulting composite sponges. I do suggest authors use a finite element model to simulate and explain the flexibility of the resulting composite sponges in the revised manuscript.*

**Response:** We thank the reviewer for the comments and apologize for the misunderstanding in the last revision. We believe this point can be explained clearly using the theory of mechanics of cellular materials. The superior flexibility of cellular materials or aerogels has been discovered since the 1990s. It is well accepted that the extremely low apparent elastic moduli yet exceptional compressibility of these materials compared to their bulk counterparts originate from the nonlinear structural response of the high-aspect-ratio ligaments, such as bending and buckling. The study by Schaedler *et al.*^1^ provided a good summary on the relationships between the relative modulus (*E*/*E*s, defined as the ratio between the apparent modulus of the cellular material *vs.* that of the bulk material) and the relative density (*ρ*/*ρ*s, defined as the ratio between the apparent density of the cellular material *vs.* that of the bulk material). They have pointed out that strong interactions between the ligaments lead to a scaling law of *E*/*E*s~(*ρ*/*ρ*s)^2^. In contrast, if the interactions between ligaments are weak (which is the case for our sponges), a scaling law of *E*/*E*s~(*ρ*/*ρ*s)^3^ emerges (see Figure R1 below adapted from the paper by Schaedler *et al.*). Therefore, we can use the scaling law to predict the elastic modulus of our sponges. The density of the SiO2-Al2O3 ceramic is *ρ*s=2.6 g cm^-3^, and the density of the sponges ranges from 10 to 30 mg cm^-3^ (*i.e.*, *ρ*/*ρ*s ranges from 3.8×10^-3^ to 1.1×10^-2^ in our study). From the chart below, we get *E*/*E*s ranging from 5×10^-8^ to 8×10^-7^. Inputting *E*s=168.6 GPa from our MD simulations, the scaling law predicts that an apparent elastic modulus around 8 kPa to 130 kPa, comparable very well to the tangential moduli from the stress-strain curves of our sponges (Figure 4b and 4c in the main text). Therefore, the flexibility of the sponges

can be well explained by the MD simulation combined with the theoretical analysis.

**Figure R1.** Scaling laws for the relative compressive modulus (*E*/*E*s) vs. relative density (*ρ*/*ρ*s) for different types of cellular materials (Adopted from the paper by Schaedler *et al.*).

Corresponding changes:

**Page 9:** Previous studies suggest a scaling law of *E*/*E*s~(*ρ*/*ρ*s)^3^ (where the properties of bulk constitutive solids are denoted by subscript “s”) for cellular materials consisting of weakly interacting ligaments^37^. Herein, we have *ρ*s=2.6 g cm^-3^ for the SiO2-Al2O3 ceramic and *ρ* in the range of 10-30 mg cm^-3^ for the SAC sponges. Inputting *E*s=168.6 GPa for SAC fibers obtained from MD simulations, the scaling law suggests that our sponges should have an elastic modulus of approximately 8 kPa to 130 kPa, which is comparable to the tangential moduli of our sponges from the stress-strain curves in Fig. 4b.

**References:**

1.	Schaedler, T. A. et al. Ultralight metallic microlattices. *Science* **334,** 962-965 (2011).

**Reviewer #3**

*The authors described the fabrication of highly compressible, temperature resistant
SiO2-Al2O3 composite ceramic sponges via blow spinning. And the authors have revised*

*the manuscript carefully according to the reviewers’ comments. I am satisfied with the revised manuscript and the present form of the manuscript can be published.* **Response:** We thank the reviewer for the comments.

**Reviewer comments, third version:**

Reviewer #1 (Remarks to the Author):

The manuscript should be published in Nature Communications.


Reviewer #2 (Remarks to the Author):

I am satisfied with the corrections authors made this time and thus suggest it publish in its current state.

**Author rebuttal, third version:**
